# Driving the Emission Towards Blue by Controlling the HOMO‐LUMO Energy Gap in BF_2_‐Functionalized 2‐(Imidazo[1,5‐*a*]pyridin‐3‐yl)phenols

**DOI:** 10.1002/chem.202101520

**Published:** 2021-07-12

**Authors:** Gioele Colombo, G. Attilio Ardizzoia, Julien Furrer, Bruno Therrien, Stefano Brenna

**Affiliations:** ^1^ Department of Science and High Technology University of Insubria Via Valleggio, 9 22100 Como Italy; ^2^ Consorzio Interuniversitario Reattività Chimica e Catalisi (CIRCC) Bari Italy; ^3^ Department für Chemie Biochemie und Pharmazie Universität Bern Freiestrasse 3 3012 Bern Switzerland; ^4^ Institute of Chemistry Université de Neuchâtel Avenue de Bellevaux 51 2000 Neuchâtel Switzerland

**Keywords:** boron, blue emission, fluorescence, HOMO-LUMO gap, TD-DFT

## Abstract

Several boron compounds with 2‐(imidazo[1,5‐*a*]pyridin‐3‐yl)phenols, differentiated by the nature of the substituent (R) in the para position of the hydroxy group, have been synthesized and thoroughly characterized both in solution (^1^H, ^13^C, ^11^B, ^19^F NMR) and in the solid state (X‐ray). All derivatives displayed attractive photophysical properties like very high Stokes shift, high fluorescence quantum yields and a good photostability in solution. Time‐Dependent Density Functional Theory (TD‐DFT) calculations allowed to define the main electronic transitions as intra ligand transitions (^1^ILT), which was corroborated by the Natural Transition Orbitals (NTOs) shapes. The homo‐lumo energy gap was correlated to the electronic properties of the substituent R on the phenolic ring, as quantified by its σ_p_ Hammett constant.

## Graphical abstract

**Blue emissive materials**: boron containing fluorescent compounds have been prepared starting from imidazo[1,5‐*a*]pyridine phenols. Large Stokes shift, high absolute quantum yields and good photostability are observed in solution. TD‐DFT calculations allowed to find a linear correlation between the HOMO‐LUMO energy gap of these compounds and the Hammett constants of substituents R para to the hydroxyl group of the phenols.

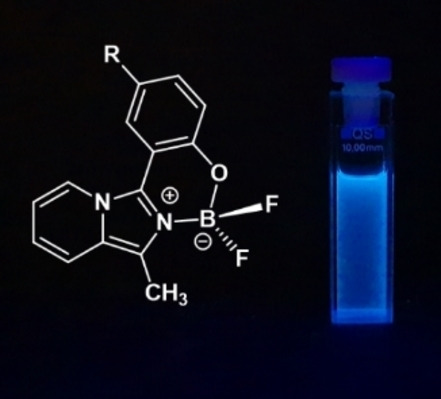

## Introduction

In recent years there has been a growing attention towards luminescent compounds suitable for different devices such as Organic Light Emitting Diodes (OLEDs),^[1]^ Light Emitting Electrochemical Cells (LECs),^[2]^ Dye Sensitized Solar Cells (DSSCs)^[3]^ or as fluorescent sensors.^[4]^ These compounds are mostly based on rare and expensive transition metals, like platinum(II),^[5]^ iridium(III),^[6]^ ruthenium(II)^[7]^ or rhenium(I)^[8]^ and, therefore, not convenient from an economic point of view. In order to offer a valid alternative to these efficient but high‐cost compounds, the interest has been moved to low‐cost organic dyes^[9]^ and to coordination compounds of cheaper metals, usually in a d^10^ electronic configuration, such as copper(I)^[10]^ or zinc(II).[^11^, ^12^] Boron is also another interesting element which is known to give fluorescent coordination compounds,[^13^, ^14^, ^15^] with boron dipyrromethene (BODIPY) being the most renown and studied derivatives.^[16]^ Thanks to their photophysical properties, these species are known to play a significant role in numerous application fields, ranging from biomolecular probes^[17]^ to optoelectronic.^[18]^ Even more interesting is the possibility to tune the emission of these species, by introducing small modifications[^19^, ^20^] and/or extending the conjugation around the fluorescent core.^[21]^ BODIPY type species are extremely versatile as well, thanks to their high photo^[22]^ and chemical stability^[23]^ and their high solubility even in aqueous media.^[24]^ Other classes of ligands, either N,N^[25]^ or N,O^[26]^ chelating, proved to be useful in the synthesis of highly emissive boron compounds as well.

Among our research group, we also focused on a series of organic dyes, namely the imidazo[1,5‐*a*]pyridines, whose biological^[27]^ and photophysical properties^[28]^ have been largely studied and reported in the literature, the latter including large Stokes shifts and high quantum yields.[^29^, ^30^] Furthermore, they are known to act as ligands in the synthesis of luminescent transition‐metal coordination compounds,[^31^, ^32^, ^33^, ^34^, ^35^] or in the fabrication of OLEDs.[^36^, ^37^, ^38^] In previous works, the photophysical properties of this class of coordination compounds were investigated on both zinc(II),[^39^, ^40^, ^41^] and silver(I),^[42]^ using opportunely derivatized imidazo[1,5‐*a*]pyridine ligands. More recently, a series of hydrogenated dyes deriving from this class of molecules has also been studied and demonstrated to be efficient blue emissive materials in solution and in thin film.^[43]^ Surprisingly, only few examples of boron derivatives with imidazo‐pyridines have been reported in the literature.[^44^, ^45^] In the recent past, we also investigated the previously cited hydrogenated ligands in the synthesis of boron difluoride coordination compounds, which showed excellent optical properties like high fluorescence quantum yields.^[46]^

For all these reasons, we decided to prepare a series of new boron compounds, using imidazo[1,5‐*a*]pyridines functionalized with a phenolic ring, with various substituents R para to the hydroxyl group. The photophysical properties of this class of boron containing compounds are described, focusing especially on the correlation between their electronic properties and their fluorescent behavior.

## Results and Discussion

### Syntheses and characterization

**Scheme 1:**
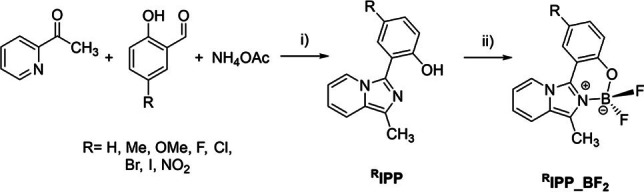
Synthesis of ^**R**^ **IPP** and reaction with boron trifluoride diethyletherate to obtain ^**R**^ **IPP_BF_2_**. i) 2‐Acetylpyridine:aldehyde:NH_4_OAc 1 : 2 : 5; AcOH, r.t., 7 days. ii) BF_3_⋅Et_2_O, Et_3_N, CH_2_Cl_2_, r.t.

(Imidazo[1,5‐*a*]pyridin‐3‐yl)phenols ^**R**^ **IPP** were prepared following published methods,^[47]^ by condensation of 2‐acetylpyridine and the corresponding 5‐substituted salicylaldehydes, in the presence of ammonium acetate as a source of the imidazolic nitrogen (Scheme 1). The boron difluoride coordination compounds were obtained by reaction of ^**R**^ **IPP** with boron trifluoride diethyl etherate, in dichloromethane at room temperature, in the presence of triethylamine (Scheme 1). The purity of the products was confirmed by elemental analysis and solution ^1^H and ^13^C{^1^H} NMR spectroscopy.

When R=H, a partially hydrated species ^**H**^ **IPP_BF_2_** ⋅ ***x*** **H_2_O** was obtained, as also confirmed by the TGA analysis conducted on one batch (Figure S1, Supporting Information). The water content cannot be exactly quantified since ^**H**^ **IPP_BF_2_** ⋅ ***x*** **H_2_O** is indeed a mixture of two products, as resulted from the analysis of X‐ray powder spectra (see Figure S2–S3). In diluted solutions ^**H**^ **IPP_BF_2_** ⋅ ***x*** **H_2_O** easily loses water and ^**H**^ **IPP_BF_2_** is recovered as the only product after removal of the solvent. The purity of the latter was verified by comparison of its X‐ray powder diffraction pattern with the one calculated from the CIF file of the related X‐ray single crystal structure (see below).

^1^H NMR spectra of ^**R**^**IPP_BF_2_** recorded in CD_2_Cl_2_ (Figures S4–S35) exhibit as expected several resonances belonging to the aromatic protons between 6.50 and 8.50 ppm, and a singlet between 2.40 and 2.70 ppm ascribed to the methyl group on the imidazolic ring. The corresponding resonances in the ^13^C NMR spectra were identified between 116 and 132 ppm (aromatic CH) and at about 9–10 ppm for the methyl group (Figures S4–S35). The ^19^F NMR spectra (CD_2_Cl_2_, 25 °C) exhibit a non‐binomial quartet around −140 ppm, with a coupling constant ^1^ *J*_F‐B_≈15 Hz due to the coupling with ^11^B. The small shoulder appearing downfield from the main resonance was attributed to the resonance of ^19^F bound to ^10^B (unresolved *J*‐coupling). The possibility that this shoulder could also result from a resonance due to inequivalent fluorine atoms could be ruled out by low temperature experiments: in the ^19^F NMR spectrum of ^**H**^ **IPP_BF_2_** recorded at −25 °C (Figure S36), this additional shoulder is not visible anymore. Even if there is a general broadening of the resonances, no second resonance that would speak for a rotamer is visible. The ^11^B NMR spectra (Supporting Information) exhibit a triplet centered around 0.5–1.0 ppm, with the ^1^ *J*_F‐B_≈15 Hz. The fact that a real triplet and not a doublet of doublet is observed for the ^11^B NMR resonance fully agrees with the expected tetrahedral geometry at the boron center, confirmed by X‐ray crystal structure analysis.

The infrared spectra (ATR) support the NMR data, showing among other a series of bands between 1000 and 1100 cm^−1^, consistent with the presence of BF_2_ derivatives (Figures S37–S45).

### X‐ray crystal structure analysis

**Figure 1:**
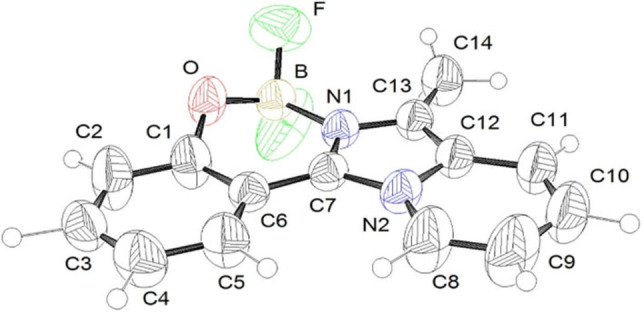
ORTEP representation of ^**H**^ **IPP_BF_2_** at 50 % probability level ellipsoids, with atom labeling scheme.

Compounds ^**H**^ **IPP_BF_2_** and ^**OMe**^ **IPP_BF_2_** were characterized by single‐crystal X‐ray structure analysis (Figure 1 and Figure S46).^[48]^ As expected, the boron center shows a tetrahedral geometry, with the (imidazo[1,5‐*a*]pyridine‐3‐yl)phenol entity chelating the boron atom in a *N,O*‐bidentate mode. In the crystal of ^**OMe**^ **IPP_BF_2_** two independent molecules, labeled A and B, are observed (Figure S46). In both compounds, bond distances and angles are comparable with those reported for similar systems (Table 1).[^44^, ^45^] The structure of the H‐derivative shows a nearly planar arrangement between the imidazolyl ring and the phenolic residue, with an angle between these planes of 0.000(4)°. On the other hand, for the methoxy derivative, the same angles measure 20.3(1)° (molecule A) and 18.2(1) (molecule B) respectively, thus revealing a higher degree of distortion from planarity for this **IPP**‐derivative.

**Table 1:** Selected bond lengths (Å) and angles (°) for ^**H**^ **IPP_BF_2_** and ^**OMe**^ **IPP_BF_2_** (molecules A and B).

	^**H**^ **IPP_BF_2_**	^**OMe**^**IPP_BF_2_** (molecule A)^[a]^	^**OMe**^**IPP_BF_2_** (molecule B)^[a]^
B−N(1) B−O B−F(1) B−F(2)	1.578(5) 1.440(6) 1.337(3) 1.337(3)	1.577(8) 1.446(8) 1.393(8) 1.370(7)	1.581(8) 1.440(8) 1.394(7) 1.380(8)
N(1)−B−O N(1)−B−F(1) N(1)−B−F(2) F(1)−B−O F(2)−B−O F(1)−B−F(2)	108.5(3) 109.3(3) 109.3(3) 125.3(4) 125.3(4) 106.8(4)	107.4(5) 108.3(5) 109.6(5) 111.3(5) 109.8(5) 110.4(5)	107.2(5) 109.4(5) 109.1(5) 108.7(5) 112.6(5) 109.7(5)

[a] See Supporting Information (Figure S46).

### Optical properties

All ^**R**^ **IPP_BF_2_** derivatives show an intense fluorescence in solution. First, in order to evaluate any possible solvent effect on the photophysical properties of these compounds, we recorded the absorption, excitation and emission spectra of ^**H**^ **IPP_BF_2_** in various solvents with different polarity (Table 2).

**Table 2:** Photophysical data for compound ^**H**^ **IPP_BF_2_** recorded in solution (5 ⋅ 10^−5^ M) in different solvents.^[a]^

Solvent	λ_abs_	λ_exc_	λ_em_	Stokes shift	φ_PL_	τ
CH_2_Cl_2_	347	348	446	0.77	0.21	3.5
acetone	347	352	445	0.74	0.21	3.8
CH_3_CN	346	352	445	0.74	0.15	3.9
EtOH	346	347	441	0.76	0.22	3.9
THF	348	348	449	0.80	0.15	4.0
toluene	351	352	450	0.77	0.19	3.8
AcOEt	347	347	446	0.79	0.13	3.7
CHCl_3_	348	348	448	0.80	0.11	3.2
DMF	349	353	446	0.73	0.24	4.4

[a] λ_abs,_ λ_exc_, λ_em_: nm; Stokes shift: eV; τ: ns.

The absorption spectra show very similar characteristics, with a main absorption at about 345–350 nm and a second one at 235–245 nm (Figure S47, Table 2). The spectra recorded in acetone, *N*,*N*‐dimethylformamide, ethyl acetate and toluene did not present the second absorption band, due to the strong absorption of the solvent in that range (Figure S47). The excitation spectra largely reproduce the UV‐Vis spectra, being comparable with each other as well. The emission spectra are characterized by a broad emission band centered at about 447 nm (Figure S47, Table 1). The emission spectrum recorded in toluene showed a more structured band, due to the vibronic structure. In any case, compound ^**H**^ **IPP_BF_2_** is always characterized by a bright blue fluorescence emission, without experiencing any interesting shifting (range from 441 to 450 nm) of the maxima. In all cases, mono‐exponential lifetime decays are observed, with τ values in the range of 3.2–4.4 ns (Table 2). Also, fluorescence quantum yields are very similar (Table 2) and no dramatic quenching was observed in these solvents. The other ^**R**^ **IPP_BF_2_** derivatives have a similar behavior, as demonstrated by the absorption and emission spectra recorded in different solvents for ^**Me**^ **IPP_BF_2_** and ^**Cl**^ **IPP_BF_2_** (Figures S48–S49). Regardless the solvent, for these compounds the emission is always centered respectively at 450 (^**Me**^ **IPP_BF_2_**) and 438 (^**Cl**^ **IPP_BF_2_**) nm. Also, absolute quantum yields and lifetime decays (see Tables S2‐S3 in Supporting Information) are analogous in the various solvents. The only exception in the ^**R**^ **IPP_BF_2_** series is represented by the NO_2_‐substitued compound, which indeed shows a significant dependence of the emission from the solvent used (Figure S50 and Table S4). As shown below, the topology of the NTOs describing the low energy excitation transitions can explain this behavior.

Based on the aforesaid observations, we continued our investigation on the photophysical properties of ^**R**^ **IPP_BF_2_** compounds in solution using dichloromethane, which represents the best choice in terms of solvent power and photoluminescent performances.

**Figure 2:**
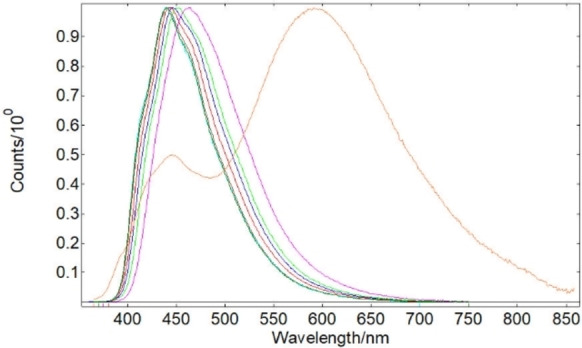
Normalized emission spectra of ^**R**^ **IPP_BF_2_** compounds recorded in solution (CH_2_Cl_2_, 5 ⋅ 10^−5^ M). (▪), ^**H**^ **IPP_BF_2_**; (▪), ^**Me**^ **IPP_BF_2_**; (▪), ^**MeO**^ **IPP_BF_2_**; (▪), ^**F**^ **IPP_BF_2_**; (▪), ^**Cl**^ **IPP_BF_2_**; (▪), ^**Br**^ **IPP_BF_2_**; (▪), ^**I**^ **IPP_BF_2_**; (▪), ^**NO2**^ **IPP_BF_2_**. (checked, it's ok)

The normalized UV‐vis spectra of ^**R**^ **IPP_BF_2_** recorded in solution (CH_2_Cl_2_, 5 ⋅ 10^−5^ M) (Figure S51, Supporting Information) are characterized by two main absorption bands, respectively centered at about 240 nm and 345–365 nm, depending on the substituent. ^**NO2**^ **IPP_BF_2_** also showed one additional absorption at 307 nm, with lower intensity with respect to the others. The normalized excitation and emission spectra recorded in solution (CH_2_Cl_2_, 5 ⋅ 10^−5^ M) for all ^**R**^ **IPP_BF_2_** derivatives are reported in Figure S52 (Supporting Information): the excitation spectra largely reproduce the absorption traces; all the emission maxima (Figure 2, Table 3) are positioned in the deep blue region of the visible spectrum (440–463 nm). As documented by the corresponding CIE 1931 plot (Figure S53), (*x*,*y*) color coordinates for ^**R**^ **IPP_BF_2_** are very close to those expected for standard blue (0.16, 0.10).^[49]^ The only exception is represented by the nitro‐substituted derivative ^**NO2**^ **IPP_BF_2_**, characterized by a λ_max_ centered in the orange region (597 nm) and a second maximum with lower intensity at 445 nm. To clarify the origin of this dual emission we recorded the excitation spectra of both emission bands (Figure S54 in Supporting Information). They are the same, and this rules out the presence of two different ground states. Thus, as also observed for other fluorescent dual emitters reported in the literature,^[50]^ the dual emission of ^**NO2**^ **IPP_BF_2_** can be described to originate from ^1^π‐π* (short‐wavelength) and ^1^CT transitions (long‐wavelength). This is consistent with the analysis of Natural Transition Orbitals (see below), showing that the long‐wavelength emission dominates, in accordance with the presence of solvent effect observed for ^**NO2**^ **IPP_BF_2_**.

**Table 3:** Photophysical data for boron difluoride compounds ^**R**^ **IPP_BF_2_** in dichloromethane solution (5 ⋅ 10^−5^ M).^[a]^

R	λ_abs_	ϵ	λ_exc_	λ_em_	Shift	φ_PL_	τ	*k*_r_	*k*_nr_
H	347	17580	348	446	0.77	0.21	3.5	6.2	2.2
Me	352	18710	351	452	0.79	0.19	3.5	5.4	2.3
OMe	362	17320	365	463	0.72	0.16	3.7	4.3	2.3
F	357	15950	356	443	0.68	0.22	3.5	6.2	2.2
Cl	358	13510	356	442	0.68	0.23	4.1	5.6	1.9
Br	358	16890	357	440	0.66	0.22	4.1	5.4	1.9
I	359	15750	361	440	0.62	0.09	1.5	6.2	6.2
NO_2_	350	7310	356	597	0.80	<0.05	1.2^[b]^	–	–

[a] λ_abs,_ λ_exc_, λ_em_: nm; ϵ: M^−1^⋅cm^−1^; Stokes shift: eV; τ: ns; *k*_r_: ⋅ 10^7^ s^−1^; *k*_nr_: ⋅ 10^8^ s^−1^. [b] Biexponential decay, weighted average between τ_1_=1.1 ns (relative percentage 95 %) and τ_2_=3.0 ns (relative percentage 5 %).

In general, for ^**R**^ **IPP_BF_2_** compounds, λ_max_ is influenced by the substituent R para to the hydroxyl group of the phenolic ring, moving to higher wavelengths with increasing the electron donating character of the substituent (i. e., from 440 for ^**Br**^ **IPP_BF_2_** and ^**I**^ **IPP_BF_2_** to 463 for ^**OMe**^ **IPP_BF_2_**). Worthy of note is the large Stoke shift (0.62–0.80 eV) observed for all ^**R**^ **IPP_BF_2_** compounds (Table 3), with a very small superposition between the excitation and the emission spectra. All these compounds showed a fluorescence behavior, with mono‐exponential lifetime decays in the range of 3.5–4.1 ns (Table 3 and Figure S55, Supporting Information). Absolute quantum yields are quite good for almost all the compounds described (Table 3), which is a desired property when combined with large Stokes shift, since the latter are often associated with a lower emission efficiency.^[51]^ The lower quantum yields are observed as expected for ^**NO2**^ **IPP_BF_2_**, due to the known quenching effect of nitro groups,^[52]^ and ^**I**^ **IPP_BF_2_**, this last reasonably experiencing a heavy‐atom effect. ^**I**^ **IPP_BF_2_** also showed the shortest lifetime decay of just 1.5 ns (Table 3); on the other hand, ^**NO2**^ **IPP_BF_2_** was the only derivative showing a biexponential lifetime decay, with a weighted value of 1.2 ns (Table 3).

A requested feature for dyes to be used in energy‐efficiency devices is a good optical stability. Therefore, we performed photostability measurements in dichloromethane solution for compound ^**H**^ **IPP_BF_2_**, taken again as a representative example of the whole series. A multiple emission scan analysis (Figure S56) was performed over a period of two hours (100 scans, 72 seconds each scan with no delay between scans). No significative loss in the emission intensity was detected (Δ_scan1‐scan100_=ca. 0.84 %) (Figure S57).

### DFT calculations

**Figure 3:**
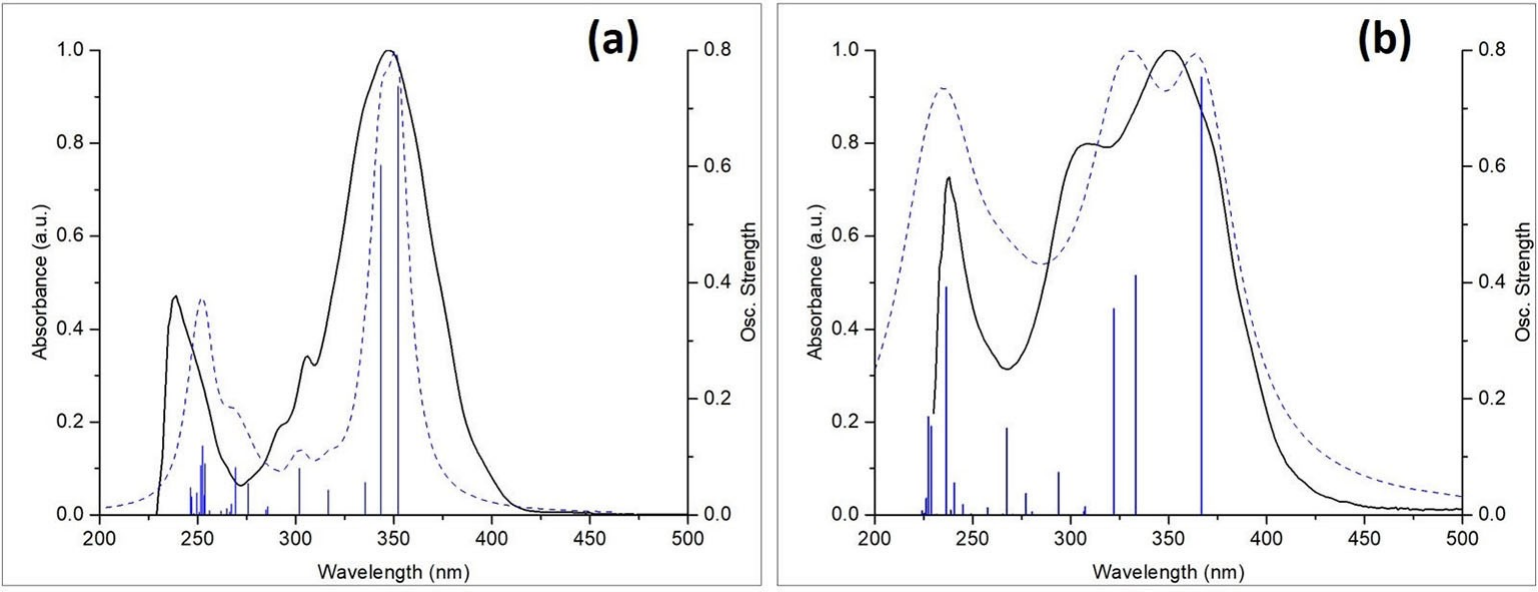
Calculated (dashed line) vs. experimental (full line) UV‐vis traces in dichloromethane solution: (a) ^**H**^ **IPP_BF_2_**, (b) ^**NO2**^ **IPP_BF_2_**.

The X‐ray crystal structure of compound ^**H**^ **IPP_BF_2_** was used as the starting point to obtain the ground state (S_0_) optimized geometries of all ^**R**^ **IPP_BF_2_** derivatives at the DFT/PBE‐D3 level of theory. Calculated and experimental (X‐ray) bond distances and angles for ^**H**^ **TIPP_BF_2_** and ^**OMe**^ **TIPP_BF_2_** are in good agreement. TD‐DFT calculations were also useful to determine the nature of the transitions responsible for the absorption processes. Figure 3 proves the good accordance between the calculated (dashed line) and the experimental (full line) UV‐vis spectra in solution (CH_2_Cl_2_) both for ^**H**^ **IPP_BF_2_** and ^**NO2**^ **IPP_BF_2_**. Vertical bars represent calculated transitions with oscillator strength *f*>0.1. The contributions of single orbital transitions to the absorption at lower energy are reported in Table 4.

**Table 4:** Contributions of single orbital transitions for ^**H**^ **IPP_BF_2_** and ^**NO2**^ **IPP_BF_2_**.

^**R**^ **IPP_BF_2_**	**NTOs**		
^**H**^ **IPP_BF_2_**	NTO1 NTO2	HOMO→LUMO HOMO‐1→LUMO HOMO→LUMO +1 HOMO‐2→LUMO+1 HOMO→LUMO+1 HOMO→LUMO HOMO‐1→LUMO HOMO‐1→LUMO+1 HOMO→LUMO+2	81.3 % 7.5 % 5.3 % 1.3 % 83.5 % 8.1 % 2.1 % 2.0 % 1.3 %
^**NO2**^ **IPP_BF_2_**	NTO1 NTO2 NTO3	HOMO→LUMO+1 HOMO‐1→LUMO HOMO→LUMO HOMO‐1→LUMO HOMO‐3→LUMO+2 HOMO→LUMO+2	98.0 % 1.5 % 93.2 % 3.5 % 1.1 % 89.7 %

**Figure 4:**
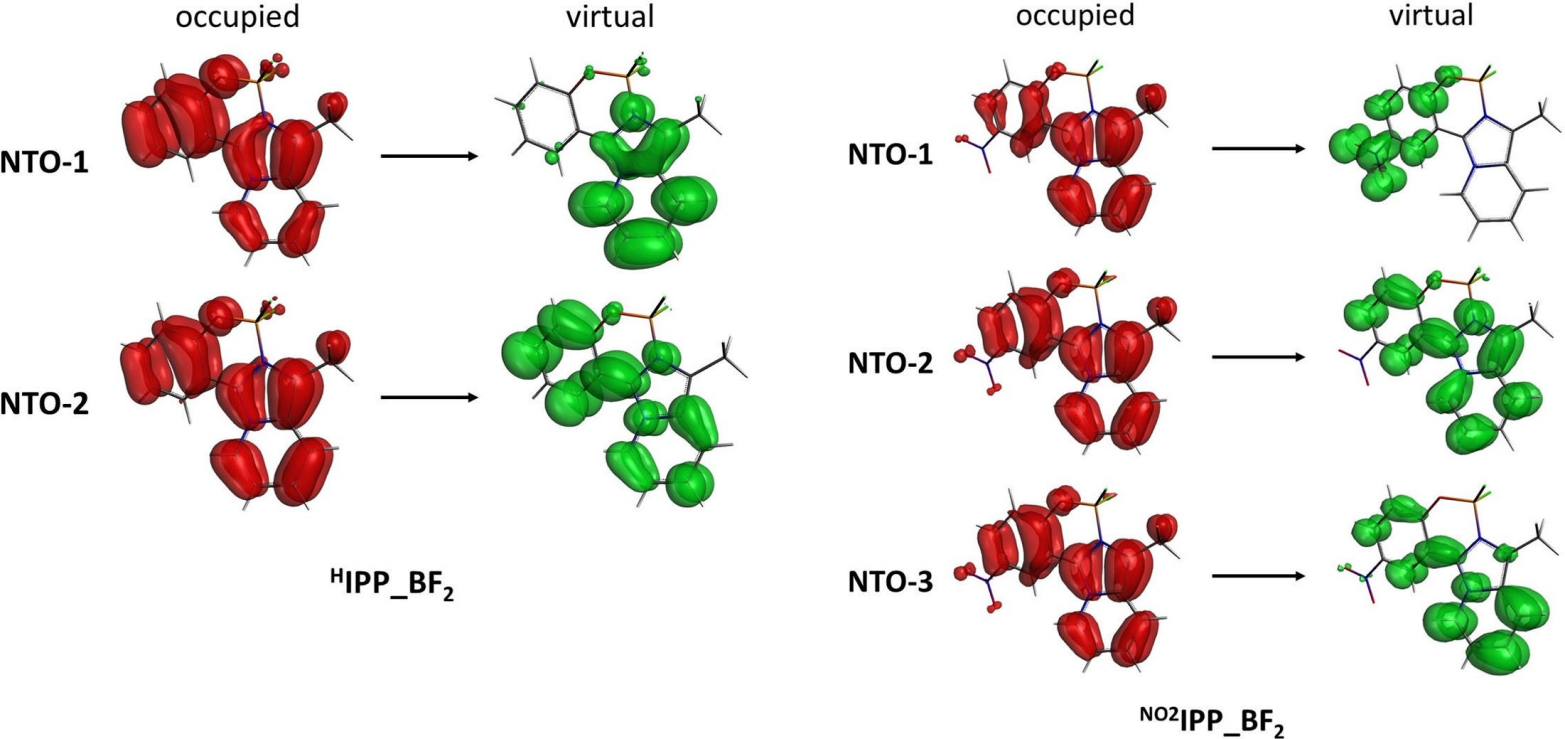
Natural Transition Orbitals (NTOs) calculated for compounds ^**H**^ **IPP_BF_2_** and ^**NO2**^ **IPP_BF_2_** relative to the first (NTO‐1), second (NTO‐2) and (for ^**NO2**^ **IPP_BF_2_**) third (NTO‐3) low energy transitions (see text).

For ^**H**^ **IPP_BF_2_** and nearly all other ^**R**^ **IPP_BF_2_** compounds, the absorption centered at about 350 nm is composed of two electronic transitions (Figure 3, (a)), mainly having a homo‐lumo (81.3 % of first transition) or homo‐lumo+1 (83.5 % of second transition) character. Compound ^**NO2**^ **IPP_BF_2_** is characterized by a different UV‐vis spectrum (Figure 3, (b)), where three calculated transitions are observed in the range 330–350 nm, mostly described as homo‐lumo (98.0 %), homo‐lumo+1 (93.2 %) and homo‐lumo+2 (89.7 %) (Table 4). These contributions to single orbital transitions are associated to Natural Transition Orbitals (NTOs) reported in Figure 4 (see Figures S58–S59 for NTOs for all ^**R**^ **IPP_BF_2_**). They show similar shapes among the ^**R**^ **IPP_BF_2_** series (R=H, Me, OMe, F, C, Br, I), with occupied NTOs (red contour) distributed over the entire molecule (NTO1 and NTO2), and the virtual NTOs (green contour) mainly localized on the imidazo[1,5‐*a*]pyridine portion (as expected acting as the acceptor moiety) (NTO1) or also involving the phenol residue (NTO2). In ^**NO2**^ **IPP_BF_2_**, all occupied NTOs are spread over the entire imidazo[1,5‐*a*]pyridine phenol system, whereas virtual NTOs are localized on the phenol ring (NTO1) or on the entire molecule (NTO2 and NTO3). In all cases, no contribution is observed from the BF_2_ fragment, which ultimately enhances the fluorescence of the free (imidazo[1,5‐*a*]pyridine‐3‐yl)phenols by giving more rigidity to the whole system.

In conclusion, the lower energy absorption band in ^**R**^ **IPP_BF_2_** (R=H, Me, OMe, F, Cl, Br, I) is due to the contribution of two close vertical transitions, the first with a clear (highlighted by NTO1) and the second with a negligible (highlighted by NTO2) charge transfer character. Plausibly, the latter transition dominates in accordance with the absence of solvent effect experimentally observed. On the contrary, for ^**NO2**^ **IPP_BF_2_** the lower energy absorption band is due only to a single ^1^CT transition (evidenced by NTO1), thus explaining the solvent dependence.

We then performed geometry optimization of the S_1_ excited state for ^**R**^ **IPP_BF_2_**: worthy of note, the computed structures for S_1_ match those of S_0_, with only very slight deviations (Figure S60).

**Figure 5:**
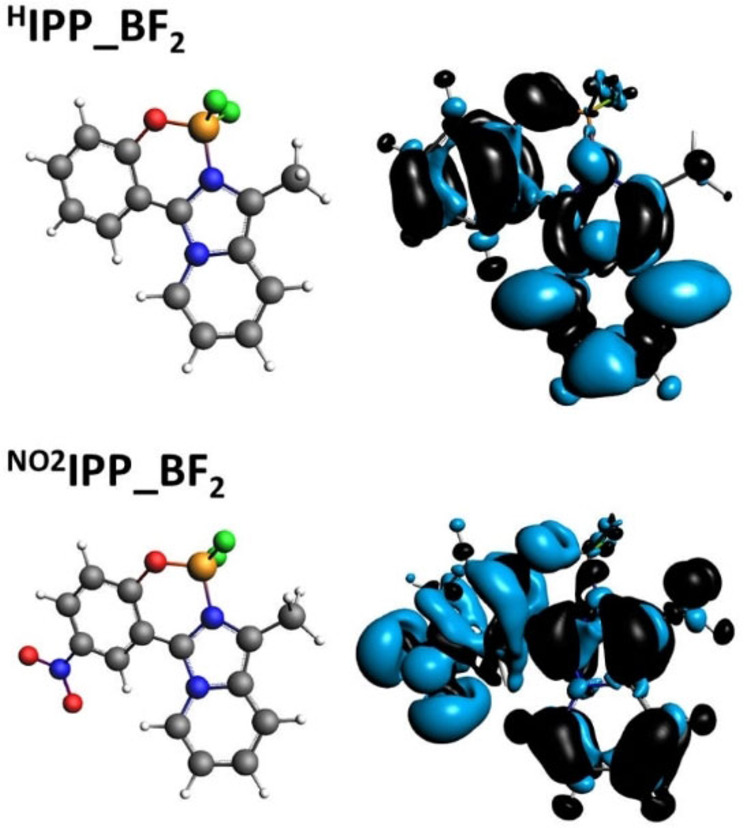
Electron Difference Density Maps (EDDM) for the lowest energy singlet electronic transition computed by TD‐DFT (**black** indicates a decrease in electron density, **blue** indicates an increase).

The Electron Difference Density Map (EDDM) computed at TD‐DFT level are very similar among the series (Figure 5): the electron density mainly moves from the phenolic residue to the imidazo‐pyridine ring for all ^**R**^ **IPP_BF_2_** except for ^**NO2**^ **IPP_BF_2_**, where a more distinct charge separation is observed, with electron density transferred from the nitrogen heterocycle to phenol. Figure 5 reports the EDDM for ^**H**^ **IPP_BF_2_** and ^**NO2**^ **IPP_BF_2_**, whereas EDDM for all ^**R**^ **IPP_BF_2_** compounds are collected in Figure S61. These results support the conclusions based on NTOs shapes, once more underlying the different behavior of ^**NO2**^ **IPP_BF_2_** when compared to the other ^**R**^ **IPP_BF_2_** and explain its distinct emission (i. e., blue for most ^**R**^ **IPP_BF_2_**, orange for ^**NO2**^ **IPP_BF_2_**).

According to the contributions of single orbital transitions reported in Table 4, the higher percentages of contributions are associated to either homo‐lumo or homo‐lumo+1 transitions Table 5 collects the calculated homo, lumo and lumo+1 energies for ^**R**^ **IPP_BF_2_** compounds: as expected, the introduction of an electron donor substituent like Me or OMe enhances the homo energy, whereas halogens lead to the opposite direction. The decrease in homo energy is even more dramatic for the nitro derivative. The lumo energies follow the same trend, increasing with donor substituents and decreasing after introduction of halogens and NO_2_. Worthy of note, the homo‐lumo energy gap is maintained quite constant over the series, ranging from 2.087 (^**OMe**^ **IPP_BF_2_**) to 2.472 eV (^**Cl**^ **IPP_BF_2_**).

**Table 5:** Calculated HOMO, LUMO and LUMO+1 energies (eV) and HOMO‐LUMO energy gap for ^**R**^ **IPP_BF_2_**.

^**R**^ **IPP_BF_2_**	homo	lumo	lumo+1	Δ_H‐L_	σ_p_
^**H**^ **IPP_BF_2_**	−5.339	−2.976	−2.305	2.363	0
^**Me**^ **IPP_BF_2_**	−5.228	−2.940	−2.287	2.288	−0.17
^**OMe**^ **IPP_BF_2_**	−5.048	−2.961	−2.321	2.087	−0.27
^**F**^ **IPP_BF_2_**	−5.402	−3.082	−2.410	2.320	0.06
^**Cl**^ **IPP_BF_2_**	−5.414	−3.098	−2.396	2.316	0.18
^**Br**^ **IPP_BF_2_**	−5.445	−3.114	−2.416	2.330	0.23
^**I**^ **IPP_BF_2_**	−5.434	−3.105	−2.414	2.472	0.23
^**NO2**^ **IPP_BF_2_**	−5.860	−3.541	−3.060	2.319	0.78

**Figure 6:**
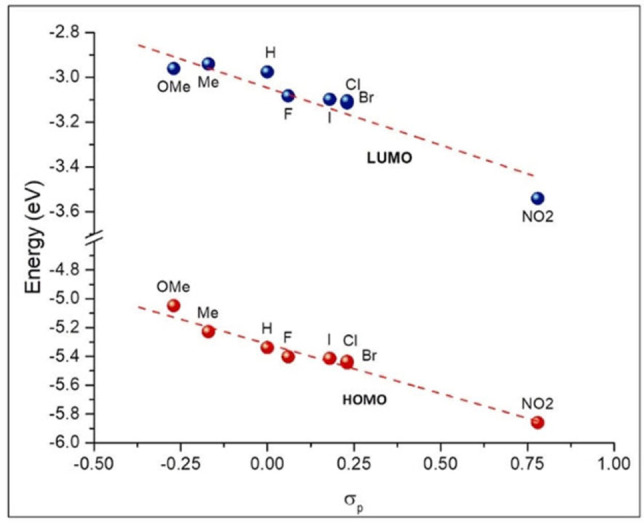
HOMO (red) and LUMO (blue) energies of ^**R**^ **IPP_BF_2_** vs. Hammet's σ_p_ of substituent R. Linear fit: R^2^=0.916 and 0.967 respectively for LUMO and HOMO.

We then decided to search for a possible correlation between the electronic properties of ^**R**^ **IPP_BF_2_** compounds and the calculated homo, lumo and lumo+1 energies, which are in turn related to the emissive properties. As reported in Figure S62 (Supporting Information) lumo+1 energies are related by a linear relationship to lumo energies for all ^**R**^ **IPP_BF_2_** derivatives. Thus, homo‐lumo energy gap can be used in the studied correlations, since it describes also contributions from lumo+1. We chose σ_p_ Hammett constants of the substituent R as the best descriptor of its electronic features. When correlated to σ_p_, homo and lumo energies follow a linear trend, with the energy of both frontier orbitals decreasing in the presence of electron‐withdrawing substituents (namely, with increasing σ_p_). Worthy of note, irrespective of σ_p_ (i. e., the electronic character of R), the homo/lumo energy gap is maintained almost constant over the whole series (Figure 6, Table 5), including ^**NO2**^ **IPP_BF_2_**. Thus, as previously discussed, the origin of its different emission is ascribed to the diverse electronic transitions due to the different topology of NTOs involved.

## Conclusion

In this work we presented the synthesis, the characterization, and the study of the photophysical properties of a series of (imidazo[1,5‐*a*]pyridine‐3‐yl)phenols functionalized with the BF_2_ fragment. The series was obtained by changing substituent R para to the hydroxyl group on the phenolic ring. Nearly all these compounds show a blue fluorescence emission, with large Stokes shifts, good absolute quantum yields as well as excellent photostability in solution. TD‐DFT calculations performed on both S_0_ and S_1_ allowed to explain the different emissive properties (i. e., solvent effect) of the NO_2_‐substituted derivative compared to the other compounds of the series. The main electronic transitions were then defined as ^1^ILT for ^**R**^ **IPP_BF_2_** and ^1^CT for ^**NO2**^ **IPP_BF_2_**. The emission of our compounds has also been correlated to the electronic properties of the substituent R on the phenolic ring, leading to a linear correlation between R Hammett constant (σ_p_) and the homo‐lumo energy gap. This study proves the possibility to use imidazo[1,5‐*a*]pyridine phenols as N,O ligands toward BF_2_ fragment, thus expanding the scope of emissive boron containing compounds.

Due to their good optical properties, especially their high Stoke shifts and photostability, we believe that these compounds constitute an interesting class of blue‐emissive dyes. Furthermore, the possibility to functionalize these molecules by changing the substituent R according to the desired properties (such as solubility in water, or addition of long alkyl chains), while maintaining the intense blue fluorescence, suggests that these compounds can be an interesting alternative for producing cheap blue‐emissive dyes.

## Experimental Section

**General procedure for the synthesis of boron difluoride compounds**^**R**^**IPP_BF_2_**: (imidazo[1,5‐*a*]pyridine‐3‐yl)phenol ^**R**^ **IPP** (1 g, 1 eq) was suspended in 6 mL of deoxygenated dichloromethane, then BF_3_⋅Et_2_O (2.5 equiv.), diluted in 1–2 mL of deoxygenated CH_2_Cl_2_, was added dropwise. Finally, Et_3_N (1.2 equiv.) was added. An exothermic reaction occurred, leading to the formation of a dark red solution, which was stirred at room temperature for a time varying from 30 minutes to 2 h. During this time, precipitation of a solid occurred, which was filtered by suction filtration and washed with a small amount of cold dichloromethane, then dried in vacuo to give a crude solid. This was dissolved in few milliliters of dichloromethane (max 5 mL) and the solution was filtered over a pad of silica gel to remove the last traces of Et_3_NHF. The solvent was then removed under reduced pressure to give the pure product.

Deposition Number(s) 2076375 (^**H**^ **IPP_BF**_**2**_) and 2076376 (^**OMe**^ **IPP_BF**_**2**_) contain(s) the supplementary crystallographic data for this paper. These data are provided free of charge by the joint Cambridge Crystallographic Data Centre and Fachinformationszentrum Karlsruhe Access Structures service www.ccdc.cam.ac.uk/structures.

## Conflict of interest

The authors declare no conflict of interest.

## Supporting information

As a service to our authors and readers, this journal provides supporting information supplied by the authors. Such materials are peer reviewed and may be re‐organized for online delivery, but are not copy‐edited or typeset. Technical support issues arising from supporting information (other than missing files) should be addressed to the authors.

Supporting Information
