# Supplementary material for: Driving the Emission Towards Blue by Controlling the HOMO‐LUMO Energy Gap in BF2‐Functionalized 2‐(Imidazo[1,5‐a]pyridin‐3‐yl)phenols
Source: Chemistry. 2021 Jul 12;27(48):12380–7. doi: 10.1002/chem.202101520 (PMC8456857; doi:10.1002/chem.202101520)
Supplement: Supplementary file 1 — Supporting Information [file CHEM-27-12380-s001.pdf]

Chemistry–A European Journal

Supporting Information

Driving the Emission Towards Blue by Controlling the HOMO-LUMO Energy Gap in BF_2-Functionalized 2-(Imidazo[1,5-*a*]pyridin-3-yl)phenols

Gioele Colombo, G. Attilio Ardizzoia, Julien Furrer, Bruno Therrien, and Stefano Brenna*

Author Contributions

G.C. Investigation:Lead; Writing – original draft:Equal
G.A. Conceptualization:Equal; Funding acquisition:Equal; Investigation:Supporting; Methodology:Supporting; Supervision:Equal; Writing – review & editing:Equal
J.F. Data curation:Equal; Investigation:Supporting; Methodology:Supporting; Writing – review & editing:Equal
B.T. Data curation:Equal; Investigation:Supporting; Methodology:Supporting; Writing – review & editing:Equal
S.B. Conceptualization:Equal; Funding acquisition:Equal; Investigation:Supporting; Methodology:Supporting; Supervision:Equal; Writing – original draft:Equal; Writing – review & editing:Lead

Table of Contents

Experimental section

**# Figure S1**	TGA/DSC analyses of ^{H}**IPP_BF$_2$·xH$_2$O**.
**# Figures S2-S3**	X-ray powder patterns of ^{H}**IPP_BF$_2$** and ^{H}**IPP_BF$_2$·xH$_2$O**.
**# Figures S4-S35**	^{1}H, ^{13}C, ^{19}F and ^{11}B NMR spectra of compounds ^{R}**IPP_BF$_2$**.
**# Figure S36**	^{19}F NMR spectrum of compound ^{H}**IPP_BF$_2$** recorded at -25°C.
**# Figures S37-S45**	Infrared spectra (ATR) of compounds ^{R}**IPP_BF$_2$**.
**# Figure S46**	ORTEP representation of ^{OMe}**IPP_BF$_2$** (molecules A and B).
**# Figure S47**	Normalized UV-vis and emission spectra of ^{H}**IPP_BF$_2$** in various solvents.
**# Figure S48**	Normalized UV-vis and emission spectra of ^{Me}**IPP_BF$_2$** in various solvents.
**# Figure S49**	Normalized UV-vis and emission spectra of ^{Cl}**IPP_BF$_2$** in various solvents.
**# Figure S50**	Normalized UV-vis and emission spectra of ^{NO2}**IPP_BF$_2$** in various solvents.
**# Figure S51**	Normalized UV-vis spectra for compounds ^{R}**IPP_BF$_2$** (CH_2Cl_2, $5 \cdot 10^{-5}$ M).
**# Figure S52**	Normalized excitation and emission spectra for compounds ^{R}**IPP_BF$_2$** (CH_2Cl_2, $5 \cdot 10^{-5}$ M).
**# Figure S53**	CIE 1931 plot for emission of compounds ^{R}**IPP_BF$_2$** (CH_2Cl_2, $5 \cdot 10^{-5}$ M).
**# Figure S54**	Normalized excitation spectra of ^{NO2}**IPP_BF$_2$** in CH_2Cl_2 ($5 \cdot 10^{-5}$ M).
**# Figure S55**	Fitting of the lifetime decay for compounds ^{R}**IPP_BF$_2$** in CH_2Cl_2 ($5 \cdot 10^{-5}$ M).
**# Figure S56**	Multiple emission spectra of ^{H}**IPP_BF$_2$** (CH_2Cl_2, $5 \cdot 10^{-5}$ M).
**# Figure S57**	Intensity of emission of ^{H}**IPP_BF$_2$** *vs*. time (CH_2Cl_2, $5 \cdot 10^{-5}$ M).
**# Figure S58-S59**	Natural Transition Orbitals (NTOs) calculated for compounds ^{R}**IPP_BF$_2$**.
**# Figure S60**	Ground state (S_0) and excited state (S_1) optimized geometries for compounds ^{H}**IPP_BF$_2$** and ^{NO2}**IPP_BF$_2$**.
**# Figure S61**	Electron Difference Density Maps (EDDM) for compounds ^{R}**IPP_BF$_2$**.
**# Figure S62**	Linear correlation between LUMO and LUMO+1 energies for ^{R}**IPP_BF$_2$**.
**# Figures S63-S65**	^{1}H and ^{13}C NMR spectra of ^{Cl}**IPP** and ^{I}**IPP**.
**# Table S1**	Crystallographic and structure refinement parameters for compounds ^{H}**IPP_BF$_2$** and ^{OMe}**IPP_BF$_2$**.
**# Tables S2-S4**	Photophysical data for compounds ^{Me}**IPP_BF$_2$**, ^{Cl}**IPP_BF$_2$** and ^{NO2}**IPP_BF$_2$** recorded in different solvents ($5 \cdot 10^{-5}$ M).

Coordinates of geometry optimization for ground (S_0) and excited state (S_1) for compounds ^{Me}**IPP_BF$_2$**.

Experimental Section

Materials, instrumentation, methods. Infrared Spectra (ATR) were acquired on a Thermo Scientific™ Nicolet™ iS20 FTIR Spectrometer with a 1 cm^{-1} resolution. Elemental analyses were obtained with a Perkin-Elmer CHN Analyzer 2400 Series II. NMR spectra were recorded with an AVANCE III HD Bruker spectrometer operating at 400 MHz for ^{1}H NMR, 100 MHz for $^{13}C\{^{1}H\}$ NMR, 376 MHz for ^{19}F NMR and 128 MHz for ^{11}B NMR. Chemical shifts are given as δ values in ppm relative to residual solvent peaks as the internal reference. *J* values are given in Hz. The UV-vis, excitation and emission spectra were measured using a fluorescence spectrometer (Edinburgh Instruments FS5) equipped with a 150 W continuous Xenon lamp as a light source and were corrected for the wavelength response of the instrument; lifetime measurements were performed on the same FS5 Edinburgh Instruments equipped with an EPLED-320 (Edinburgh Instruments) as the pulsed source. Absolute fluorescence quantum yields in solution were determined using a PhotoMed GmbH K-Sphere Integrating Sphere (3.2 inch. diameter). Analysis of the lifetime decay curve and determination of absolute quantum yields were done using Fluoracle® Software package (Ver. 1.9.1) which runs the FS5 instrument. Aldehydes **1-4**, **6** and **8** have been purchased (TCI Chemicals, Fluorochem) and used without further purifications. Aldehydes **5** and **7** were prepared by direct halogenation of salicylaldehyde, using a slightly modified procedure as reported by Bovosombat.[1]

Synthesis of 2-hydroxy-5-halobenzaldehyde 5 and 7

Salicylaldehyde (2.57 mL, 3 g, 24.57 mmol), *N*-halosuccinimide (24.57 mmol) and *p*-toluensulfonic acid (4.23 g, 24.57 mmol) were dissolved in 8 mL of deoxygenated acetonitrile. The resulting orange solution was left stirring under inert atmosphere overnight. During this time, precipitation of a white solid occurred. The suspension was cooled to 0 °C for 6 hours to favor the precipitation. Then, the solid was filtered, washed with cold acetonitrile, and dried in vacuo. Yield: *5-chloro-2-hydroxybenzaldehyde (**5**)*: 2.58 g, (69.8%). *2-hydroxy-5-iodobenzaldehyde (7)*: 4.15 g, (76.0%). The purity of the products was confirmed by comparison with literature data.[2]

Synthesis of 4-halo-2-(1-methylimidazo[1,5-a]pyridin-3-yl)phenol (^{Cl}IPP and ^{I}IPP)

2-acetylpyridine (2 mL, 2.16 g, 17.83 mmol), ammonium acetate (6.88 g, 89.26 mmol) and 2-hydroxy-5-halobenzaldehyde **5** or **7** (35.66 mmol) were dissolved in 40 mL of deoxygenated glacial acetic acid. The resulting yellow-orange suspension was left stirring under inert atmosphere for 1 week, at room temperature. During this time, precipitation of a solid occurred, and the suspension turned to deep red. The solid was filtered, washed with acetic acid, and discarded. The filtrate was diluted with water and extracted with dichloromethane (4 x 100 mL), the organic phase was washed with an aqueous saturated solution of $NaHCO_3$ and dried over Na_2SO_4. The solvent was removed under reduced pressure to afford a yellow-orange oil that was triturated with hexane. The solid obtained was filtered, washed with hexane, and dried in vacuo.

^{Cl}IPP: Yield: 2.68 g (49.0%). Anal. Calcd (%) for $C_{14}H_{11}ClN_2O$: C, 65.00; H, 4.29; N, 10.83. Found (%): C, 65.15; H, 4.21; N, 10.47. ^{1}H NMR (400 MHz, $CDCl_3$, 298 K, *J* [Hz]): δ = 8.38 (d, *J* = 7.2, 1H), 7.68 (d, *J* = 2.3, 1H), 7.46 (d, *J* = 9.0, 1H), 7.21 (dd, *J* = 8.7, 2.3, 1H), 7.07 (d, *J* = 8.7, 1H), 6.75 (m, 1H), 6.68 (t, *J* = 6.6, 1H), 2.55 (s, 3H). ^{13}C NMR (100 MHz, $CDCl_3$, 298 K): δ = 155.0, 133.0, 128.6, 127.6, 127.3, 123.5, 122.7, 121.7, 118.8, 118.5, 118.1, 115.4, 114.3, 114.1, 12.2. (Figure S63-S-64).

[1] P. Bovonsombat, J. Leykajarakul, C. Khan, K. Pla-on, M. Krause, P. Khanthapura, R. Ali, N. Doowa, *Tetrahedron Lett.* **2009**, *50*, 2664-2667.

[2] a) Y. Niu, R. Wang, P. Shao, Y. Wang, Y. Zhang, *Chem. Eur. J.* **2018**, *24*, 16670–16676; b) C. Huang, K. Zhu, Y. Zhang, Z. Shao, D. Wang, L. Mi, H. Hou, *Inorg. Chem.* **2019**, *58*, 12933−12942.

^{I}IPP: Yield: 4.62 g (65.1%). Anal. Calcd (%) for $C_{14}H_{11}IN_2O$: C, 48.02; H, 3.17; N, 8.00. Found (%): C, 48.22; H, 3.16; N, 7.62. ^{1}H NMR (400 MHz, $CDCl_3$, 298 K, *J* [Hz]): δ = 8.34 (d, *J* = 7.2, 1H), 7.97 (d, *J* = 1.7, 1H), 7.51 (dd, *J* = 8.6, 1.7, 1H), 7.44 (d, *J* = 9.0, 1H), 6.91 (d, *J* = 8.6, 1H), 6.74 (m, 1H), 6.78 (t, *J* = 6.6, 1H), 2.54 (s, 3H). ^{13}C NMR (100 MHz, $CDCl_3$, 298 K): δ = 153.1, 137.6, 131.7, 128.2, 127.1, 122.7, 121.5, 120.0, 119.7, 118.3, 118.1, 116.1, 114.8, 114.1, 88.5, 12.1. (Figure S65-S66).

General procedure for the synthesis of boron difluoride compounds ^{R}IPP_BF$_2$

(imidazo[1,5-*a*]pyridine-3-yl)phenol **^{R}IPP** (1 g, 1 eq) was suspended in 6 mL of deoxygenated dichloromethane, then $BF_3{\cdot}Et_2O$ (2.5 eq), diluted in 1-2 mL of deoxygenated CH_2Cl_2, was added dropwise. Finally, Et_3N (1.2 eq) was added. An exothermic reaction occurred, leading to the formation of a dark red solution, which was stirred at room temperature for a time varying from 30 minutes to 2 hours. During this time, precipitation of a solid occurred, which was filtered by suction filtration and washed with a small amount of cold dichloromethane, then dried in vacuo to give a crude solid. This was dissolved in few milliliters of dichloromethane (max 5 mL) and the solution was filtered over a pad of silica gel to remove the last traces of Et_3NHF. The solvent was then removed under reduced pressure to give the pure product.

^{H}IPP_BF$_2$: The compound was obtained as the partially hydrated species **^{H}IPP_BF$_2$·*x*H$_2$O**. Then, the anhydrous species **^{H}IPP_BF$_2$** was obtained by dissolving as synthesized **^{H}IPP_BF$_2$·*x*H$_2$O** (0.1 g) in dichloromethane (25 mL), so that the final solution had a concentration of about 10^{-5} M. The pink-orange solution was left stirring at room temperature until it became light yellow. Then the solvent was removed under reduced pressure, obtaining a yellow solid in quantitative yield. Anal. Calcd (%) for $C_{14}H_{11}BF_2N_2O$: C, 61.81; H, 4.08; N, 10.30. Found (%): C, 61.55; H, 4.01; N, 10.18. ^{1}H NMR (400 MHz, CD_2Cl_2, 278 K, *J* [Hz]): δ = 8.52 – 8.41 (m, 1H), 7.82 (dd, *J* = 8.1, 1.5, 1H), 7.56 – 7.47 (m, 1H), 7.37-7.32 (m, 1H), 7.12 (dd, *J* = 8.3, 1.2, 1H), 7.03-6.98 (m, 1H), 6.94 – 6.80 (m, 2H), 2.62 (s, 3H). ^{13}C NMR (100 MHz, CD_2Cl_2, 298 K): δ = 154.92, 122.35, 122.18, 120.67, 120.10, 119.75, 119.01, 117.19, 9.34. ^{19}F NMR (376 MHz, CD_2Cl_2, 298 K, *J* [Hz]): δ = -140.01 (q, *J* = 13.6). ^{11}B NMR (128 MHz, CD_2Cl_2, 298 K, *J* [Hz]): δ = 1.15 (t, *J* = 14.9). FT-IR (ATR): (cm^{-1}): ν = 1033 (s), 1061 (s) 1093 (s). Single crystals suitable for X-ray analysis were obtained by slow diffusion of hexane in dichloromethane.

^{Me}IPP_BF$_2$: Yield: 0.76 g (59.3%). Anal. Calcd (%) for $C_{15}H_{13}BF_2N_2O$: C, 62.98; H, 4.58; N, 9.79. Found (%): C, 62.79; H, 4.62; N, 9.73. ^{1}H NMR (400 MHz, CD_2Cl_2, 298 K, *J* [Hz]): δ = 8.84 – 8.43 (m, 1H), 7.86 – 7.67 (m, 1H), 7.67 – 7.49 (m, 1H), 7.38 – 7.23 (m, 1H), 7.13 (d, *J* = 8.4, 1H), 7.05 – 6.88 (m, 2H), 2.73 (s, 3H), 2.44 (s, 3H). ^{13}C NMR (100 MHz, CD_2Cl_2, 298 K): δ = 152.81, 132.74, 129.18, 127.55, 122.47, 122.14, 120.57, 119.79, 118.96, 117.04, 110.53, 20.61, 9.34. ^{19}F NMR (376 MHz, CD_2Cl_2, 298 K, *J* [Hz]): δ = -140.28 (q, *J* = 13.6). ^{11}B NMR (128 MHz, CD_2Cl_2, 298 K, *J* [Hz]): δ = 1.18 (t, *J* = 15.3). FT-IR (ATR): (cm^{-1}): ν = 1036 (s), 1066 (s) 1090 (s).

^{OMe}IPP_BF$_2$: Yield: 0.69 g (58.0%). Anal. Calcd (%) for $C_{15}H_{13}BF_2N_2O_2$: C, 59.64; H, 4.42; N, 9.27. Found (%): C, 59.38; H, 4.31; N, 9.20. ^{1}H NMR (400 MHz, CD_2Cl_2, 298 K, *J* [Hz]): δ = 8.62 – 8.52 (m, 1H), 7.68 – 7.59 (m, 1H), 7.43 (d, *J* = 2.8, 1H), 7.18 (d, *J* = 9.0, 1H), 7.07 (dd, *J* = 9.0, 2.9, 1H), 7.05 – 6.95 (m, 2H), 3.90 (s, 3H), 2.74 (s, 3H). ^{13}C NMR (100 MHz, CD_2Cl_2, 298 K): δ = 152.76, 149.05, 127.71, 122.72, 122.29, 120.71, 120.61, 119.04, 117.53, 117.25, 110.82, 107.63, 56.06, 9.36. ^{19}F NMR (376 MHz, CD_2Cl_2, 298 K, *J* [Hz]): δ = -140.71 (q, *J* = 13.1).^{11}B NMR (128 MHz, CD_2Cl_2, 298 K, *J* [Hz]): δ = 1.15 (t, *J* = 14.8). FT-IR (ATR): (cm^{-1}): ν = 1032 (s), 1055 (s) 1084 (s). Single crystals suitable for X-ray analysis were obtained by slow diffusion of hexane in dichloromethane.

^{F}IPP_BF$_2$: Yield: 0.74 g (61.7%). Anal. Calcd (%) for $C_{14}H_{10}BF_3N_2$: C, 57.97; H, 3.48; N, 9.66. Found (%): C, 57.61; H, 3.37; N, 9.48. ^{1}H NMR (400 MHz, CD_2Cl_2, 298 K, *J* [Hz]): δ = 8.58 – 8.47 (m, 1H), 7.82 – 7.54 (m, 2H), 7.25 – 7.17 (m,

2H), 7.14 – 7.00 (m, 2H), 2.76 (s, 3H). ^{13}C NMR (100 MHz, CD_2Cl_2, 298 K): δ = 157.02, 154.66, 128.00, 122.05, 121.06, 119.15, 118.68, 118.45, 117.68, 108.40, 108.14, 9.36. ^{19}F NMR (376 MHz, CD_2Cl_2, 298 K, *J* [Hz]): δ = -123.56, -140.17 (q, *J* = 13.1). ^{11}B NMR (128 MHz, CD_2Cl_2, 298 K, *J* [Hz]): δ = 1.17 (t, *J* = 14.4). FT-IR (ATR): (cm^{-1}): ν = 1043 (s), 1066 (s) 1106 (s).

^{Cl}IPP_BF$_2$: Yield: 0.65 g (61.8%). Anal. Calcd (%) for $C_{14}H_{10}BF_2ClN_2O$: C, 53.43; H, 3.20; N, 8.90. Found (%): C, 53.46; H, 3.40; N, 9.07. ^{1}H NMR (400 MHz, $CDCl_3$, 298 K, *J* [Hz]): δ = 8.68 – 8.32 (m, 1H), 7.78 (m, 1H), 7.66 – 7.54 (m, 1H), 7.34 (dd, J = 8.9, 2.4, 1H), 7.18 (d, J = 8.9, 1H), 7.06 – 6.92 (m, 2H), 2.74 (s, 3H). ^{13}C NMR (100 MHz, CD_2Cl_2, 298 K): δ = 153.66, 131.70, 129.73, 127.83, 124.61, 123.41, 122.14, 121.93, 121.37, 121.05, 119.29, 117.71, 111.72, 9.64. ^{19}F NMR (376 MHz, CD_2Cl_2, 298 K, *J* [Hz]): δ = -139.80 (q, *J* = 11.4). ^{11}B NMR (128 MHz, CD_2Cl_2, 298 K, *J* [Hz]): δ = 1.14 (t, *J* = 14.2). FT-IR (ATR): (cm^{-1}): ν = 1033 (s), 1085 (s) 1103 (s).

^{Br}IPP_BF$_2$: Yield: 0.84 g (72.5%). Anal. Calcd (%) for $C_{14}H_{10}BF_2N_2OBr$: C, 47.91; H, 2.87; N, 7.98. Found (%): C, 47.75; H, 2.96; N, 7.63. ^{1}H NMR (400 MHz, CD_2Cl_2, 298 K, *J* [Hz]): δ = 8.54 – 8.37 (m, 1H), 7.92 (d, *J* = 2.3, 1H), 7.67 – 7.49 (m, 1H), 7.43 (dd, *J* = 8.8, 2.3, 1H), 7.03 (d, *J* = 8.8, 1H), 6.99 – 6.86 (m, 2H), 2.63 (s, 3H). ^{13}C NMR (100 MHz, CD_2Cl_2, 298 K): δ = 153.95, 134.37, 128.05, 124.49, 123.15, 122.22, 121.90, 121.14, 119.13, 117.76, 111.51, 9.36. ^{19}F NMR (376 MHz, CD_2Cl_2, 298 K, *J* [Hz]): δ = -139.89 (q, *J* = 12.7). ^{11}B NMR (128 MHz, CD_2Cl_2, 298 K, *J* [Hz]): δ = 1.03 (t, *J* = 14.4). FT-IR (ATR): (cm^{-1}): ν = 1032 (s), 1069 (s) 1094 (s).

^{I}IPP_BF$_2$: Yield: 0.63 g (55.4%). Anal. Calcd (%) for $C_{14}H_{10}BF_2IN_2O$: C, 42.25; H, 2.53; N, 7.04. Found (%): C, 41.95; H, 2.68; N, 7.00. ^{1}H NMR (400 MHz, CD_2Cl_2, 298 K, *J* [Hz]): δ = 8.49 – 8.41 (m, 1H), 8.07 (d, *J* = 2.1, 1H), 7.69 – 7.54 (m, 2H), 7.09 – 6.92 (m, 3H), 2.73 (s, 3H). ^{13}C NMR (100 MHz, CD_2Cl_2, 298 K): δ = 154.71, 140.39, 130.12, 129.29, 127.82, 123.36, 122.78, 122.15, 120.96, 119.26, 117.74, 113.25, 80.89, 9.66. ^{19}F NMR (376 MHz, CD_2Cl_2, 298 K, *J* [Hz]): δ = 139.74 (q, *J* = 11.4). ^{11}B NMR (128 MHz, CD_2Cl_2, 298 K, *J* [Hz]): δ = 1.08 (t, *J* = 14.3). FT-IR (ATR): (cm^{-1}): ν = 1036 (s), 1056 (s) 1094 (m).

^{NO2}IPP_BF$_2$: Yield: 0.87 g (73.6%). Anal. Calcd (%) for $C_{14}H_{10}BF_2N_3O_2$: C, 53.04; H, 3.18; N, 13.25. Found (%): C, 53.03; H, 3.43; N, 12.91. ^{1}H (400 MHz, CD_2Cl_2, 298 K, *J* [Hz]): δ = 8.91 (d, *J* = 2.4, 1H), 8.70 – 8.67 (m, 1H), 7.86 – 7.67 (m, 1H), 7.34 (dd, *J* = 9.1, 1.3, 1H), 7.26 – 7.08 (m, 2H), 2.79 (s, 3H). ^{13}C NMR (100 MHz, CD_2Cl_2, 298 K): δ = 159.91, 140.25, 128.54, 122.16, 121.75, 121.15, 120.56, 119.26, 118.56, 118.40, 115.13, 114.98, 9.37. ^{19}F NMR (376 MHz, CD_2Cl_2, 298 K, *J* [Hz]): δ = -138.77 (q, *J* = 12.4). ^{11}B NMR (128 MHz, CD_2Cl_2, 298 K, *J* [Hz]): δ = 1.05 (t, *J* = 13.4). FT-IR (ATR): (cm^{-1}): ν = 1061 (s) 1093 (s).

X-ray crystallography

A crystal of **^{H}IPP_BF$_2$** and **^{OMe}IPP_BF$_2$** was mounted on a Stoe Image Plate Diffraction system equipped with a ϕ circle goniometer, using Mo-Kα graphite monochromated radiation (λ = 0.71073 Å) with ϕ range 0–200º. The structure was solved by direct methods using the program SHELXS,[3] while refinement and all further calculations were carried out using SHELXL.[4] The H-atoms were included in calculated positions and treated as riding atoms using the SHELXL default parameters. The non-H atoms were refined anisotropically, using weighted full-matrix least-square on F^2. Crystallographic details are summarized in Table S1. Figure 1 was drawn with ORTEP–32.[5]

CCDC-2076375 (**^{H}IPP_BF$_2$**) and 2076376 (**^{OMe}IPP_BF$_2$**) contain the supplementary crystallographic data for this paper. These data are provided free of charge by the joint Cambridge Crystallographic Data Centre and Fachinformationszentrum Karlsruhe Access Structures service https://www.ccdc.cam.ac.uk/structures

Computational details

All calculations were carried out at the density functional level of theory (DFT) with the ADF2020.102 program package.[6] The PBE functional plus a D3 dispersion correction energy term (PBE-D3)[7] was employed for all calculations. Frequency analyses were performed for all optimized structures to establish the nature of the stationary points. TD-DFT implemented in the ADF package was used to determine the excitation energies: the 40 lowest singlet-singlet excitations were calculated by using the optimized geometries. For geometry optimizations all atoms but iodine were described through TZ2P basis sets [triple-ξ Slater-type orbitals (STOs) plus two polarization function]. For iodine atom the QZ4P basis set (core triple-ξ, valence quadruple-ξ, and four sets of polarization functions) was applied and relativistic effects were included using the scalar relativistic *zeroth-order regular approximation* (ZORA) formalism.[8] The corresponding augmented basis set was employed in TD-DFT calculations.[9] Restricted formalism, no-frozen-core approximation (all-electron) and no-symmetry constrains were used in all calculations. Solvent effects were simulated employing the conductor-like continuum solvent model (COSMO)[10] as implemented in the ADF suite.

---

[3] G. M. Sheldrick, *Acta Cryst.* **1990,** *A46*, 467-473.
[4] G. M. Sheldrick, *Acta Cryst.* **2015**, *C71*, 3-8.
[5] L. J. Farrugia, *J. Appl. Cryst.* **1997**, *30*, 565
[6] a) G. te Velde, F. M. Bickelhaupt, E. J. Baerends, C. Fonseca Guerra, S. J. A. van Gisbergen, J. G. Snijders, T. Ziegler, *J. Comp. Chem.* **2001**, *22*, 931-967; b) C. Fonseca Guerra, J. G. Snijders, G. te Velde, E. J. Baerends, *Theor. Chem. Acc.* **1998**, *99*, 391-403; c) E. J. Baerends, T. Ziegler, J. Autschbach, D. Bashford, A. Bérces, F. M. Bickelhaupt, C. Bo, P. M. Boerrigter, L. Cavallo, D. P. Chong, L. Deng, R. M. Dickson, D. E. Ellis, M. van Faassen, L. Fan, T. H. Fischer, C. Fonseca Guerra, M. Franchini, A. Ghysels, A. Giammona, S. J .A. van Gisbergen, A. W. Götz, J. A. Groeneveld, O. V. Gritsenko, M. Grüning, S. Gusarov, F. E. Harris, P. van den Hoek, C. R. Jacob, H. Jacobsen, L. Jensen, J. W. Kaminski, G. van Kessel, F. Kootstra, A. Kovalenko, M. V. Krykunov, E. van Lenthe, D. A. McCormack, A. Michalak, M. Mitoraj, S. M. Morton, J. Neugebauer, V. P. Nicu, L. Noodleman, V. P. Osinga, S. Patchkovskii, M. Pavanello, P. H. T. Philipsen, D. Post, C. C. Pye, W. Ravenek, J. I. Rodríguez, P. Ros, P. R. T. Schipper, H. van Schoot, G. Schreckenbach, J. S. Seldenthuis, M. Seth, J. G. Snijders, M. Solà, M. Swart, D. Swerhone, G. te Velde, P. Vernooijs, L. Versluis, L. Visscher, O. Visser, F. Wang, T. A. Wesolowski, E. M. van Wezenbeek, G. Wiesenekker, S. K. Wolff, T. K. Woo, A. L. Yakovlev, ADF2014, SCM, Theoretical Chemistry, Vrije Universiteit, Amsterdam, The Netherlands, http://www.scm.com
[7] S. Grimme, J. Antony, S. Ehrlich, S. Krieg, *J. Chem. Phys.* **2010**, *132*, 154104.
[8] a) E. van Lenthe, E. J. Baerends, J. G. Snijders, *J. Chem. Phys.* **1993,** *99*, 4597-5600; b) E. van Lenthe, E. J. Baerends, J. G. Snijders, *J. Chem. Phys.* **1994,** *101*, 9783-9792; c) E. van Lenthe, A. E. Ehlers, E. J. Baerends, *J. Chem. Phys.* **1999,** *110*, 8943-8953; d) E. van Lenthe, J. G. Snijders, E. J. Baerends, *J. Chem. Phys.* **1996,** *105*, 6505-6516; e) E. van Lenthe, R. van Leeuwen, E. J. Baerends, J. G. Snijders, *Int. J. Quantum Chem.* **1996** *57*, 281-293.
[9] D. P. Chong, *Mol. Phys.* **2005**, *103*, 749-761.
[10] a) A. Klamt, G. J. Schürmann, *J. Chem. Soc. Perkin Trans. 2.* **1993**, 799-805; b) A. Klamt, V. Jonas, *J. Chem. Phys.* **1996**, *105*, 9972-9981; c) C. C. Pye, T. Ziegler, *Theor. Chem. Acc.* **1999**, *101*, 396-408.

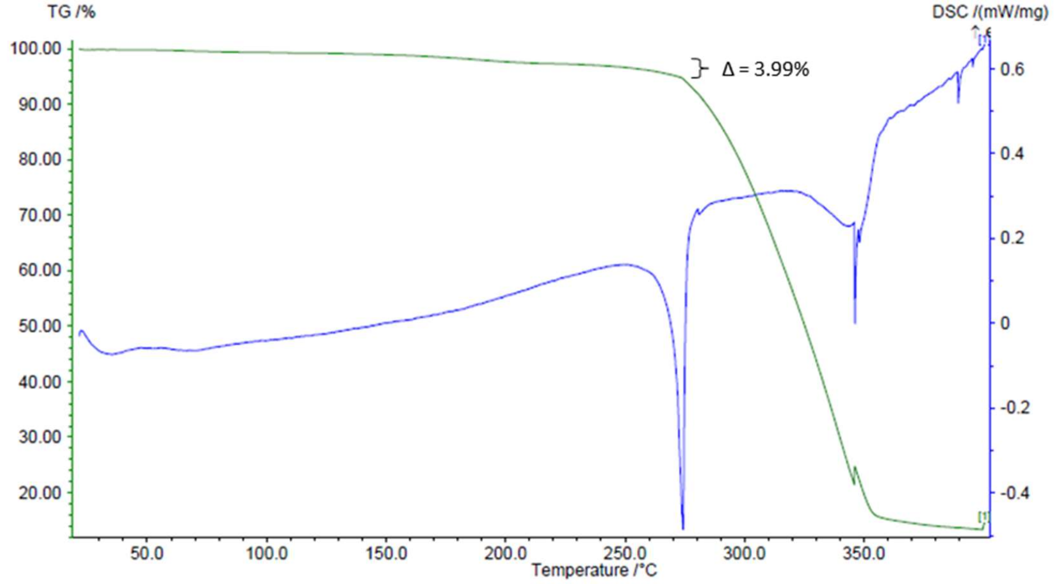

Figure S1. TGA/DSC analyses conducted on one batch of **^{H}IPP_BF$_2$·*x*H$_2$O**. A loss of 3.99% weight is observed starting from T = 100°C, suggesting water loss.

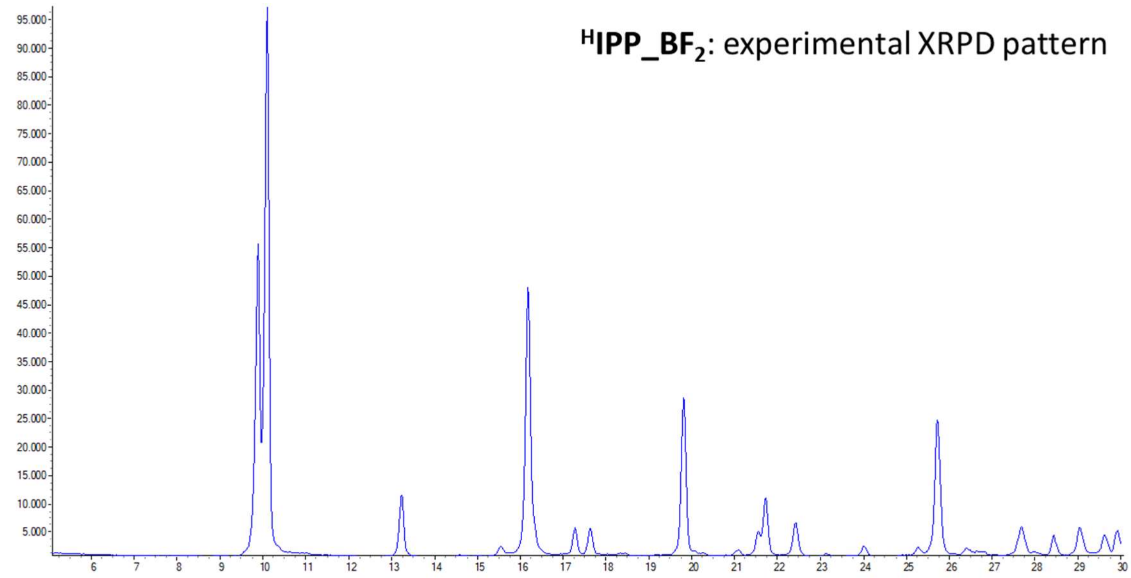

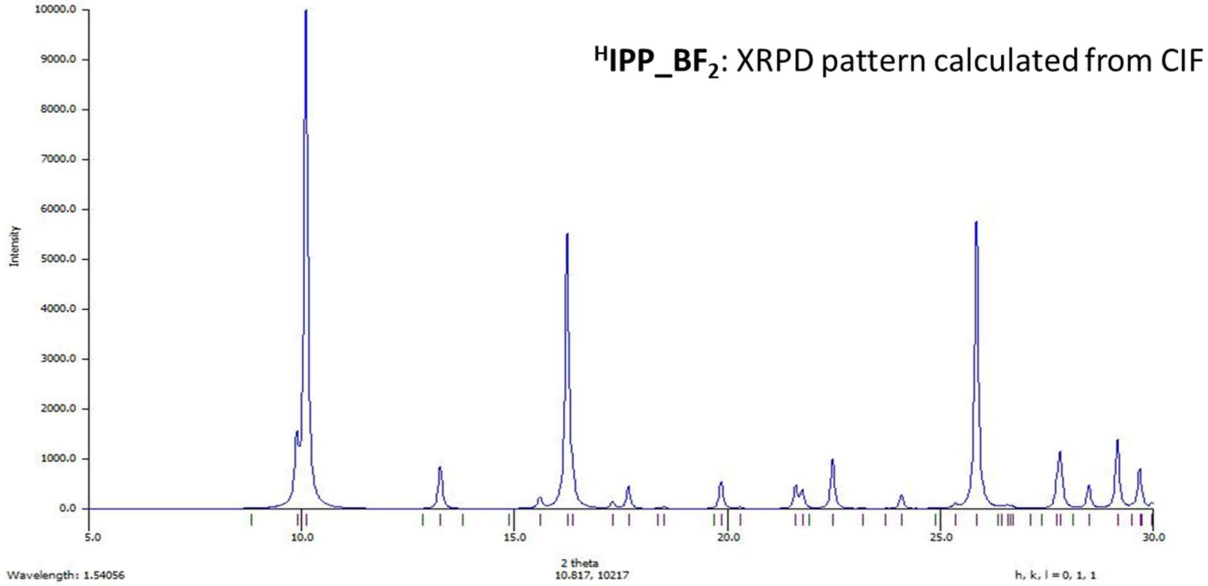

Figure S2. Experimental (up) and calculated (bottom) X-ray powder pattern of **^{H}IPP_BF$_2$**.

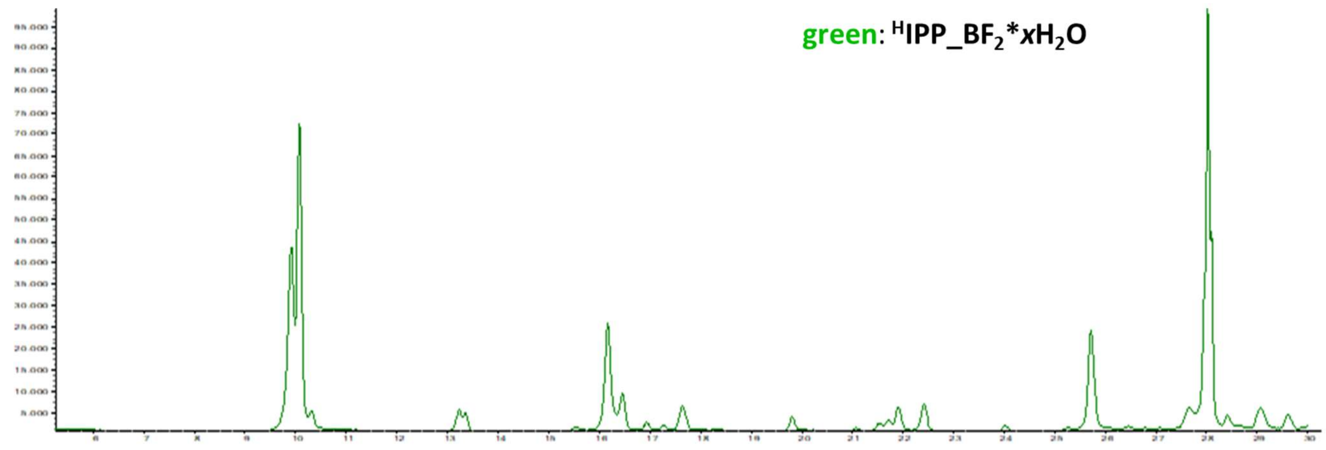

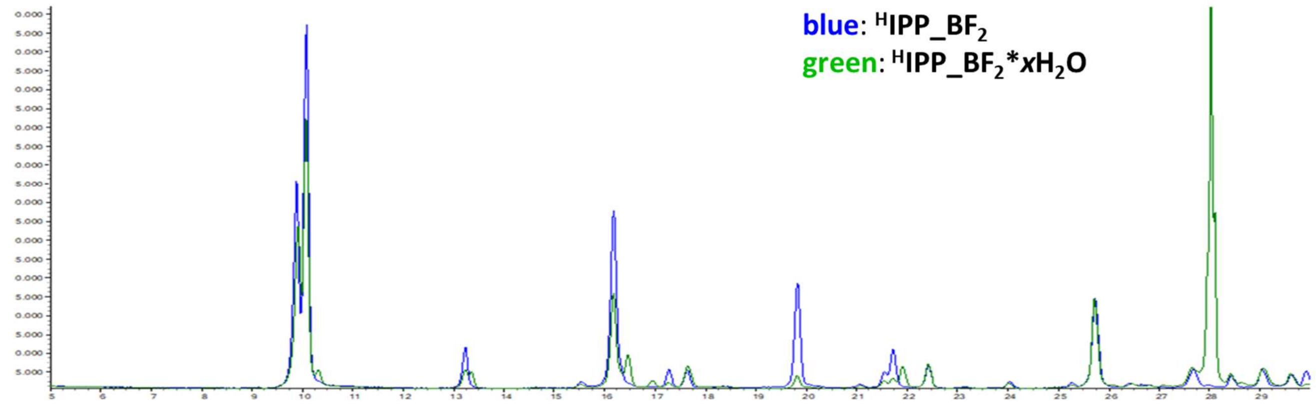

Figure S3. Up: experimental X-ray powder pattern of **^{H}IPP_BF$_2$*xH$_2$O**. Bottom: comparison between experimental X-ray powder pattern of **^{H}IPP_BF$_2$** and **^{H}IPP_BF$_2$*xH$_2$O**.

^{H}IPP_BF$_2$

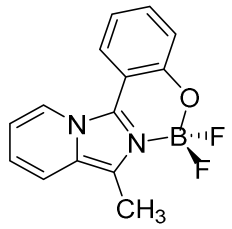

^{1}H NMR (400 MHz, CD_2Cl_2, 278 K, J [Hz]): δ = 8.52 – 8.41 (m, 1H), 7.82 (dd, J = 8.1, 1.5, 1H), 7.56 – 7.47 (m, 1H), 7.37-7.32 (m, 1H), 7.12 (dd, J = 8.3, 1.2, 1H), 7.03-6.98 (m, 1H), 6.94 – 6.80 (m, 2H), 2.62 (s, 3H). ^{13}C NMR (100 MHz, CD_2Cl_2, 298 K): δ = 154.92, 122.35, 122.18, 120.67, 120.10, 119.75, 119.01, 117.19, 9.34. ^{19}F NMR (376 MHz, CD_2Cl_2, 298 K, J [Hz]): δ = -140.01 (q, J = 13.6). ^{11}B NMR (128 MHz, CD_2Cl_2, 298 K, J [Hz]): δ = 1.15 (t, J = 14.9).

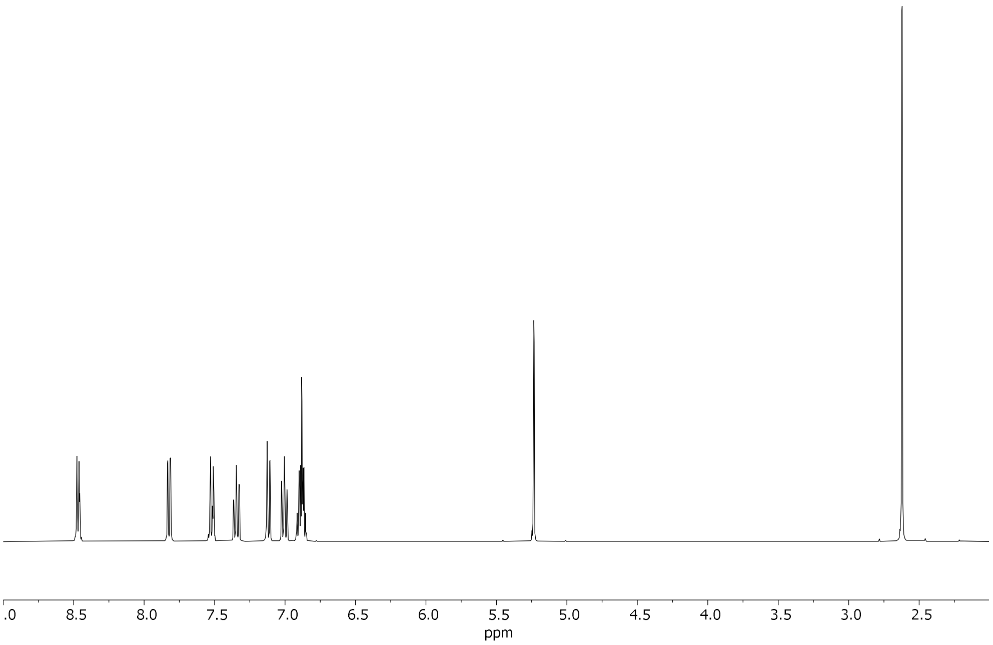

Figure S4. ^{1}H NMR (CD_2Cl_2, 25°C) of **^{H}IPP_BF$_2$**.

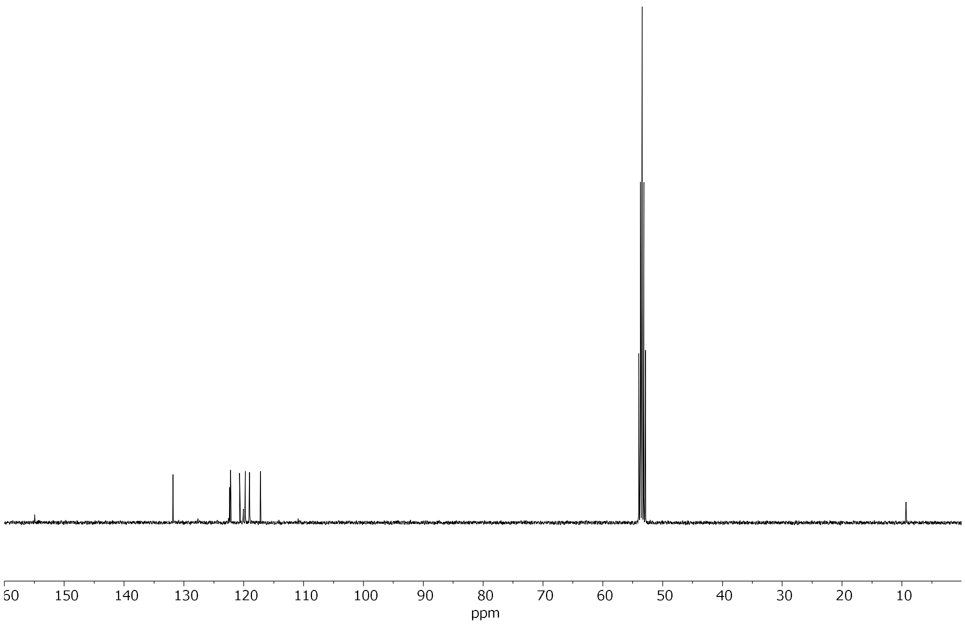

Figure S5. ^{13}C NMR (CD_2Cl_2, 25°C) of **^{H}IPP_BF$_2$**.

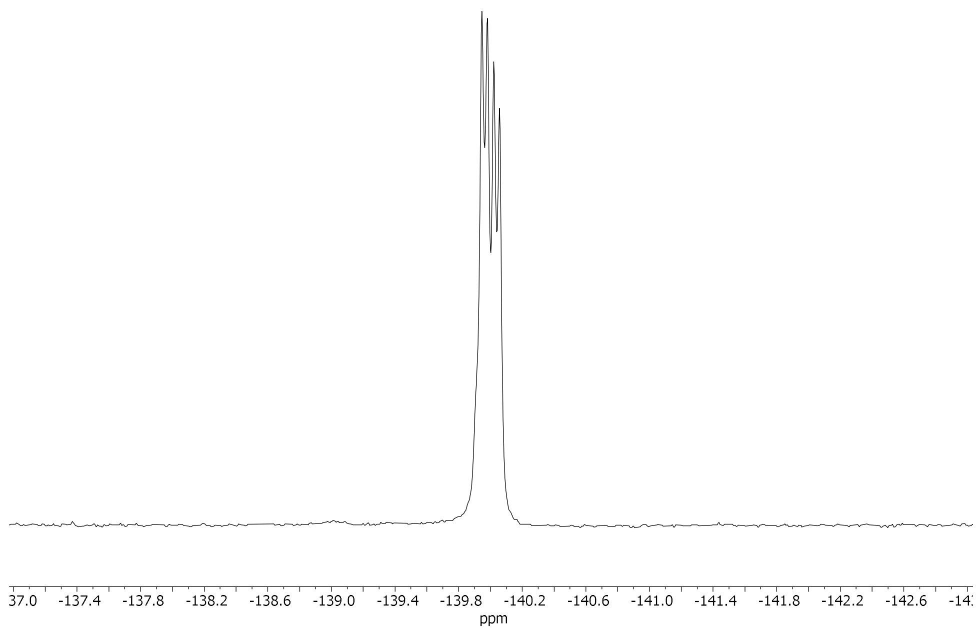

Figure S6. ^{19}F NMR (CD_2Cl_2, 25°C) of **^{H}IPP_BF$_2$**.

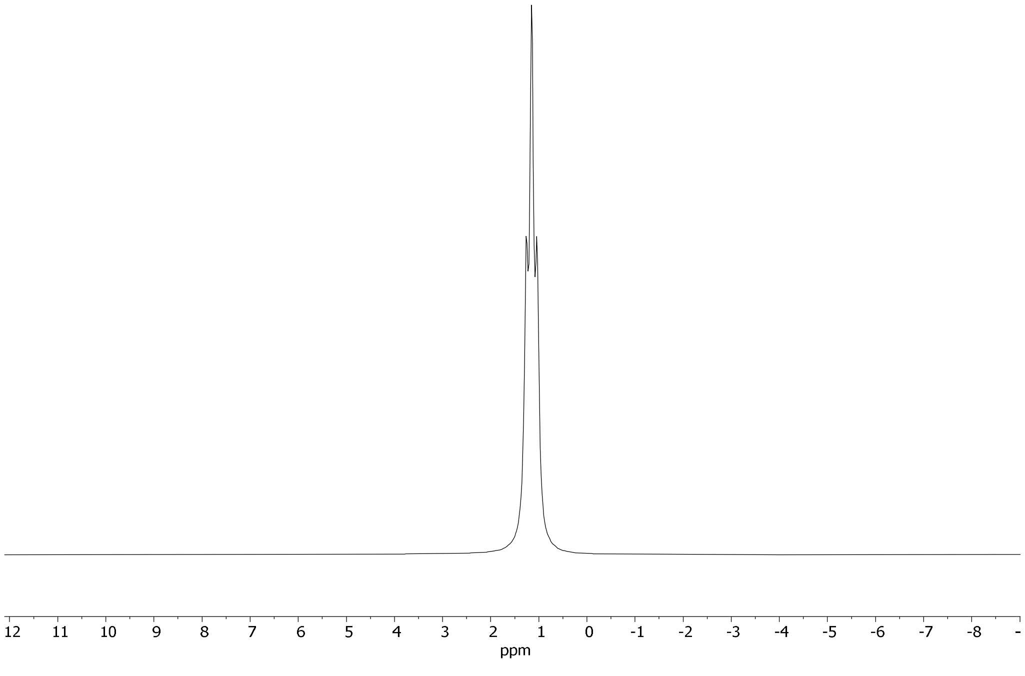

Figure S7. ^{11}B NMR (CD_2Cl_2, 25°C) of **^{H}IPP_BF$_2$**.

^{Me}IPP_BF$_2$

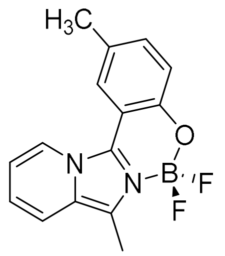

^{1}H NMR (400 MHz, CD_2Cl_2, 298 K, *J* [Hz]): δ = 8.84 – 8.43 (m, 1H), 7.86 – 7.67 (m, 1H), 7.67 – 7.49 (m, 1H), 7.38 – 7.23 (m, 1H), 7.13 (d, *J* = 8.4, 1H), 7.05 – 6.88 (m, 2H), 2.73 (s, 3H), 2.44 (s, 3H). ^{13}C NMR (100 MHz, CD_2Cl_2, 298 K): δ = 152.81, 132.74, 129.18, 127.55, 122.47, 122.14, 120.57, 119.79, 118.96, 117.04, 110.53, 20.61, 9.34. ^{19}F NMR (376 MHz, CD_2Cl_2, 298 K, *J* [Hz]): δ = -140.28 (q, *J* = 13.6). ^{11}B NMR (128 MHz, CD_2Cl_2, 298 K, *J* [Hz]): δ = 1.18 (t, *J* = 15.3).

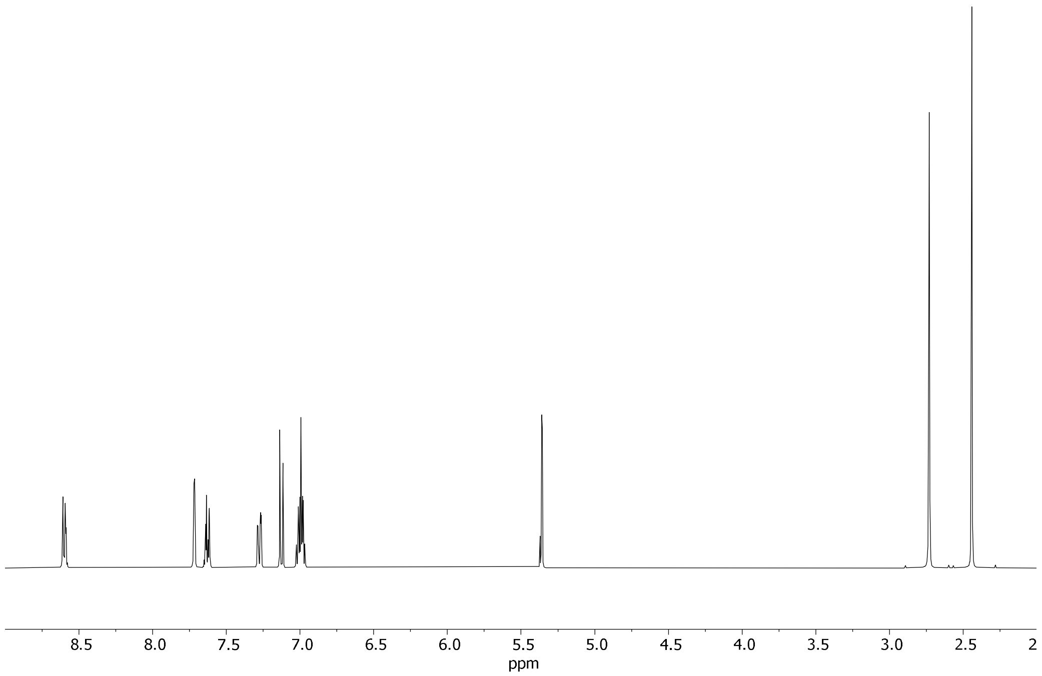

Figure S8. ^{1}H NMR (CD_2Cl_2, 25°C) of **^{Me}IPP_BF$_2$**.

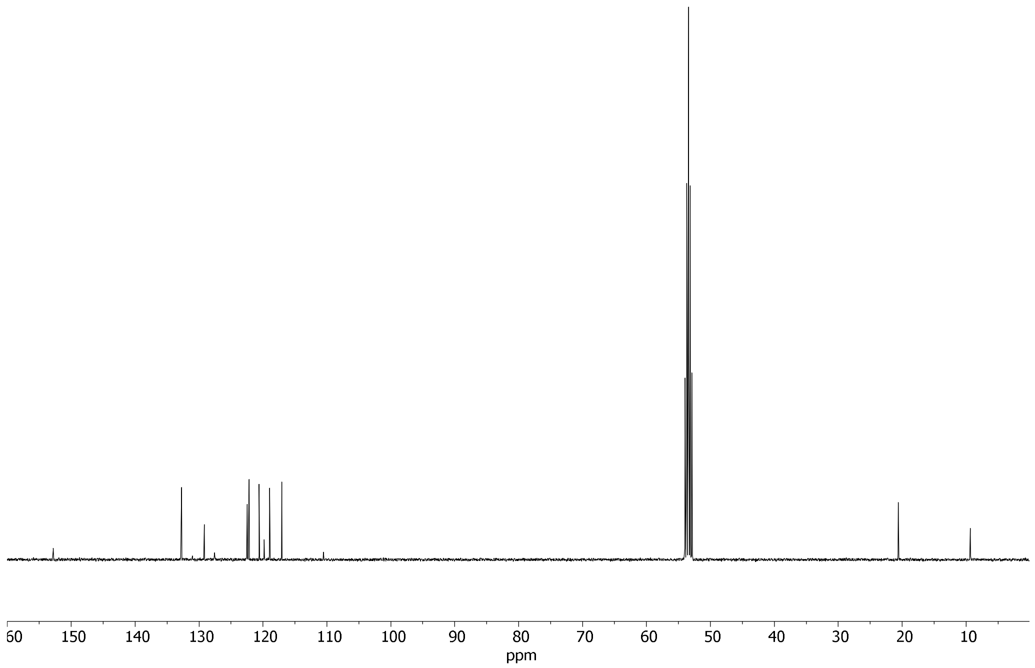

Figure S9. ^{13}C NMR (CD_2Cl_2, 25°C) of **^{Me}IPP_BF$_2$**.

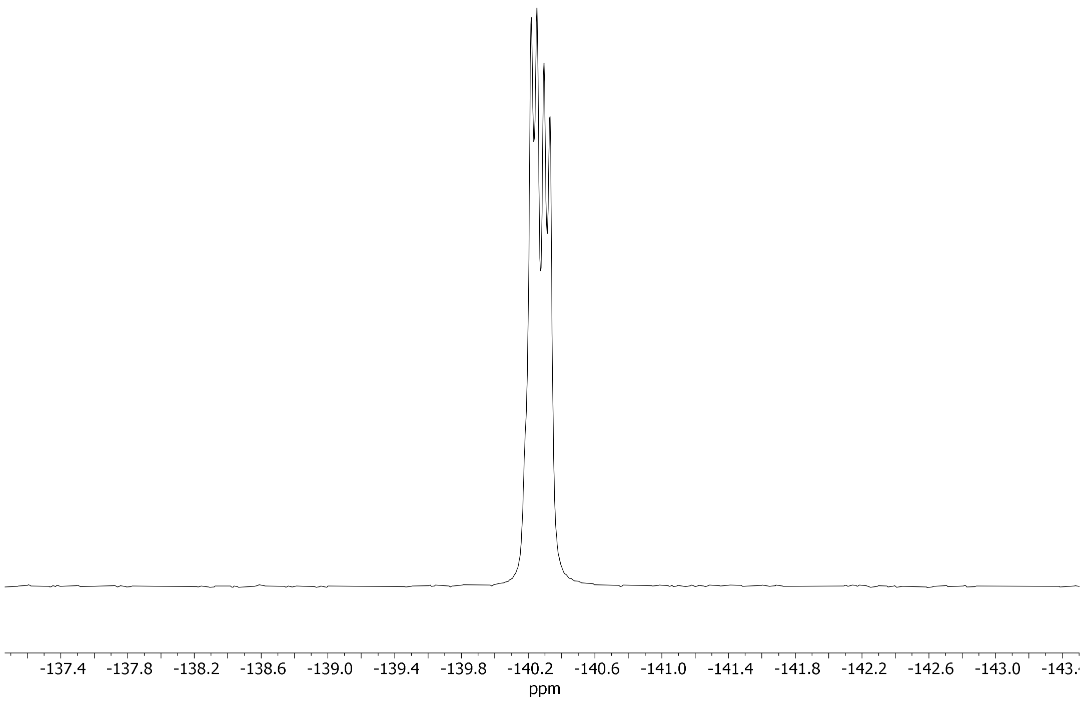

Figure S10. ^{19}F NMR (CD_2Cl_2, 25°C) of **^{Me}IPP_BF$_2$**.

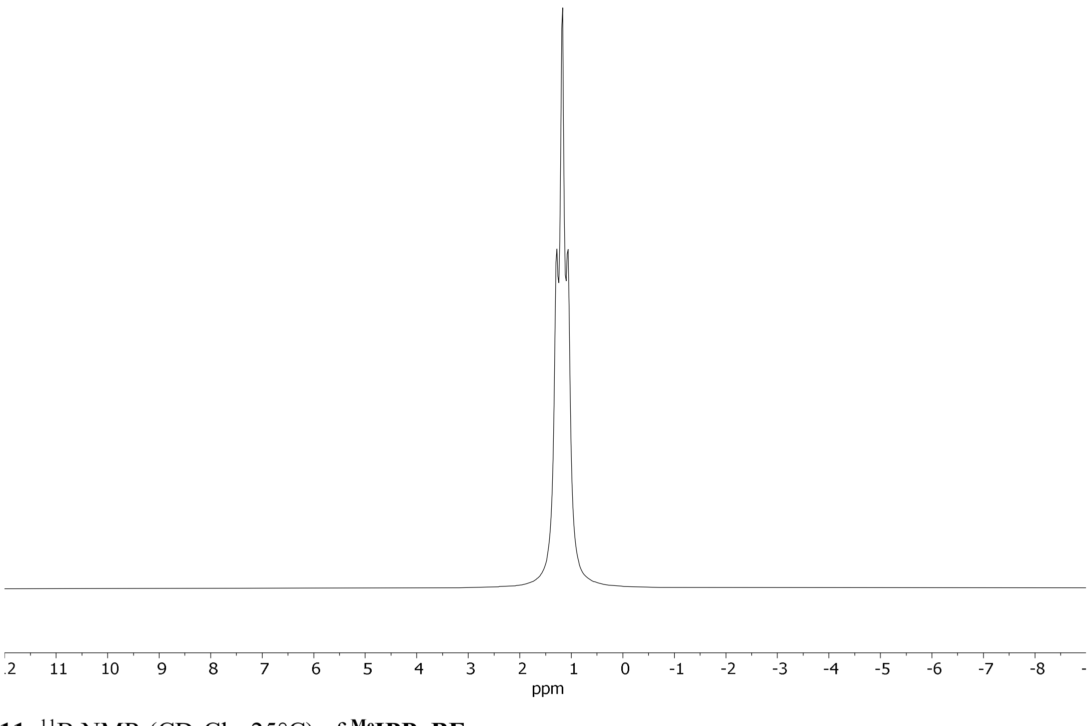

Figure S11. ^{11}B NMR (CD_2Cl_2, 25°C) of **^{Me}IPP_BF$_2$**.

^{OMe}IPP_BF$_2$

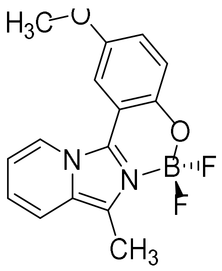

^{1}H NMR (400 MHz, CD_2Cl_2, 298 K, *J* [Hz]): δ = 8.62 – 8.52 (m, 1H), 7.68 – 7.59 (m, 1H), 7.43 (d, *J* = 2.8, 1H), 7.18 (d, *J* = 9.0, 1H), 7.07 (dd, *J* = 9.0, 2.9, 1H), 7.05 – 6.95 (m, 2H), 3.90 (s, 3H), 2.74 (s, 3H). ^{13}C NMR (100 MHz, CD_2Cl_2, 298 K): δ = 152.76, 149.05, 127.71, 122.72, 122.29, 120.71, 120.61, 119.04, 117.53, 117.25, 110.82, 107.63, 56.06, 9.36. ^{19}F NMR (376 MHz, CD_2Cl_2, 298 K, *J* [Hz]): δ = -140.71 (q, *J* = 13.1).^{11}B NMR (128 MHz, CD_2Cl_2, 298 K, *J* [Hz]): δ = 1.15 (t, *J* = 14.8).

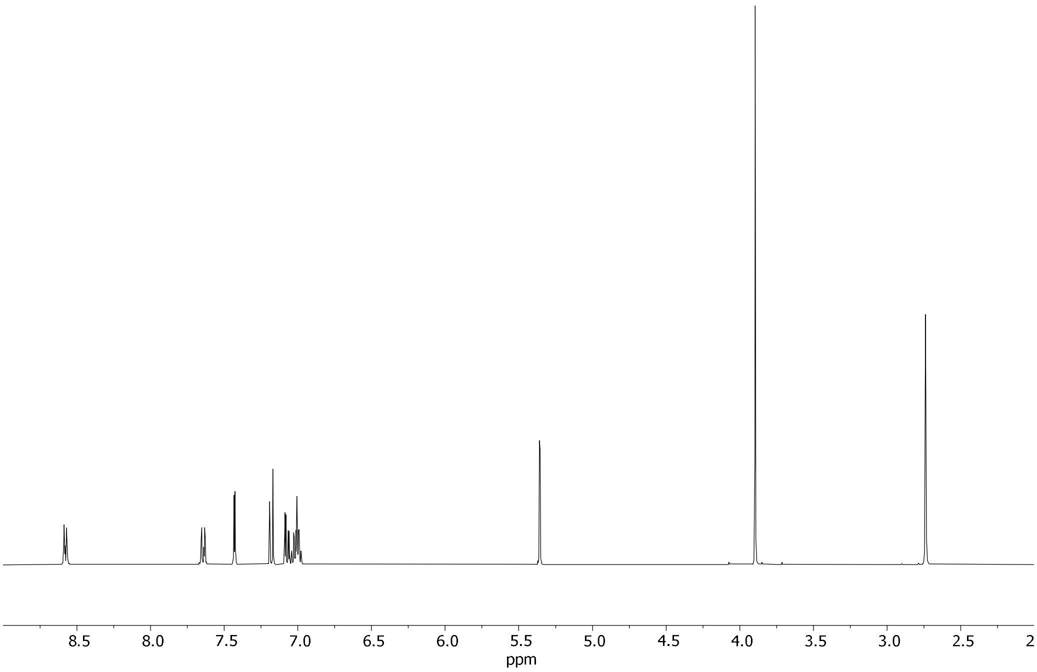

Figure S12. ^{1}H NMR (CD_2Cl_2, 25°C) of **^{OMe}IPP_BF$_2$**.

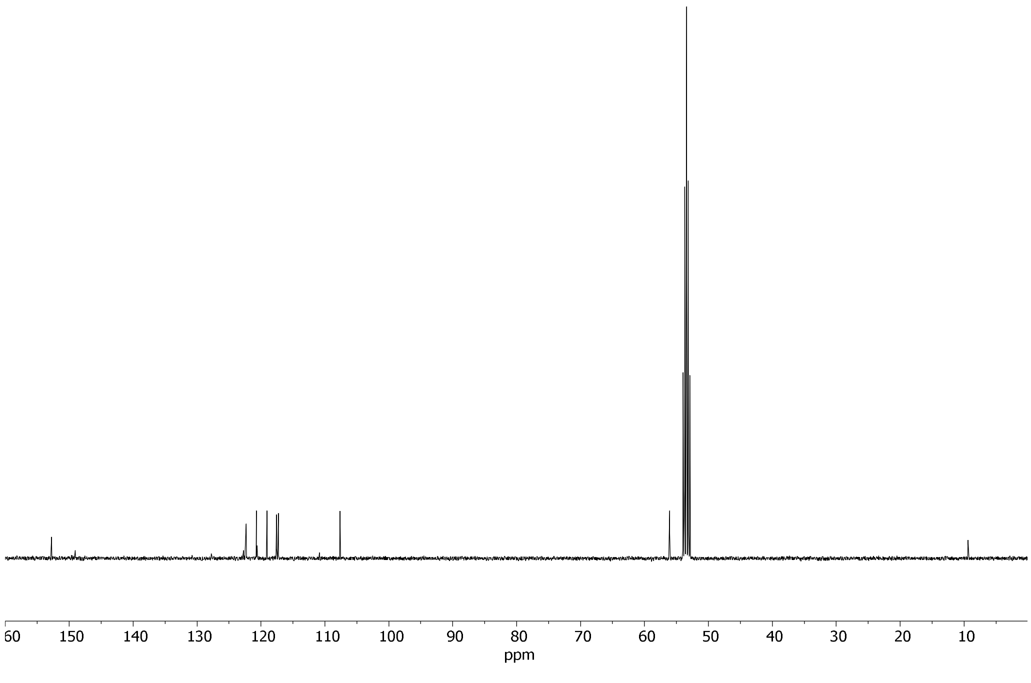

Figure S13. ^{13}C NMR (CD_2Cl_2, 25°C) of **^{OMe}TIPP_BF$_2$**.

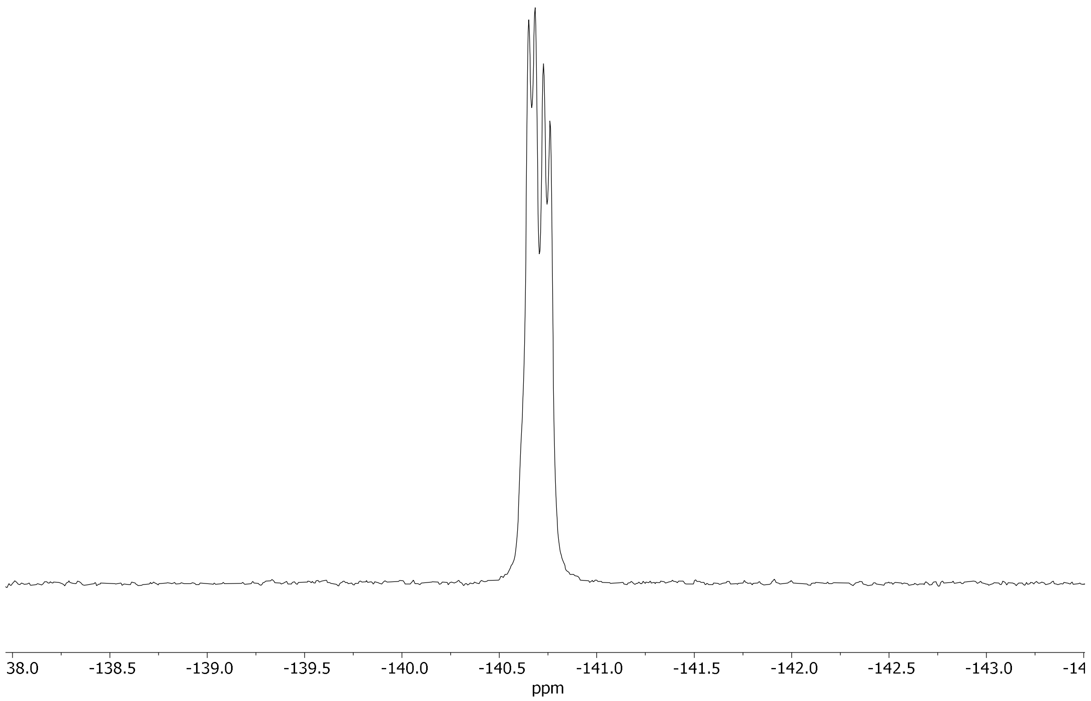

Figure S14. ^{19}F NMR (CD_2Cl_2, 25°C) of $^{\mathbf{OMe}}$**IPP_BF$_2$**.

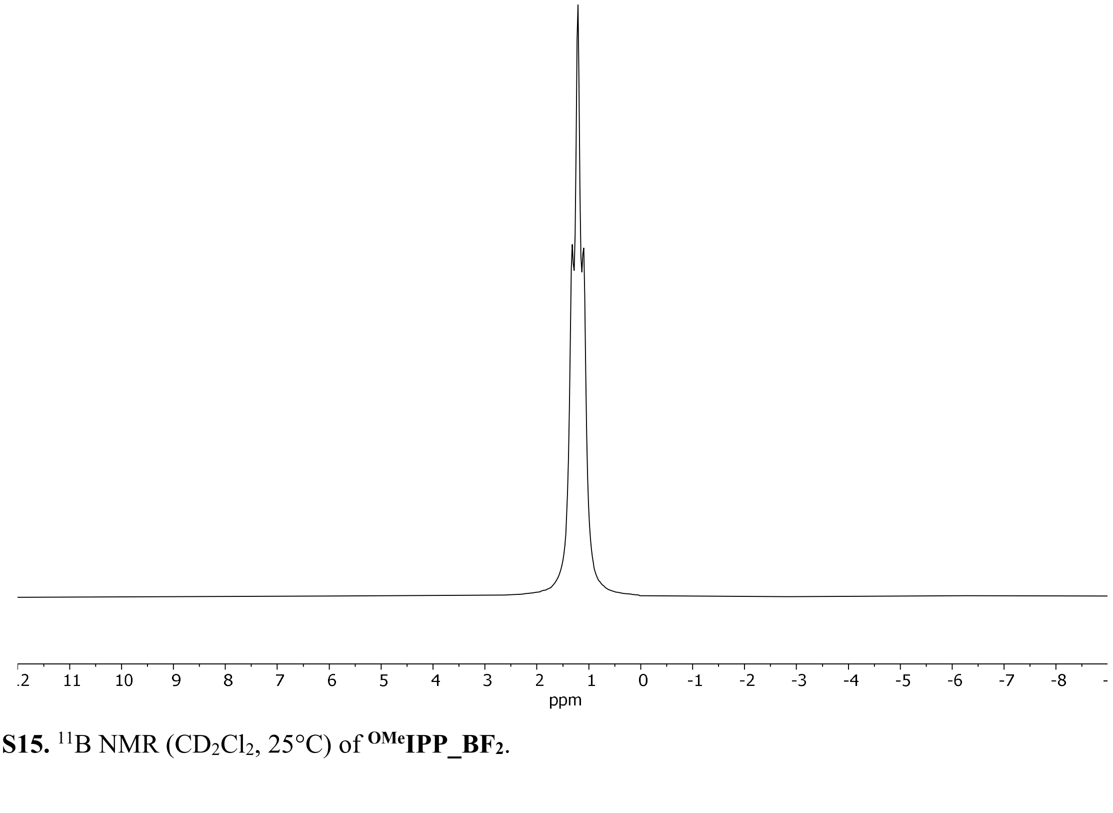

Figure S15. ^{11}B NMR (CD_2Cl_2, 25°C) of $^{\mathbf{OMe}}$**IPP_BF$_2$**.

^{F}IPP_BF$_2$

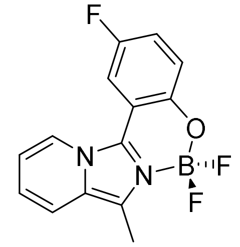

^{1}H NMR (400 MHz, CD_2Cl_2, 298 K, J [Hz]): δ = 8.58 – 8.47 (m, 1H), 7.82 – 7.54 (m, 2H), 7.25 – 7.17 (m, 2H), 7.14 – 7.00 (m, 2H), 2.76 (s, 3H). ^{13}C NMR (100 MHz, CD_2Cl_2, 298 K): δ = 157.02, 154.66, 128.00, 122.05, 121.06, 119.15, 118.68, 118.45, 117.68, 108.40, 108.14, 9.36. ^{19}F NMR (376 MHz, CD_2Cl_2, 298 K, J [Hz]): δ = -123.56, -140.17 (q, J = 13.1). ^{11}B NMR (128 MHz, CD_2Cl_2, 298 K, J [Hz]): δ = 1.17 (t, J = 14.4).

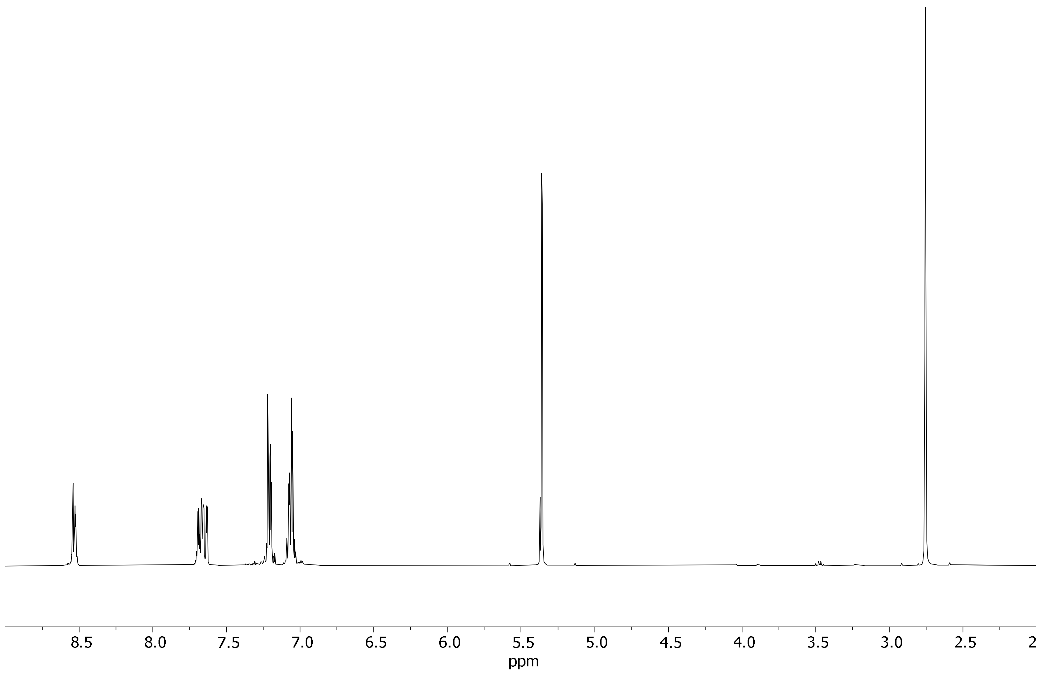

Figure S16. ^{1}H NMR (CD_2Cl_2, 25°C) of **^{F}IPP_BF$_2$**.

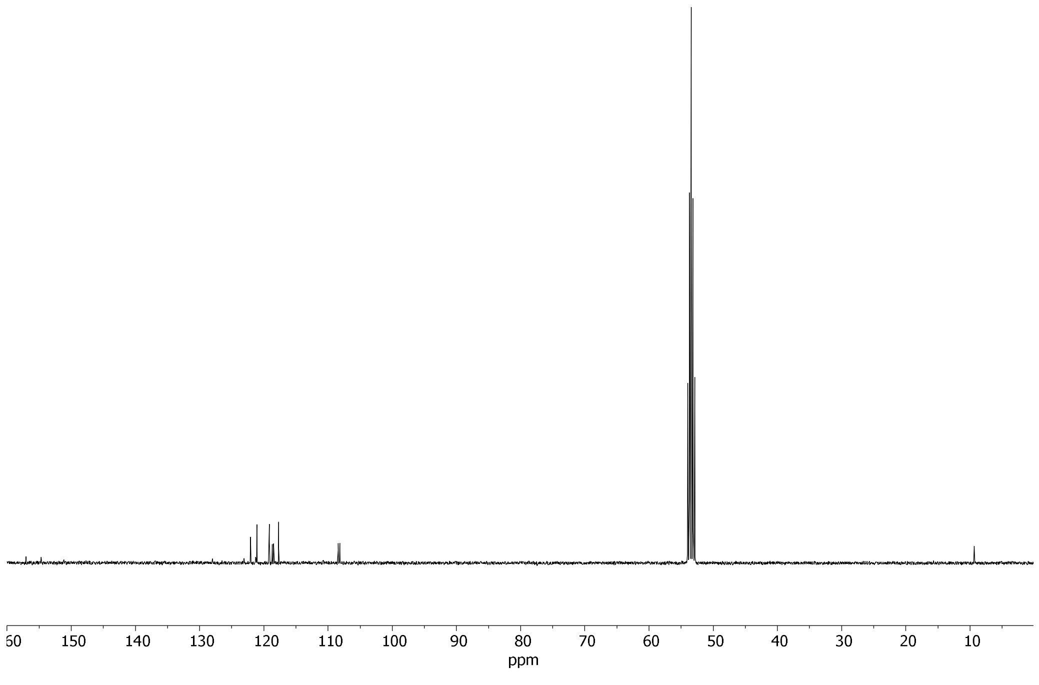

Figure S17. ^{13}C NMR (CD_2Cl_2, 25°C) of **^{F}IPP_BF$_2$**.

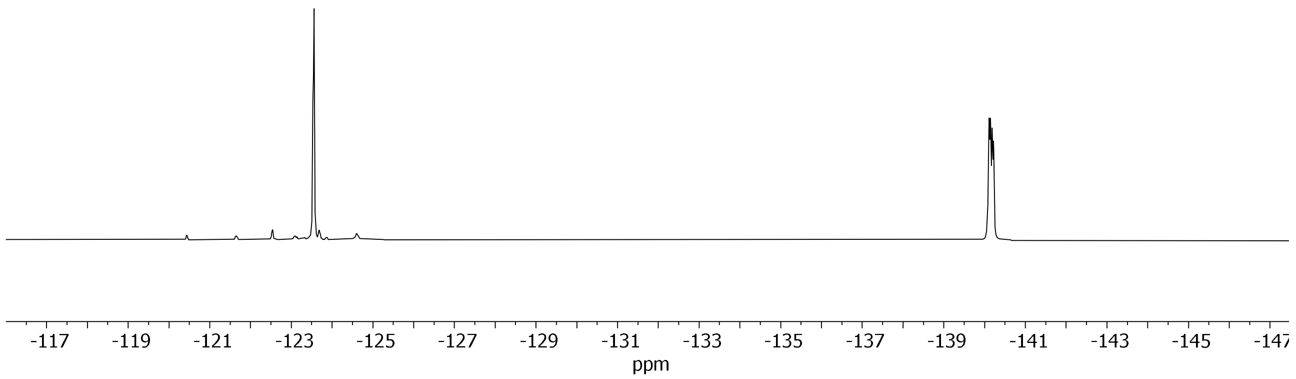

Figure S18. ^{19}F NMR (CD_2Cl_2, 25°C) of $^{\mathbf{F}}$**IPP_BF$_2$**.

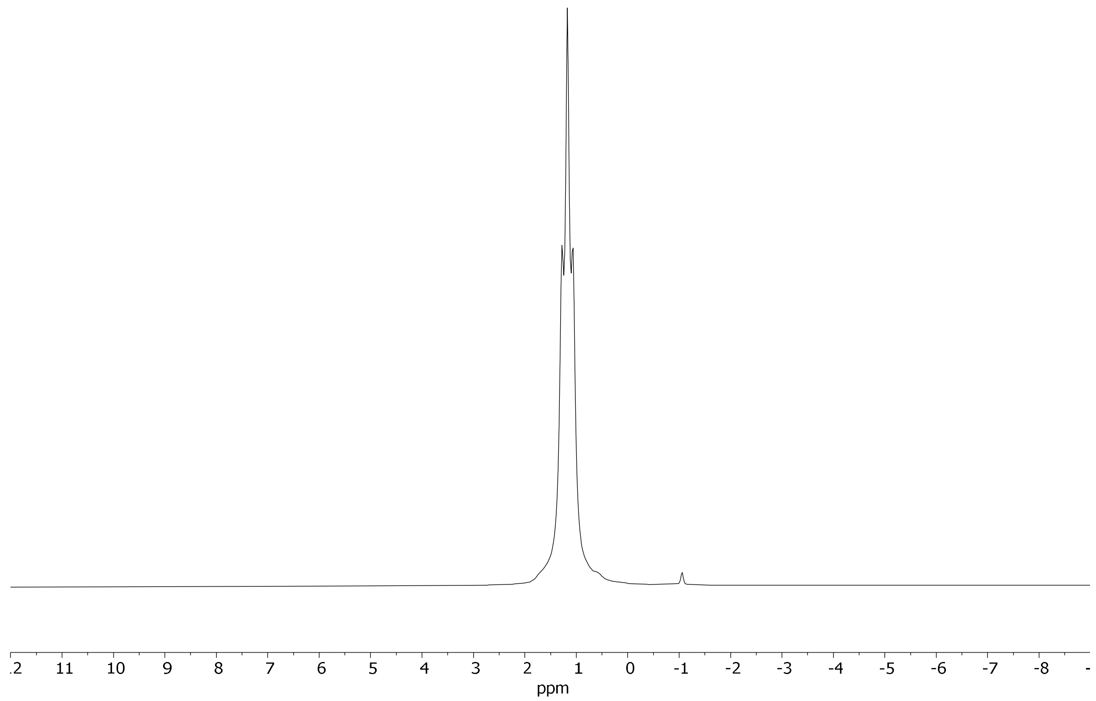

Figure S19. ^{11}B NMR (CD_2Cl_2, 25°C) of $^{\mathbf{F}}$**IPP_BF$_2$**.

^{Br}IPP_BF$_2$

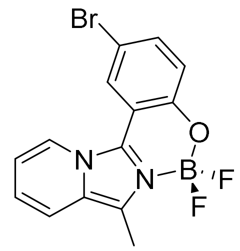

^{1}H NMR (400 MHz, CD_2Cl_2, 298 K, *J* [Hz]): δ = 8.54 – 8.37 (m, 1H), 7.92 (d, *J* = 2.3, 1H), 7.67 – 7.49 (m, 1H), 7.43 (dd, *J* = 8.8, 2.3, 1H), 7.03 (d, *J* = 8.8, 1H), 6.99 – 6.86 (m, 2H), 2.63 (s, 3H). ^{13}C NMR (100 MHz, CD_2Cl_2, 298 K): δ = 153.95, 134.37, 128.05, 124.49, 123.15, 122.22, 121.90, 121.14, 119.13, 117.76, 111.51, 9.36. ^{19}F NMR (376 MHz, CD_2Cl_2, 298 K, *J* [Hz]): δ = -139.89 (q, *J* = 12.7). ^{11}B NMR (128 MHz, CD_2Cl_2, 298 K, *J* [Hz]): δ = 1.03 (t, *J* = 14.4).

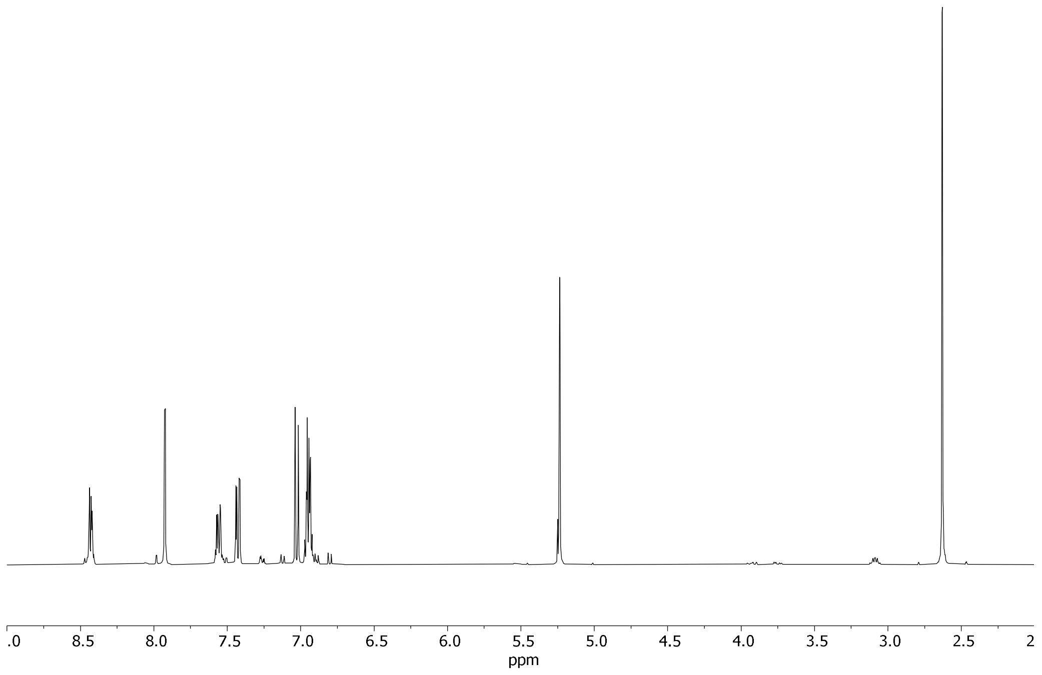

Figure S20. ^{1}H NMR (CD_2Cl_2, 25°C) of **^{Br}IPP_BF$_2$**.

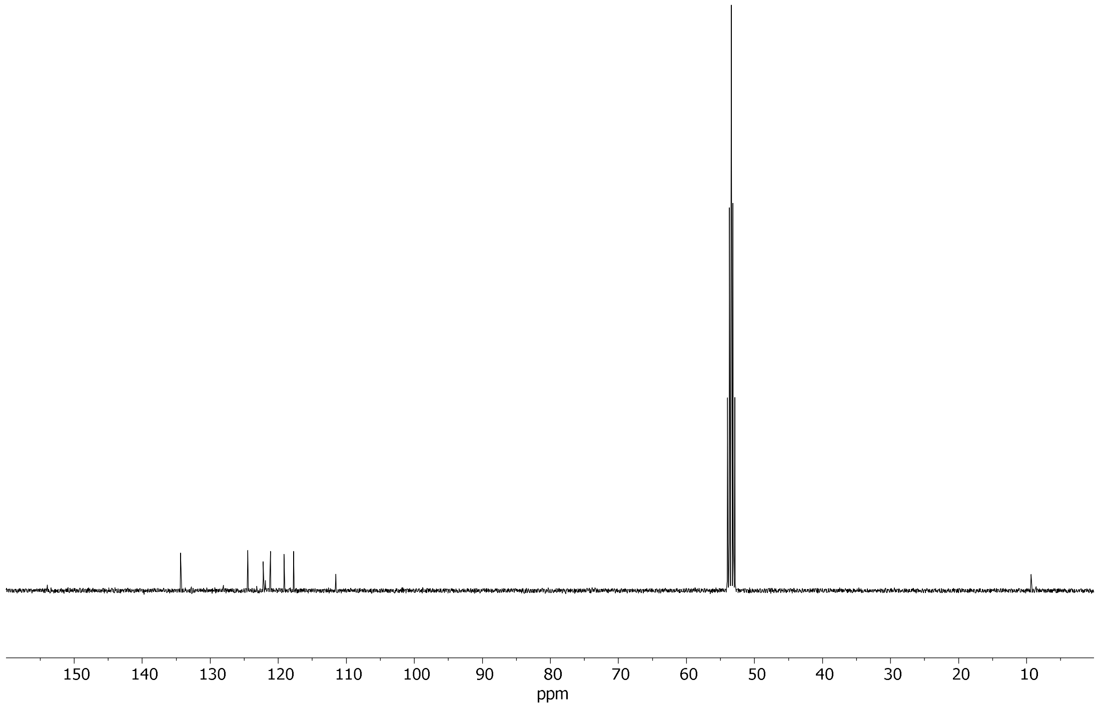

Figure S21. ^{13}C NMR (CD_2Cl_2, 25°C) of **^{Br}IPP_BF$_2$**.

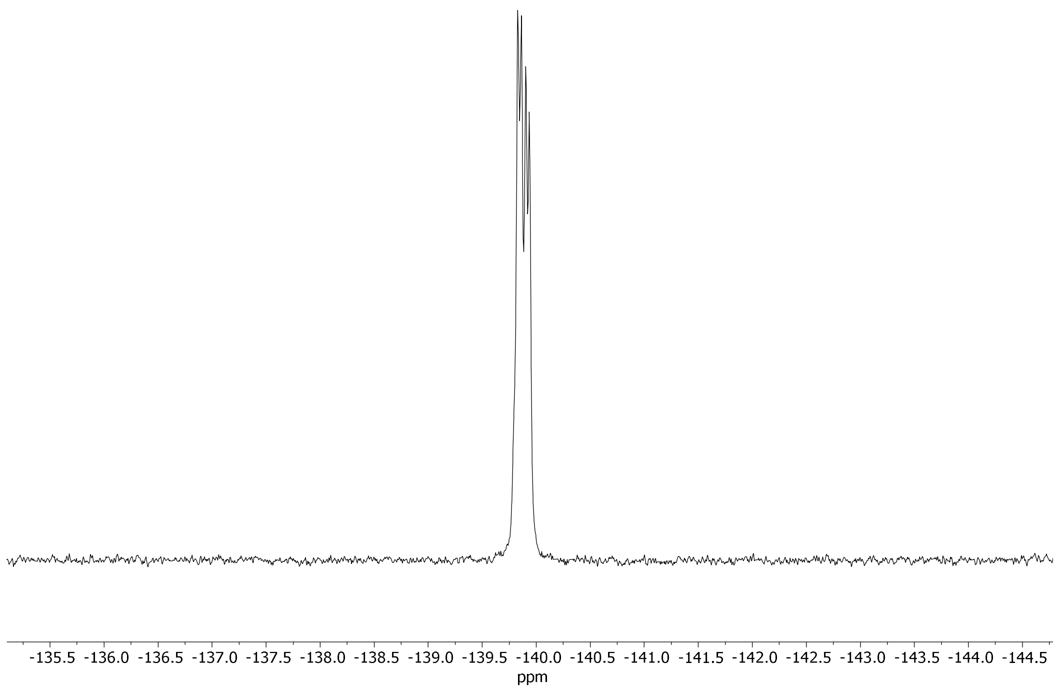

Figure S22. ^{19}F NMR (CD_2Cl_2, 25°C) of **^{Br}IPP_BF$_2$**.

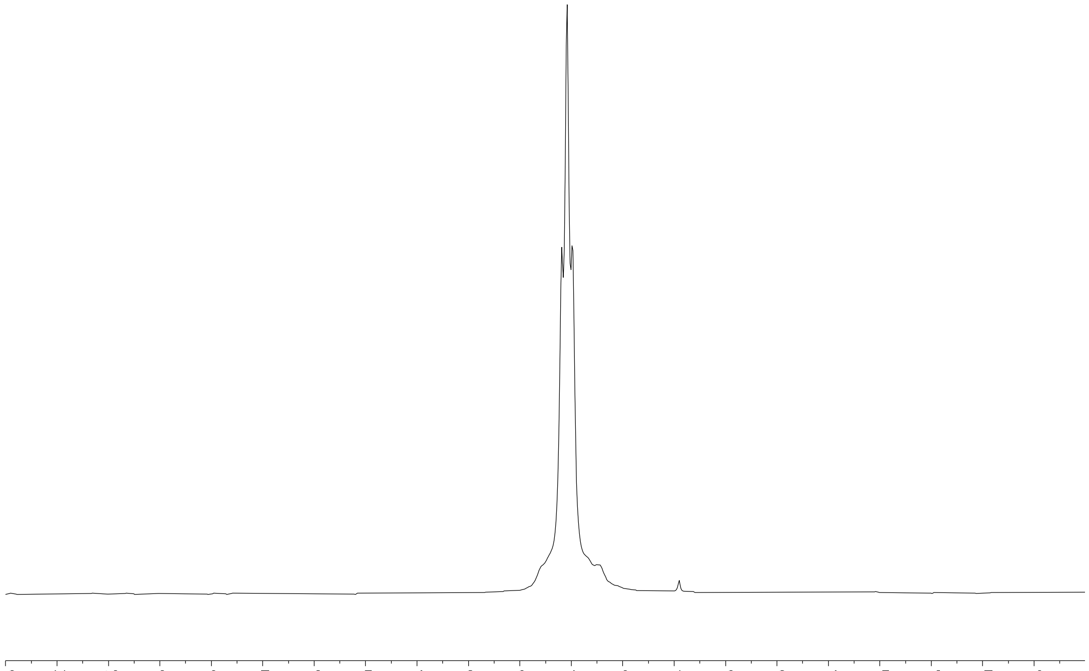

Figure S23. ^{11}B NMR (CD_2Cl_2, 25°C) of **^{Br}IPP_BF$_2$**.

^{NO2}IPP_BF$_2$

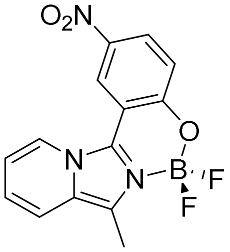

^{1}H (400 MHz, CD_2Cl_2, 298 K, *J* [Hz]): δ = 8.91 (d, *J* = 2.4, 1H), 8.70 – 8.67 (m, 1H), 7.86 – 7.67 (m, 1H), 7.34 (dd, *J* = 9.1, 1.3, 1H), 7.26 – 7.08 (m, 2H), 2.79 (s, 3H). ^{13}C NMR (100 MHz, CD_2Cl_2, 298 K): δ = 159.91, 140.25, 128.54, 122.16, 121.75, 121.15, 120.56, 119.26, 118.56, 118.40, 115.13, 114.98, 9.37. ^{19}F NMR (376 MHz, CD_2Cl_2, 298 K, *J* [Hz]): δ = -138.77 (q, *J* = 12.4). ^{11}B NMR (128 MHz, CD_2Cl_2, 298 K, *J* [Hz]): δ = 1.05 (t, *J* = 13.4).

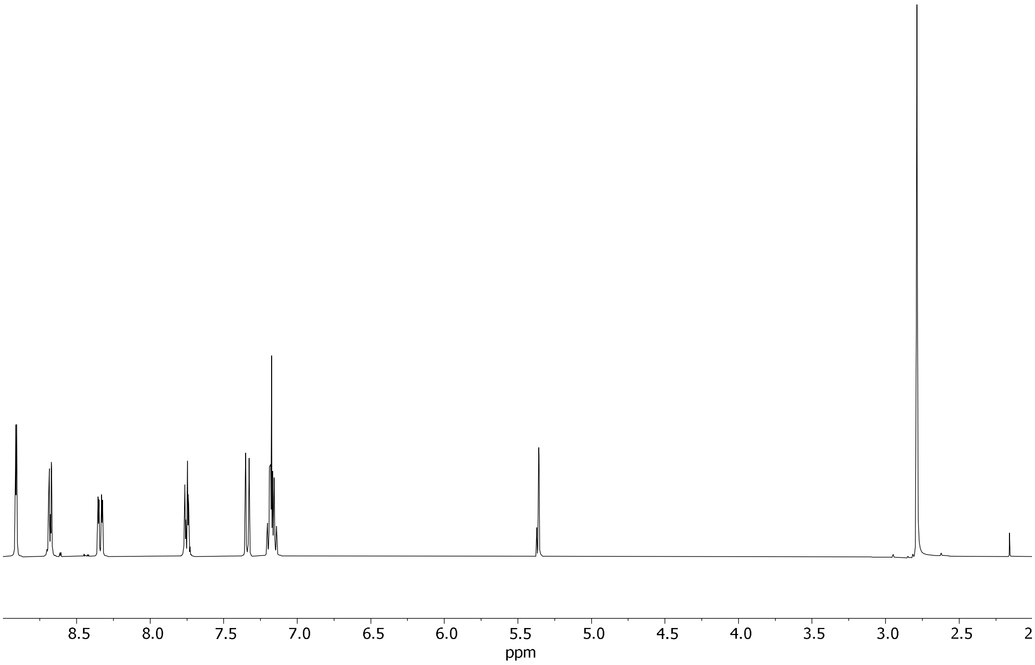

Figure S24 ^{1}H NMR (CD_2Cl_2, 25°C) of **^{NO2}IPP_BF$_2$**.

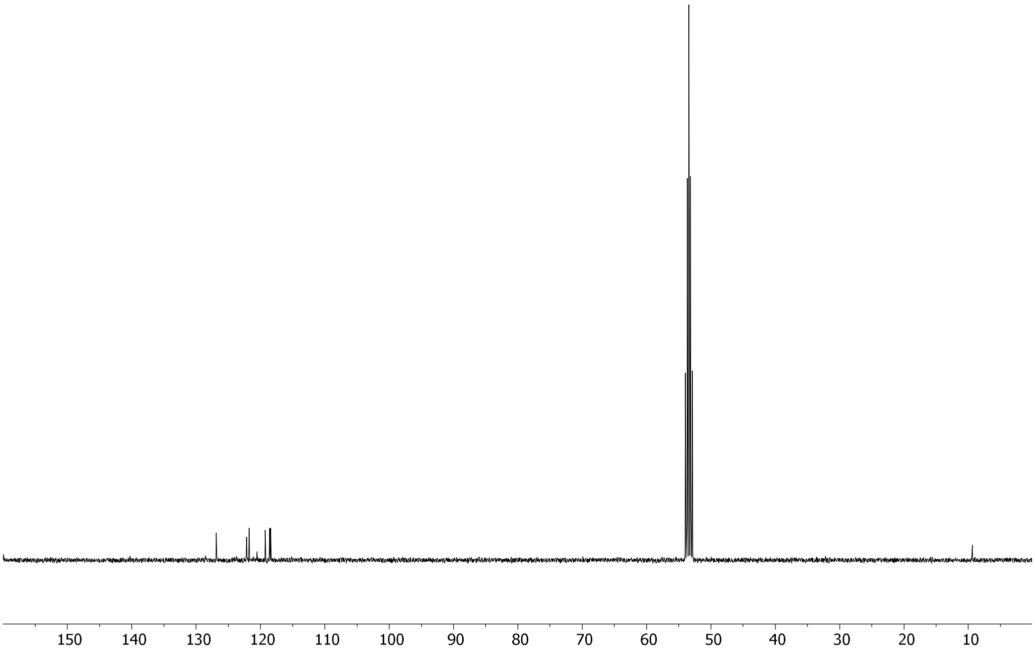

Figure S25. ^{13}C NMR (CD_2Cl_2, 25°C) of **^{NO2}IPP_BF$_2$**.

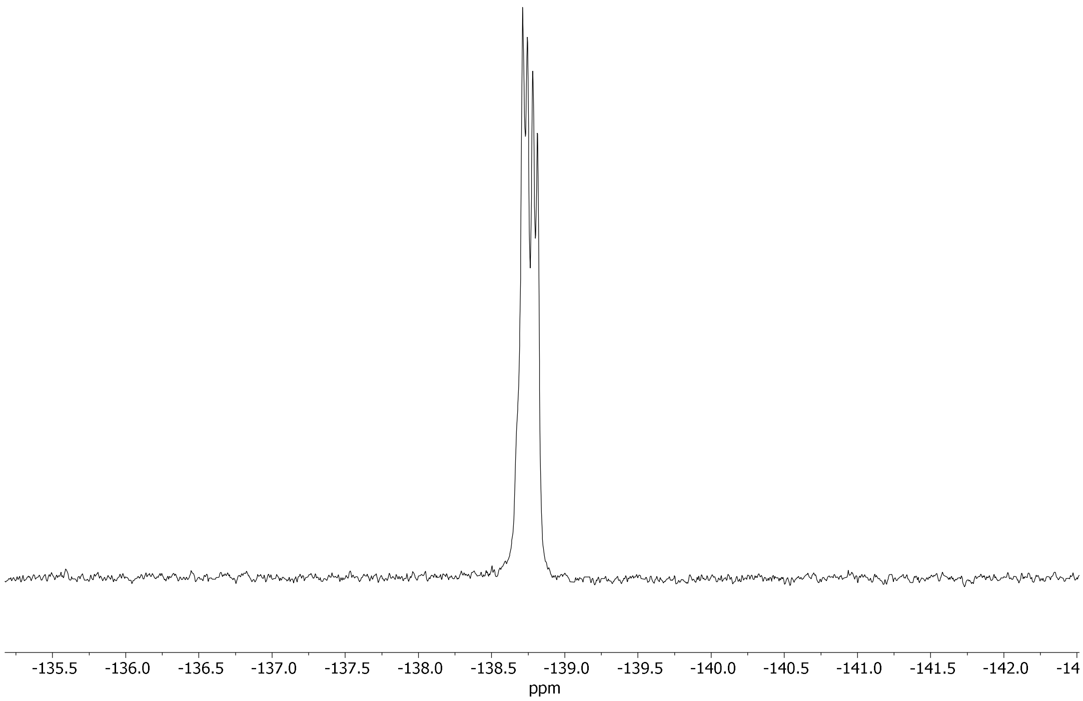

Figure S26. ^{19}F NMR (CD_2Cl_2, 25°C) of $^{\mathbf{NO2}}$**IPP_BF**$_2$.

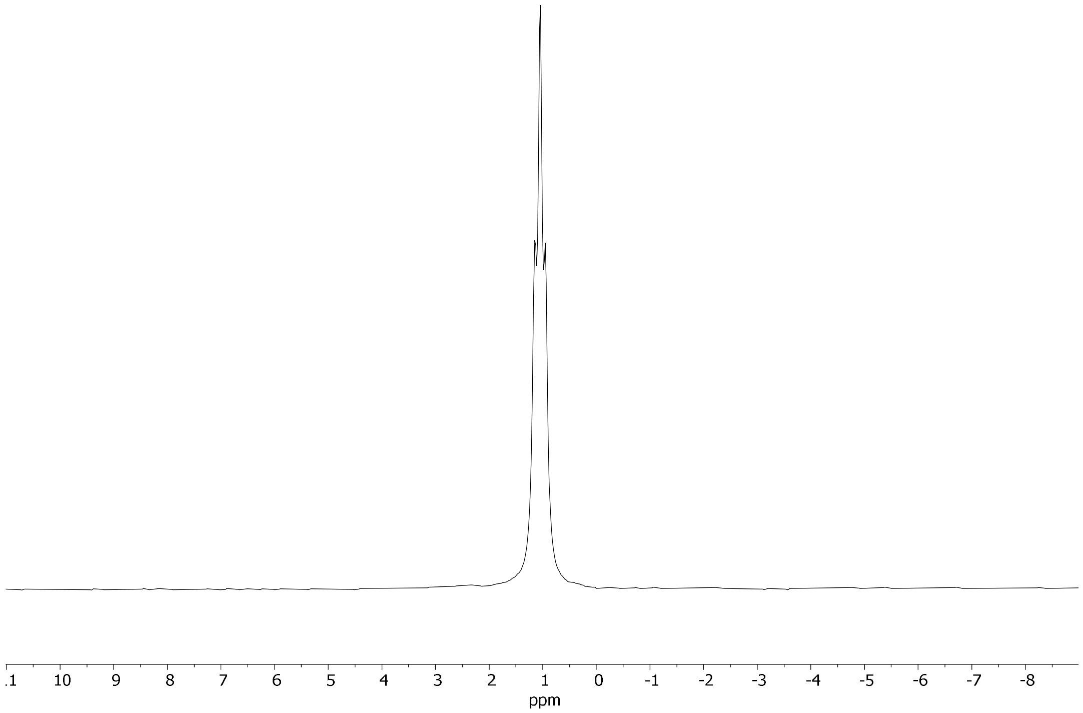

Figure S27. ^{11}B NMR (CD_2Cl_2, 25°C) of $^{\mathbf{NO2}}$**IPP_BF**$_2$.

^{I}IPP_BF$_2$

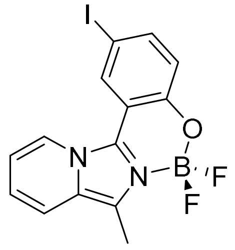

^{1}H NMR (400 MHz, CD_2Cl_2, 298 K, *J* [Hz]): δ = 8.49 – 8.41 (m, 1H), 8.07 (d, *J* = 2.1, 1H), 7.69 – 7.54 (m, 2H), 7.09 – 6.92 (m, 3H), 2.73 (s, 3H). ^{13}C NMR (100 MHz, CD_2Cl_2, 298 K): δ = 154.71, 140.39, 130.12, 129.29, 127.82, 123.36, 122.78, 122.15, 120.96, 119.26, 117.74, 113.25, 80.89, 9.66. ^{19}F NMR (376 MHz, CD_2Cl_2, 298 K, *J* [Hz]): δ = 139.74 (q, *J* = 11.4). ^{11}B NMR (128 MHz, CD_2Cl_2, 298 K, *J* [Hz]): δ = 1.08 (t, *J* = 14.3).

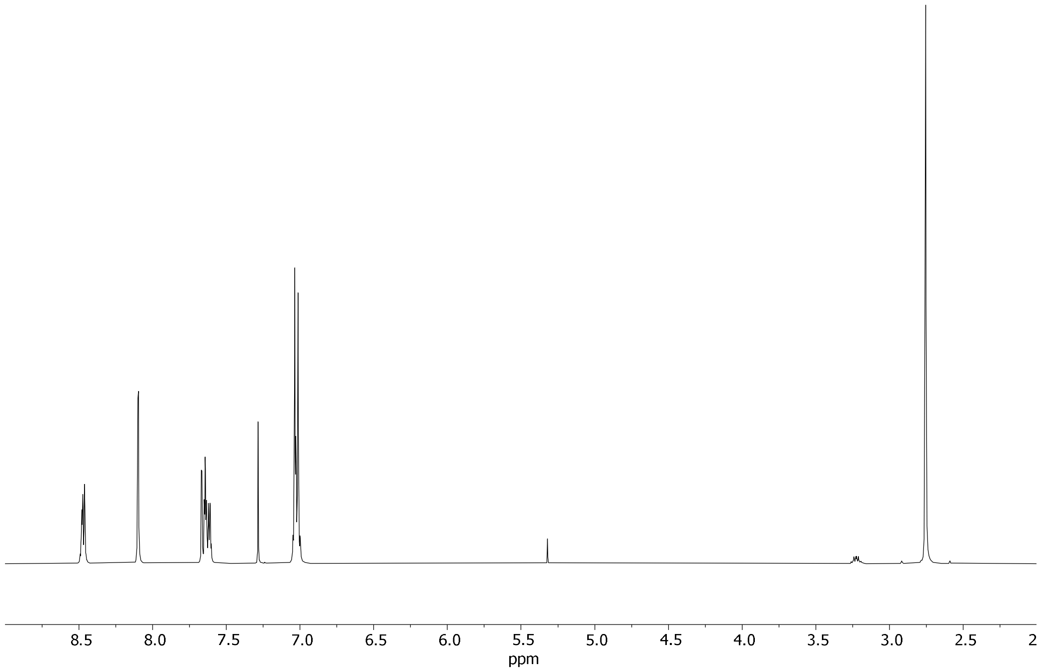

Figure S28. ^{1}H NMR ($CDCl_3$, 25°C) of **^{I}IPP_BF$_2$**.

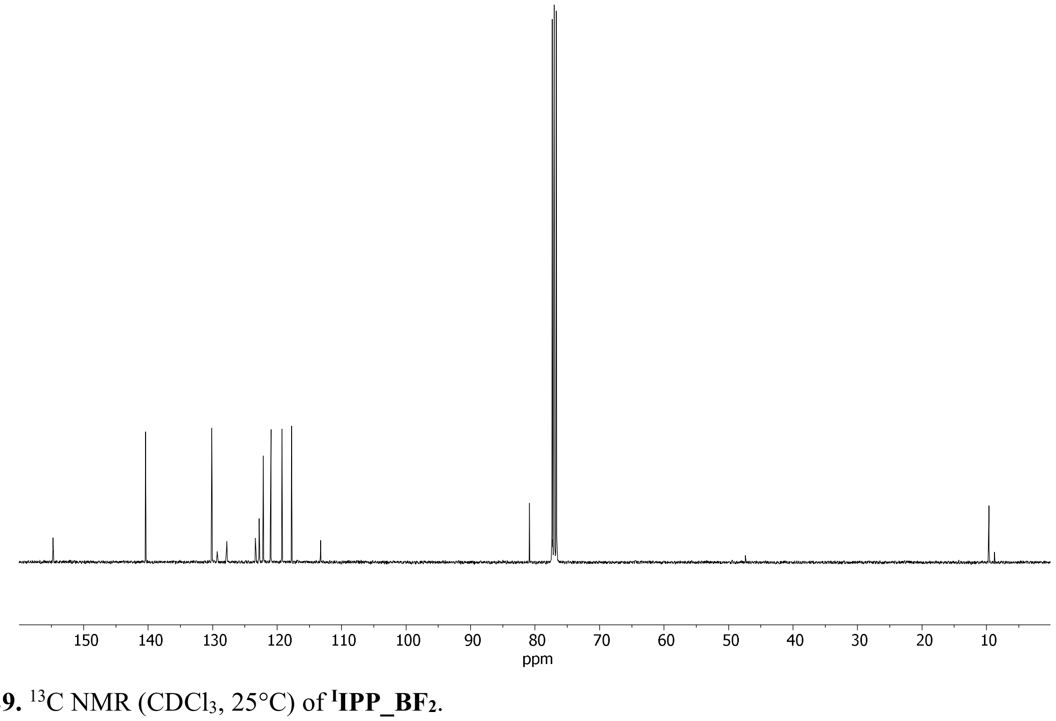

Figure S29. ^{13}C NMR ($CDCl_3$, 25°C) of **^{I}IPP_BF$_2$**.

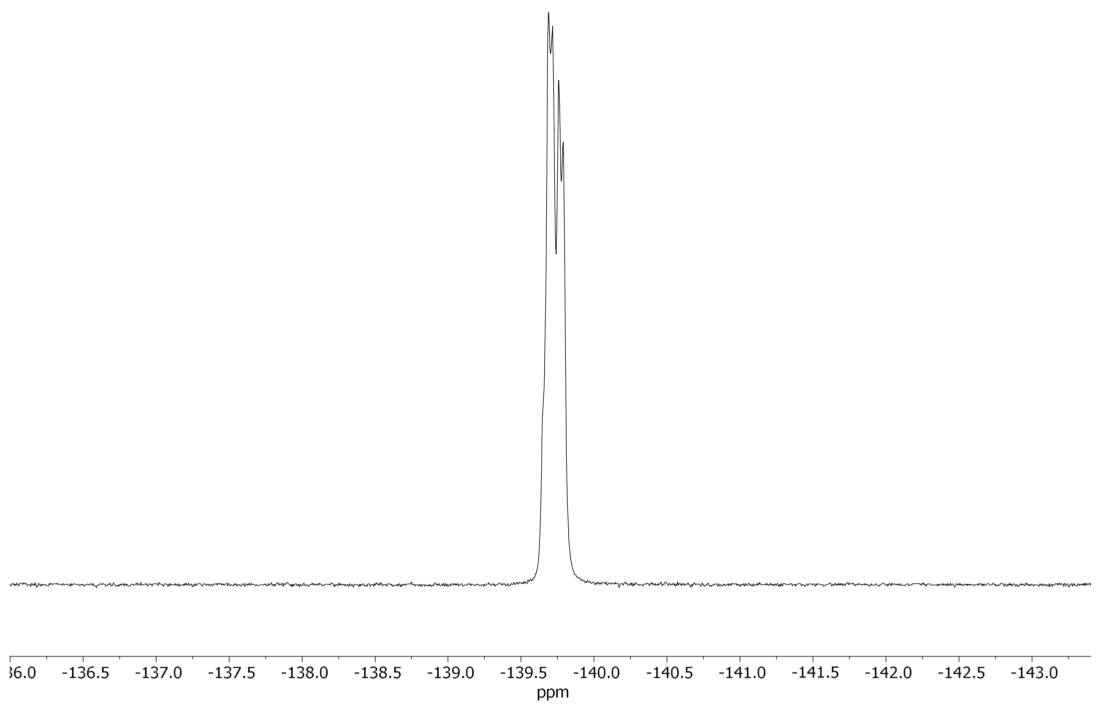

Figure S30. ^{19}F NMR ($CDCl_3$, 25°C) of **^{I}IPP_BF$_2$**.

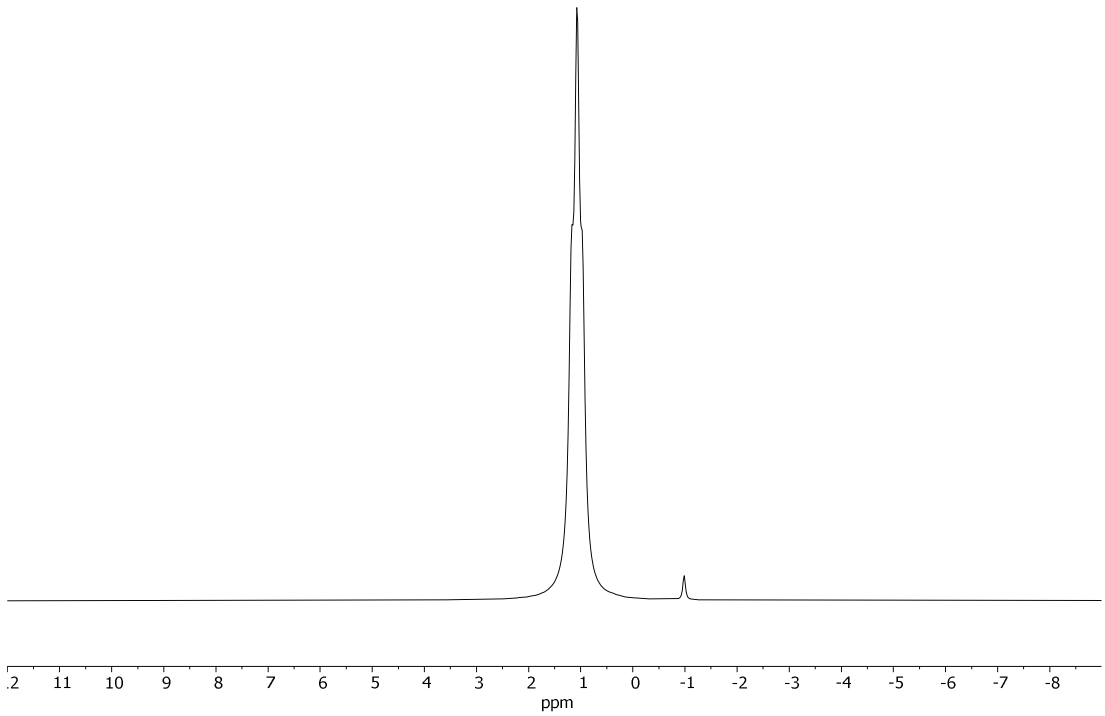

Figure S31. ^{11}B NMR ($CDCl_3$, 25°C) of **^{I}IPP_BF$_2$**.

^{Cl}IPP_BF$_2$

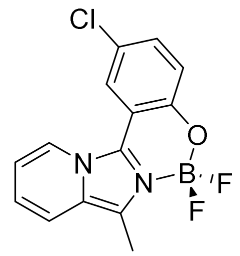

^{1}H NMR (400 MHz, $CDCl_3$, 298 K, *J* [Hz]): δ = 8.68 – 8.32 (m, 1H), 7.78 (m, 1H), 7.66 – 7.54 (m, 1H), 7.34 (dd, J = 8.9, 2.4, 1H), 7.18 (d, J = 8.9, 1H), 7.06 – 6.92 (m, 2H), 2.74 (s, 3H). ^{13}C NMR (100 MHz, CD_2Cl_2, 298 K): δ = 153.66, 131.70, 129.73, 127.83, 124.61, 123.41, 122.14, 121.93, 121.37, 121.05, 119.29, 117.71, 111.72, 9.64. ^{19}F NMR (376 MHz, CD_2Cl_2, 298 K, *J* [Hz]): δ = -139.80 (q, *J* = 11.4). ^{11}B NMR (128 MHz, CD_2Cl_2, 298 K, *J* [Hz]): δ = 1.14 (t, *J* = 14.2).

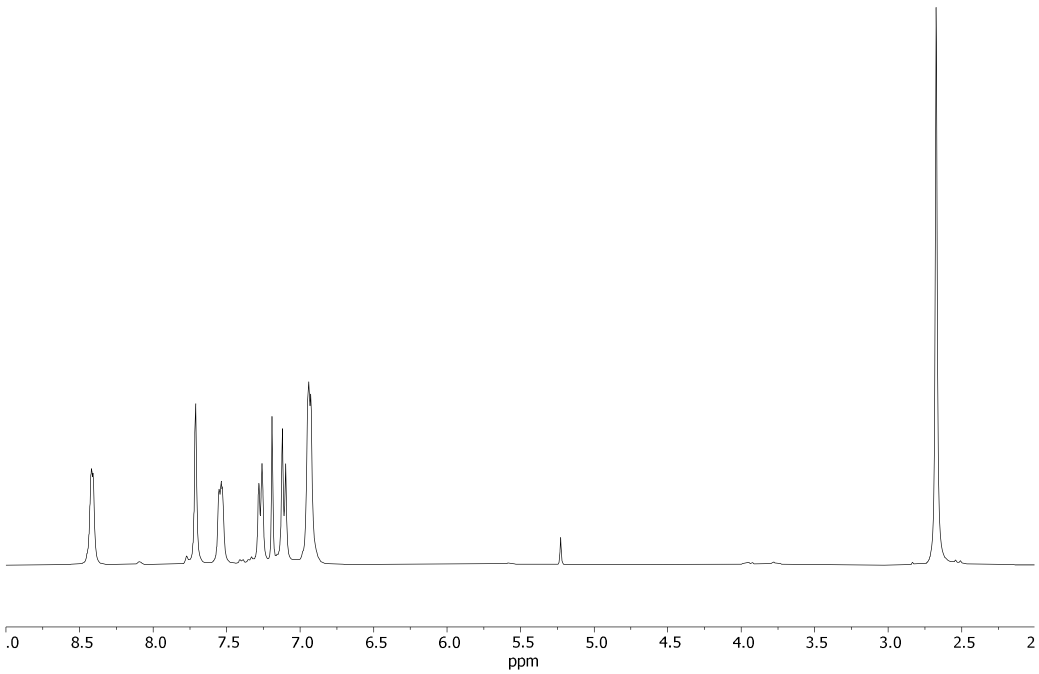

Figure S32. ^{1}H NMR ($CDCl_3$, 25°C) of **^{Cl}IPP_BF$_2$**.

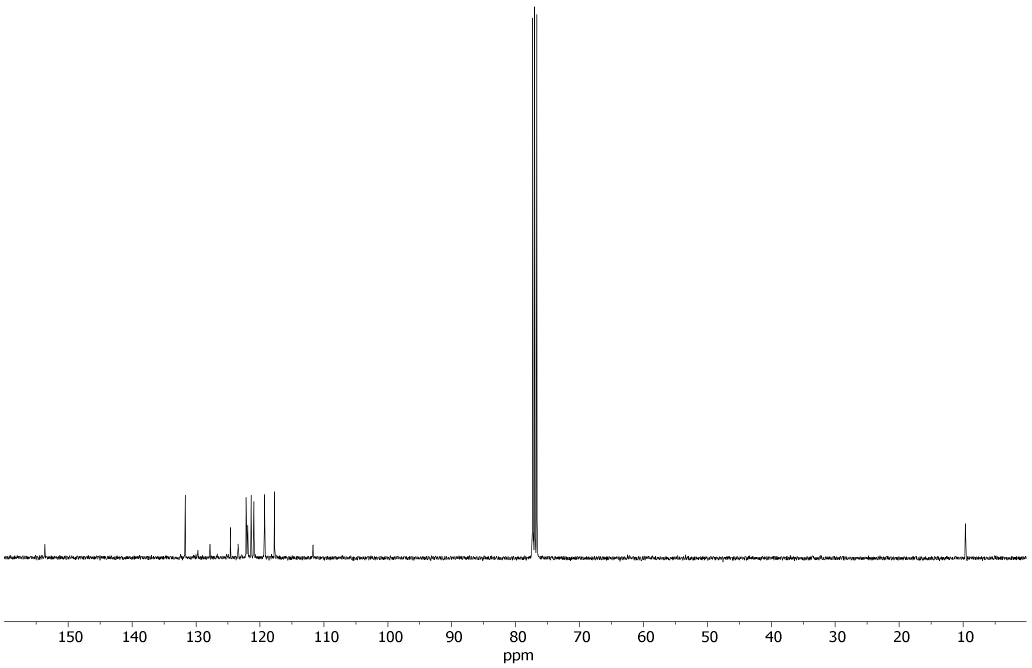

Figure S33. ^{13}C NMR ($CDCl_3$, 25°C) of **^{Cl}IPP_BF$_2$**.

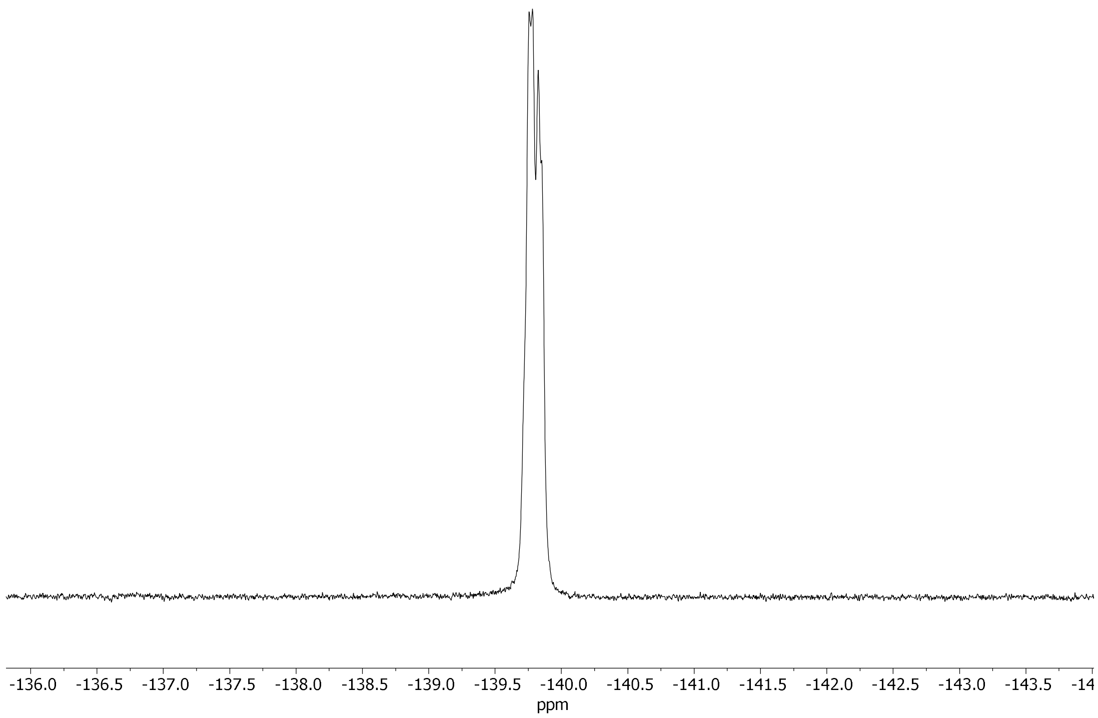

Figure S34. ^{19}F NMR ($CDCl_3$, 25°C) of ^{Cl}**IPP_BF$_2$**.

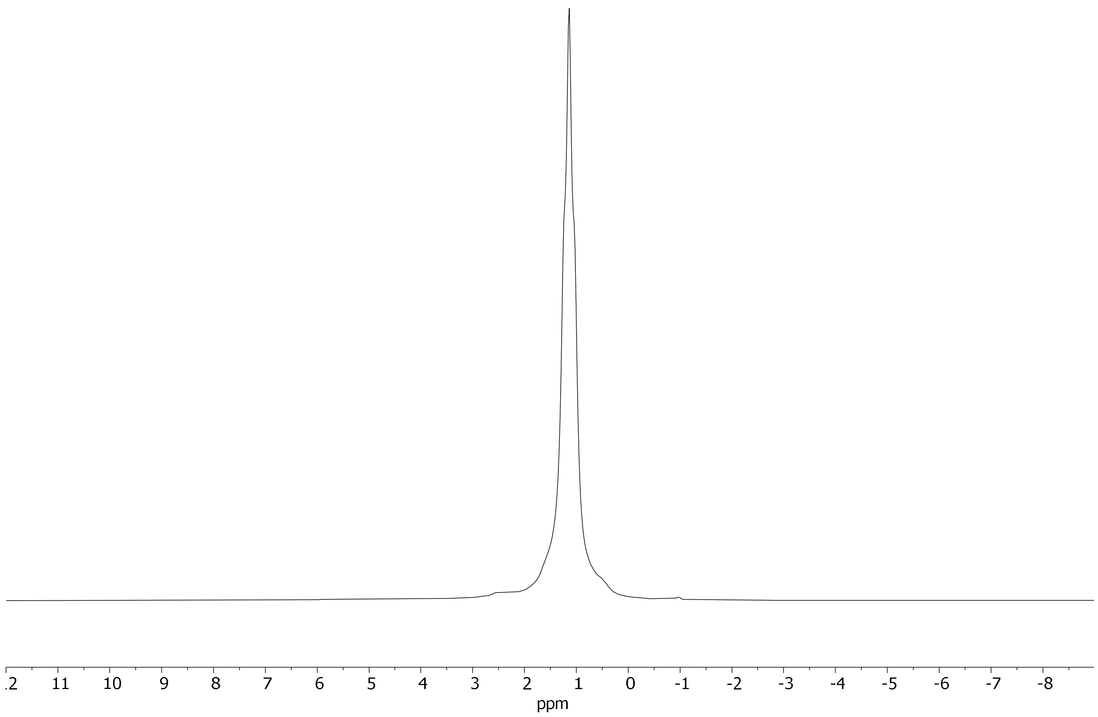

Figure S35. ^{11}B NMR ($CDCl_3$, 25°C) of ^{Cl}**IPP_BF$_2$**

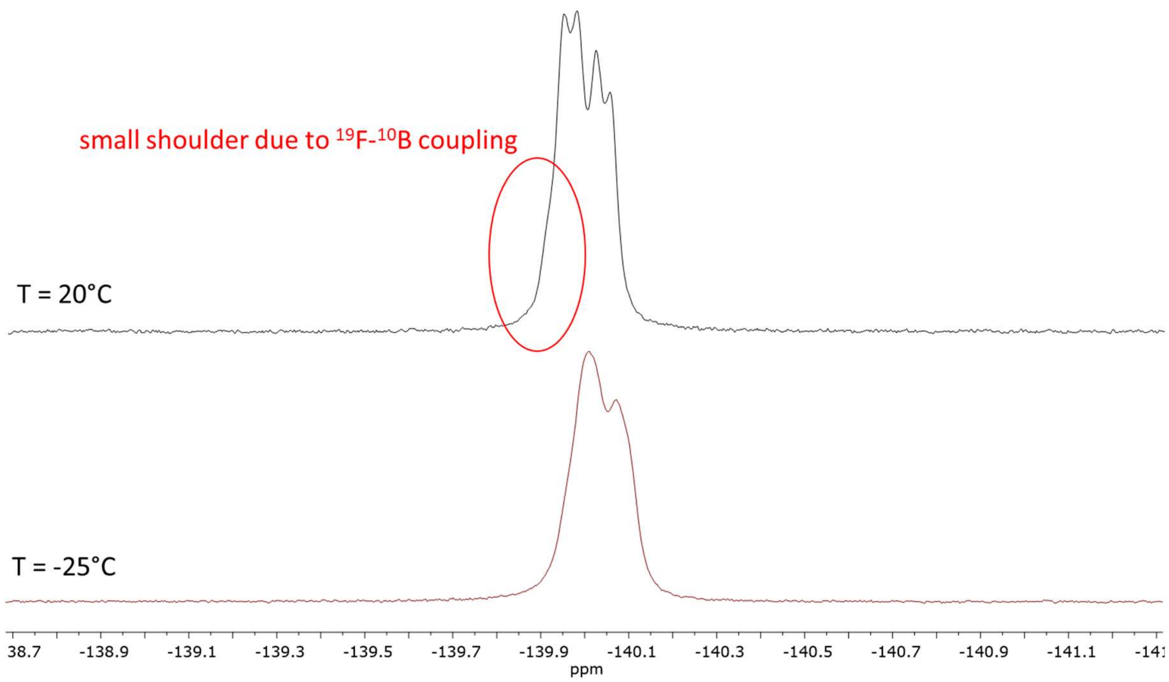

Figure S36. ^{19}F NMR spectrum of compound $^{\mathbf{H}}$**IPP_BF$_2$** recorded at -25°C ($CDCl_3$).

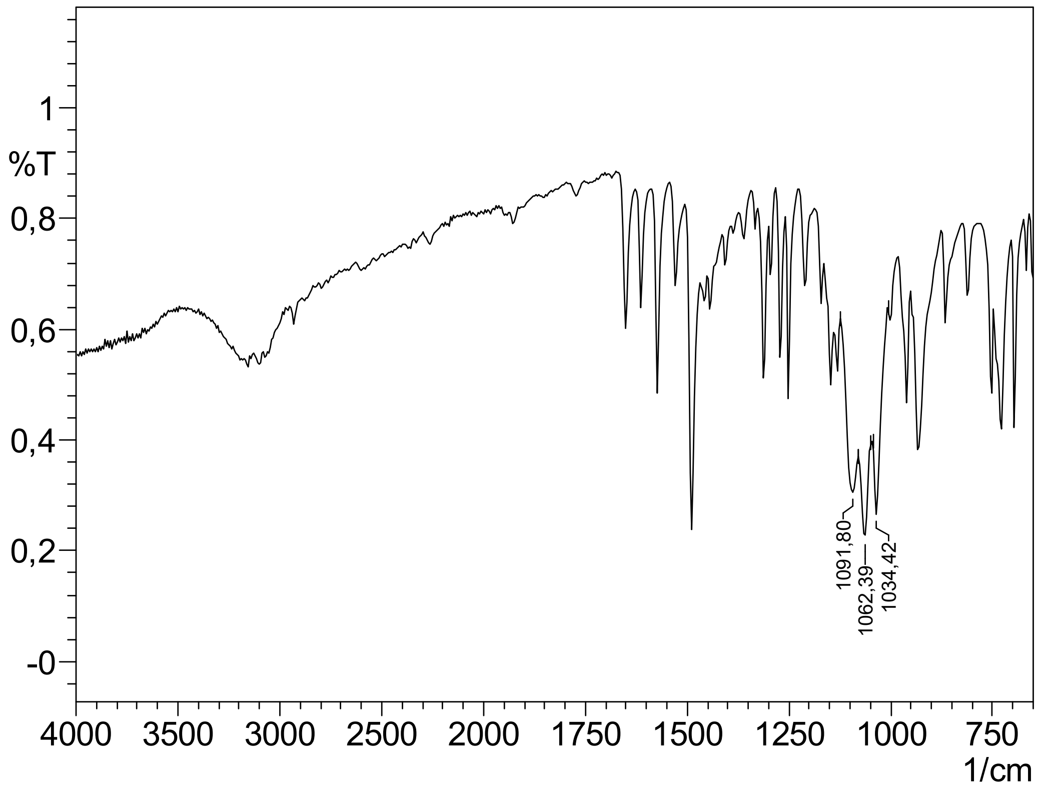

Figure S37. Infrared spectrum (ATR) of compound $^{\mathbf{H}}$**IPP_BF$_2$·*x*H$_2$O**.

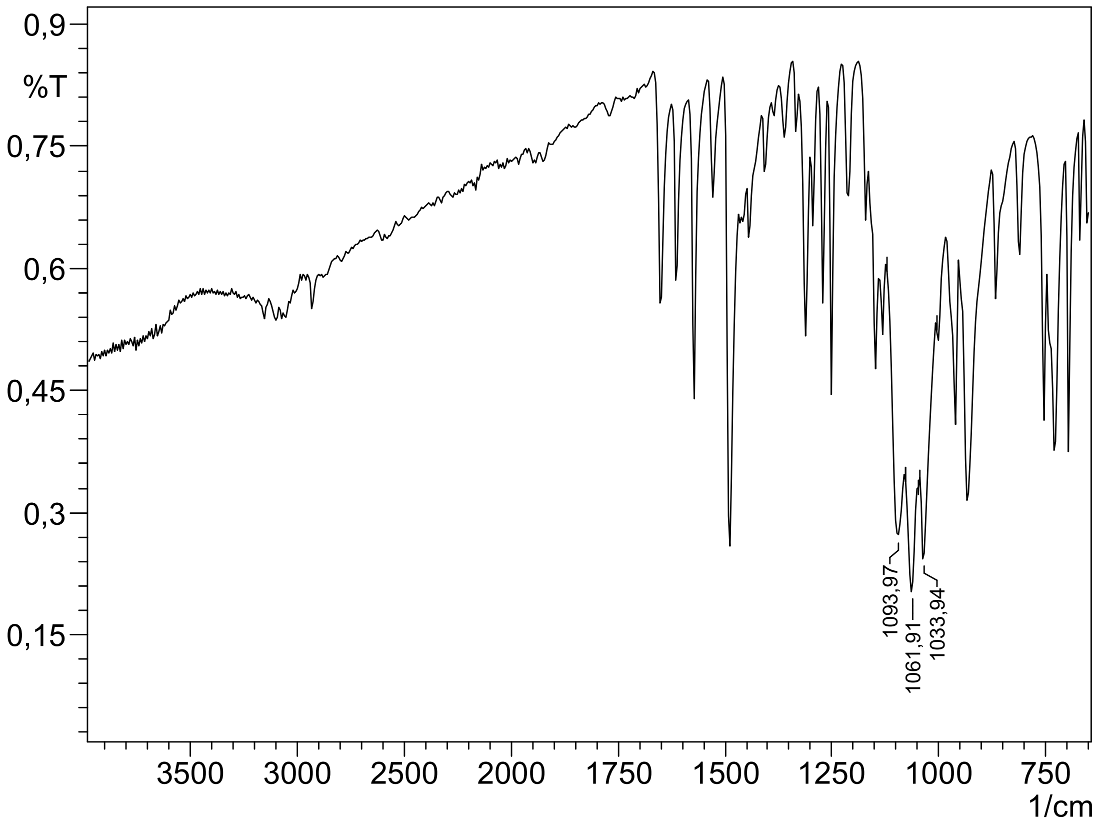

Figure S38. Infrared spectrum (ATR) of compound **^{H}IPP_BF$_2$**.

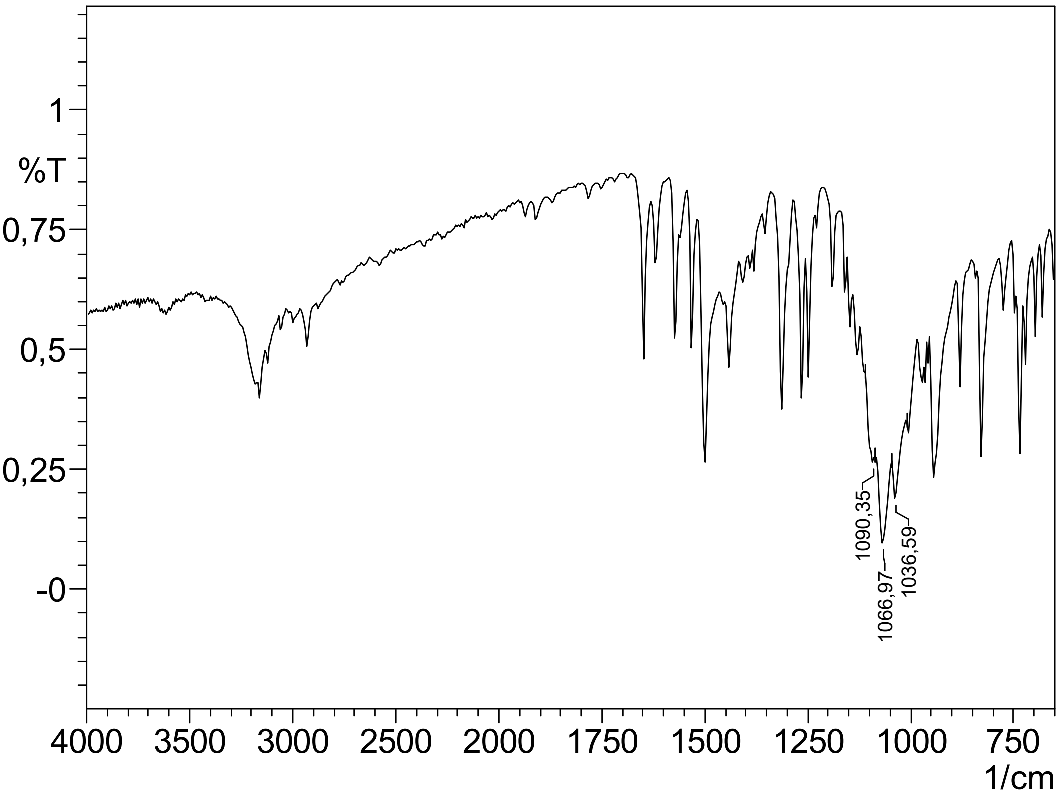

Figure S39. Infrared spectrum (ATR) of compound **^{Me}IPP_BF$_2$**.

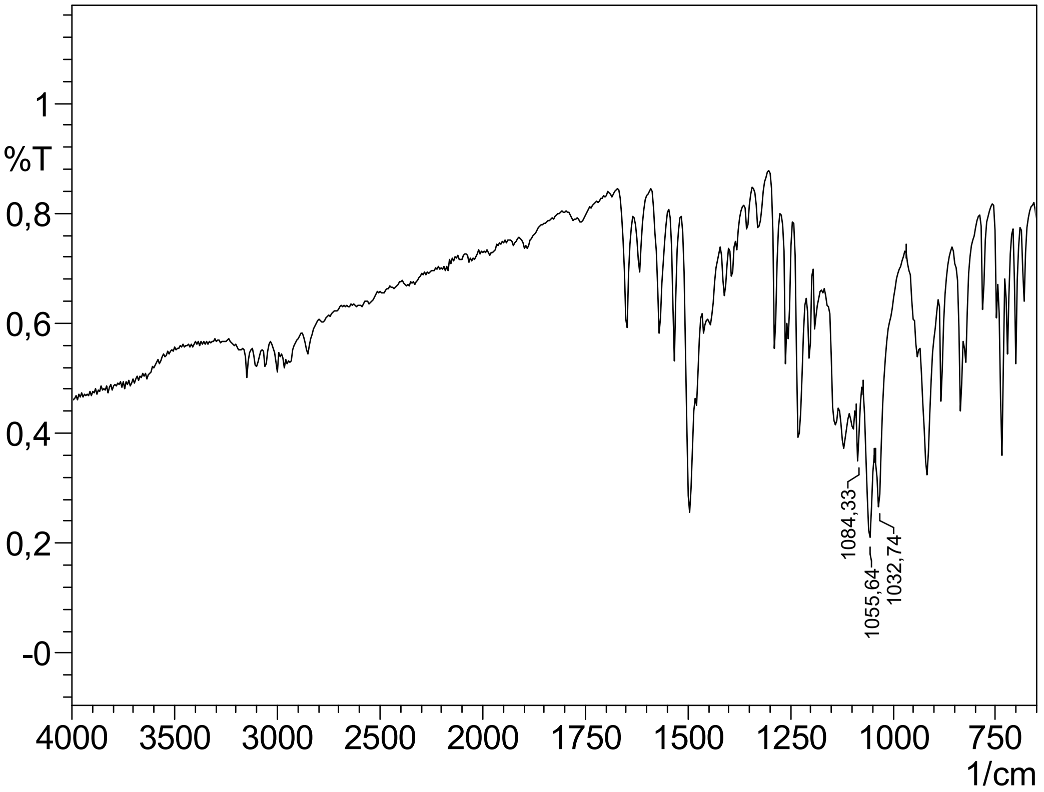

Figure S40. Infrared spectrum (ATR) of compound **^{OMe}IPP_BF$_2$**.

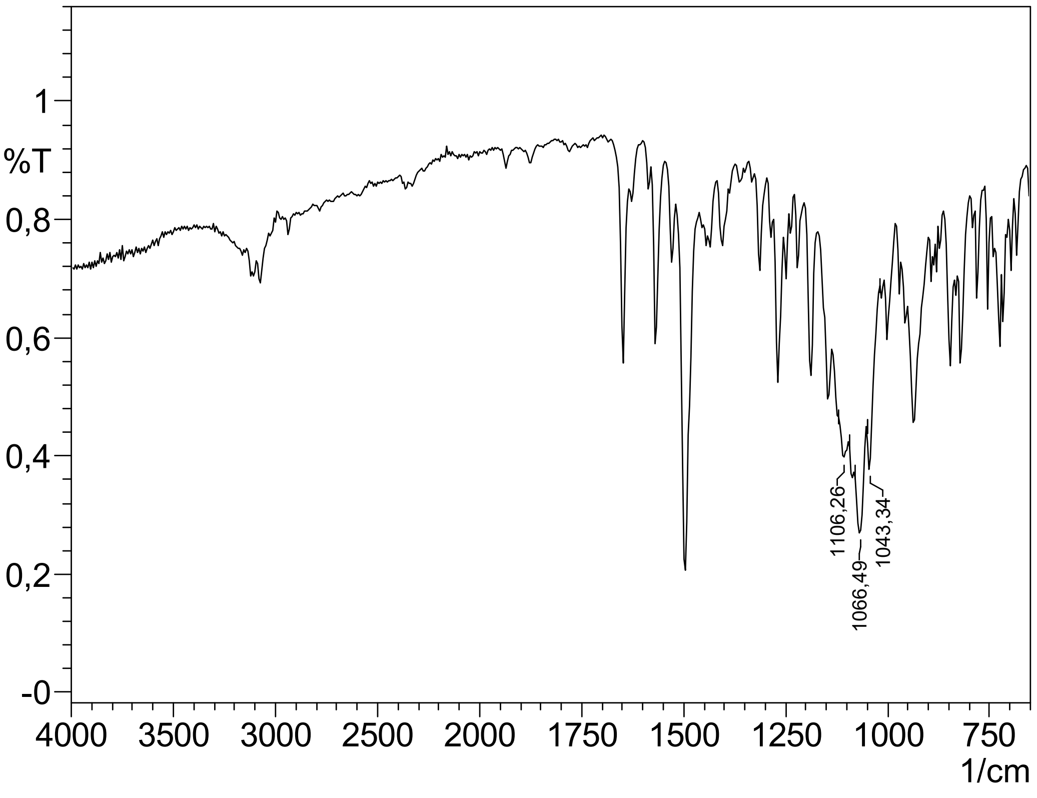

Figure S41. Infrared spectrum (ATR) of compound **^{F}IPP_BF$_2$**.

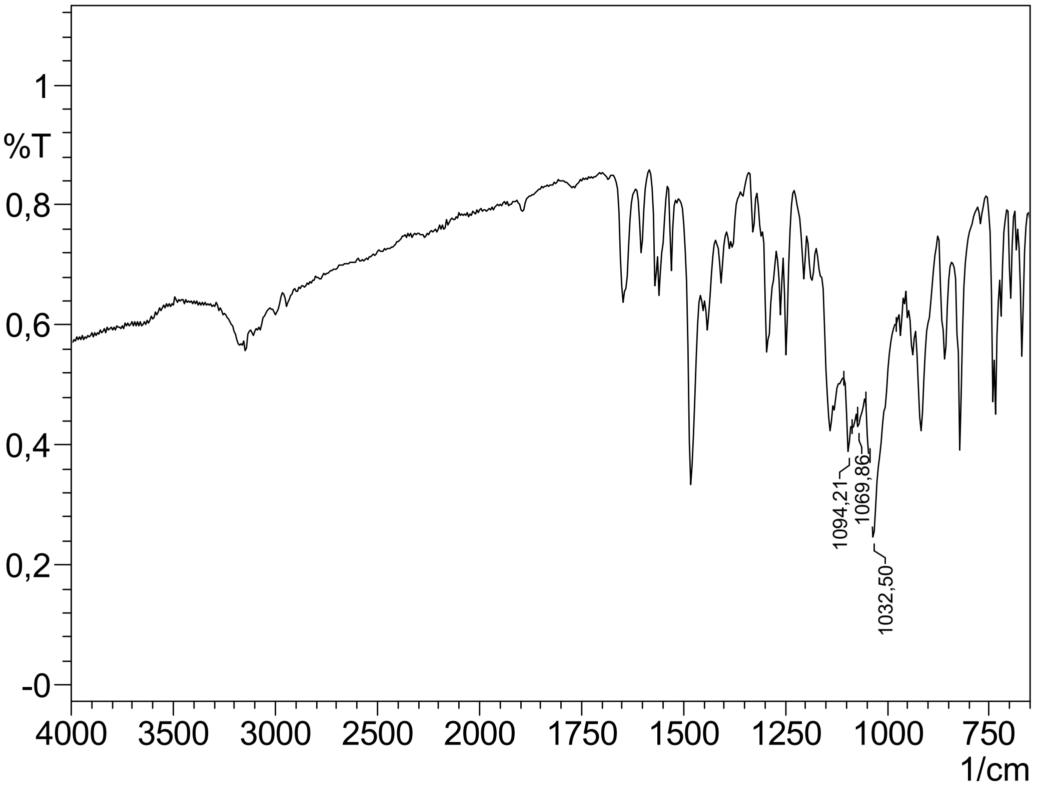

Figure S42. Infrared spectrum (ATR) of compound ^{Br}**IPP_BF$_2$**.

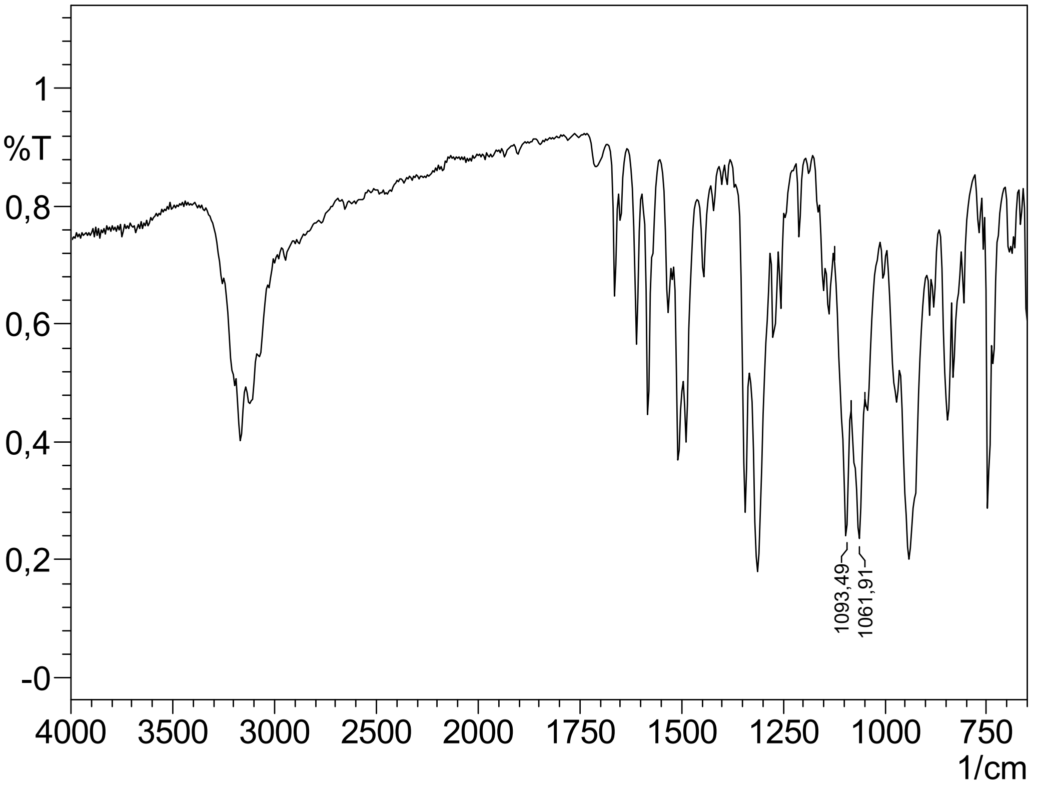

Figure S43. Infrared spectrum (ATR) of compound ^{NO2}**IPP_BF$_2$**.

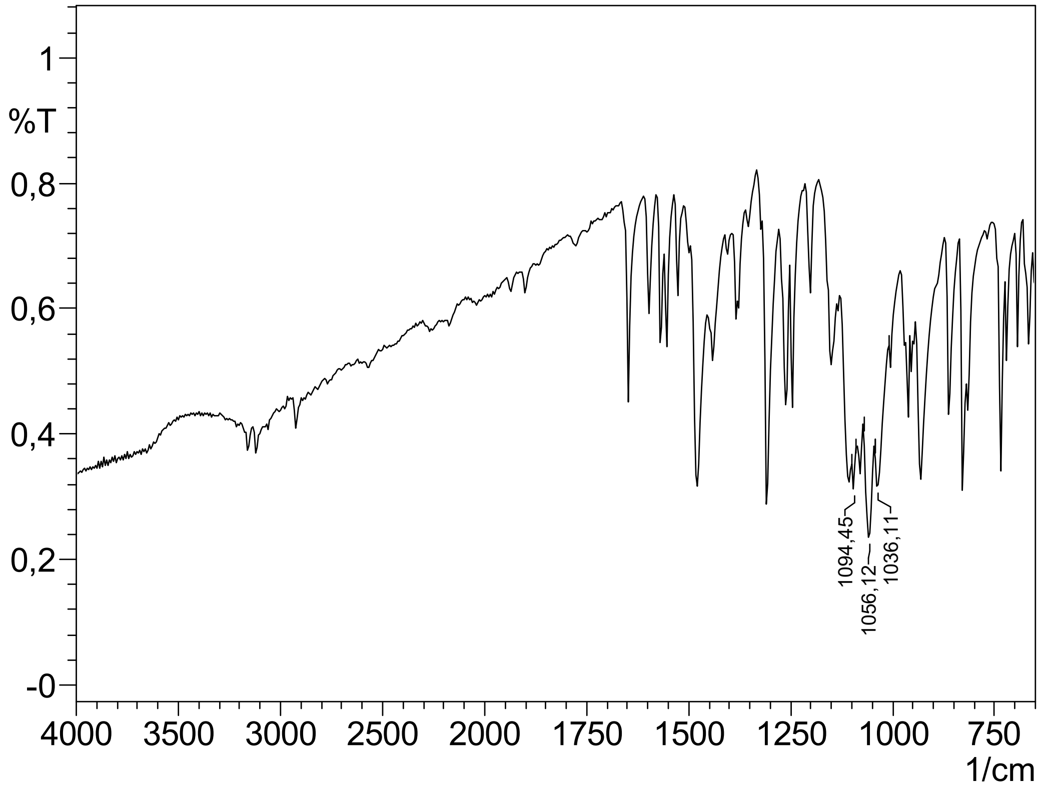

Figure S44. Infrared spectrum (ATR) of compound **^{I}IPP_BF$_2$**.

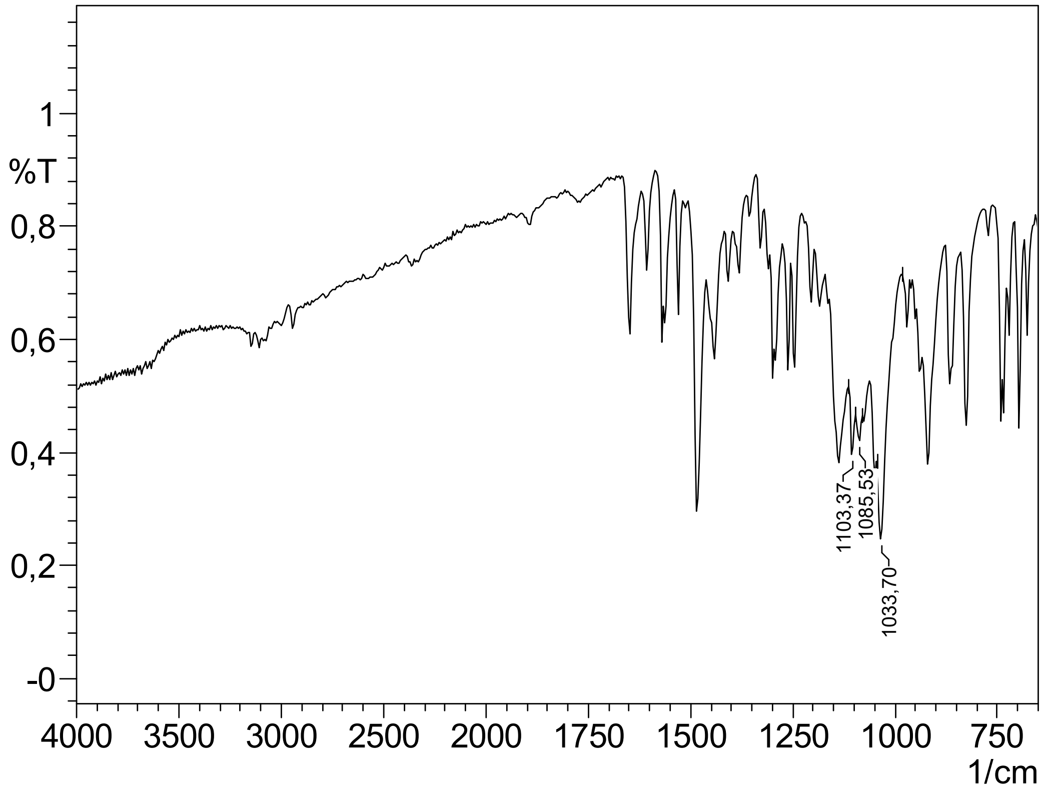

Figure S45. Infrared spectrum (ATR) of compound **^{Cl}IPP_BF$_2$**.

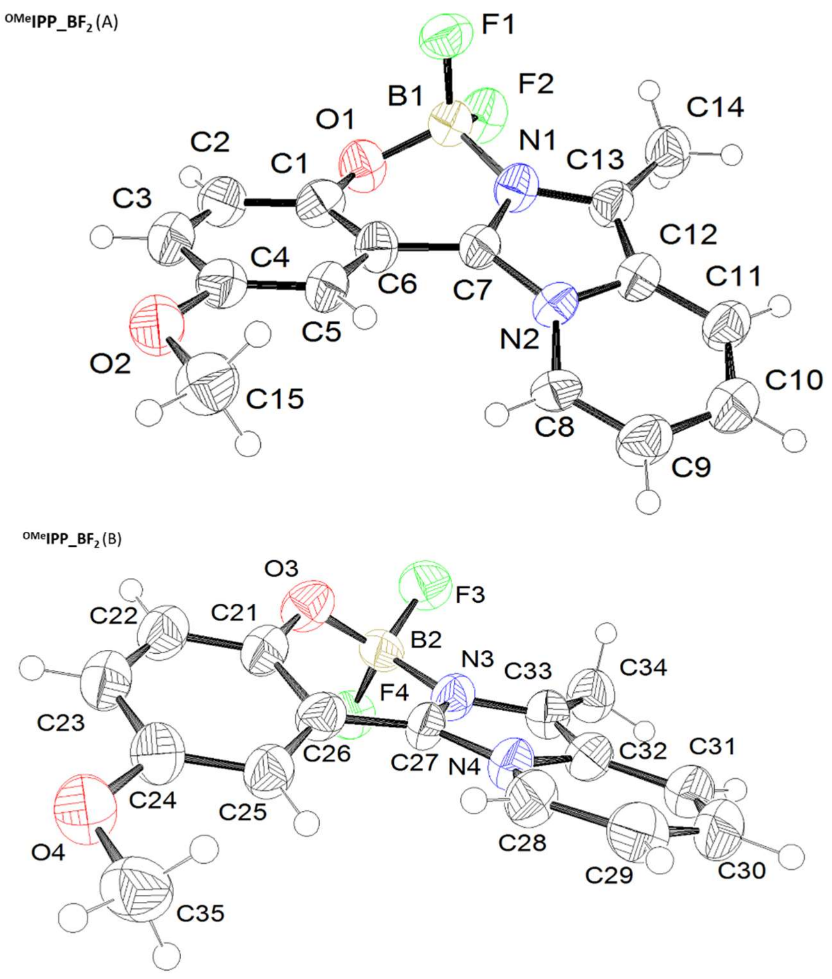

Figure S46. ORTEP representation of ^{OMe}**IPP_BF$_2$** (molecules A and B) at 50% probability level, with atom labeling scheme.

In the checkcif file associated to compound ^{OMe}**IPP_BF$_2$**, one "Alert level A" is present: the reflections differing by more than 10 times sigma (W) are linked to the disorder observed within the structure. In the methoxy derivative, disorder is observed on both, the methoxy and the BF_2 groups, which overall reduces or increases the electron density associated to the corresponding h,k,l planes, thus producing this alert.

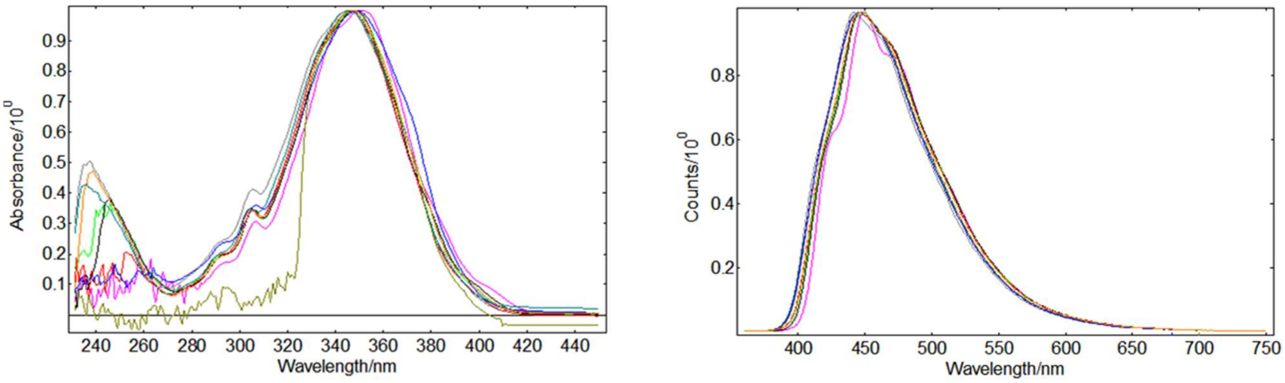

Figure S47. Normalized UV-vis (left) and emission spectra (right) of **^{H}IPP_BF$_2$** measured in various solvents ($5\cdot10^{-5}$ M). Color code: pink (■), toluene; green (■), THF; grey (■), EtOH; black (■), $CHCl_3$; red (■), ethyl acetate; light blue (■), CH_3CN; khaki (■), acetone; blue (■), DMF; orange (■), CH_2Cl_2.

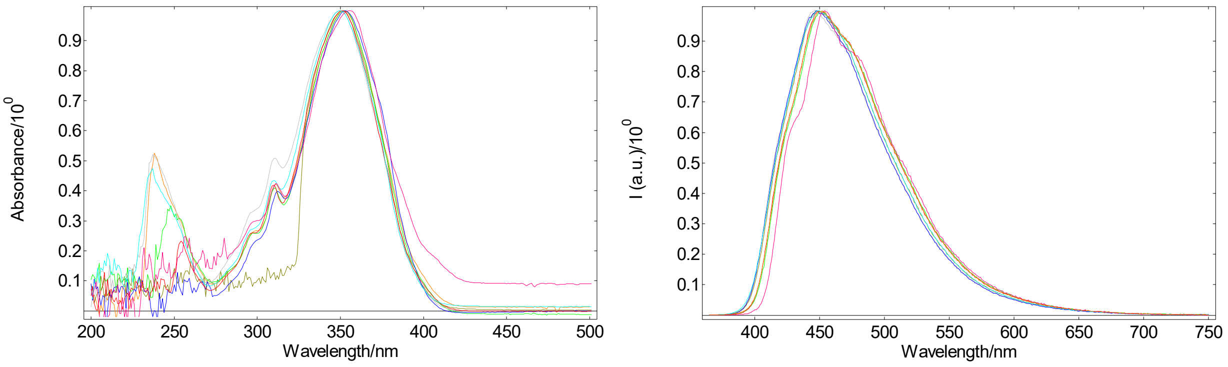

Figure S48. Normalized UV-vis (left) and emission spectra (right) of **^{Me}IPP_BF$_2$** measured in various solvents ($5\cdot10^{-5}$ M). Color code: pink (■), toluene; green (■), THF; grey (■), EtOH; red (■), ethyl acetate; light blue (■), CH_3CN; khaki (■), acetone; blue (■), DMF; orange (■), CH_2Cl_2.

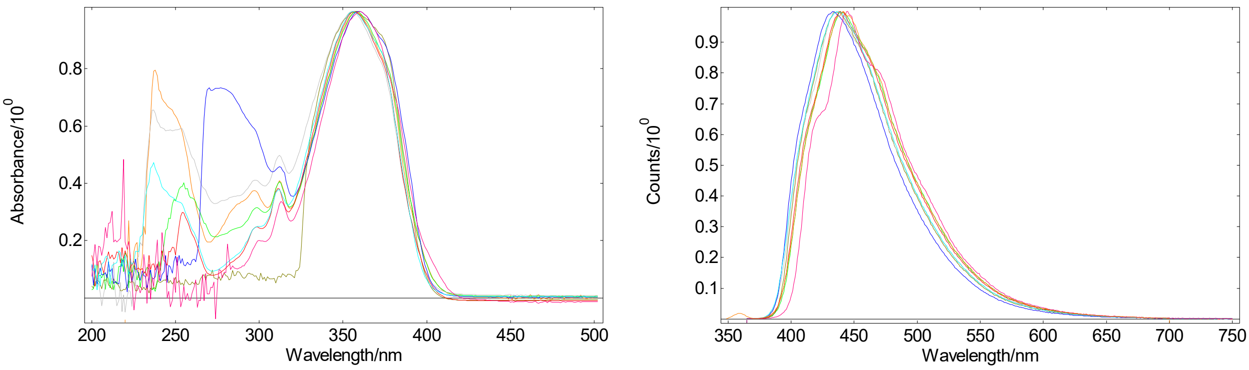

Figure S49. Normalized UV-vis (left) and emission spectra (right) of ^{Cl}**IPP_BF$_2$** measured in various solvents ($5 \cdot 10^{-5}$ M). Color code: pink (■), toluene; green (■), THF; grey (■), EtOH; red (■), ethyl acetate; light blue (■), CH_3CN; khaki (■), acetone; blue (■), DMF; orange (■), CH_2Cl_2.

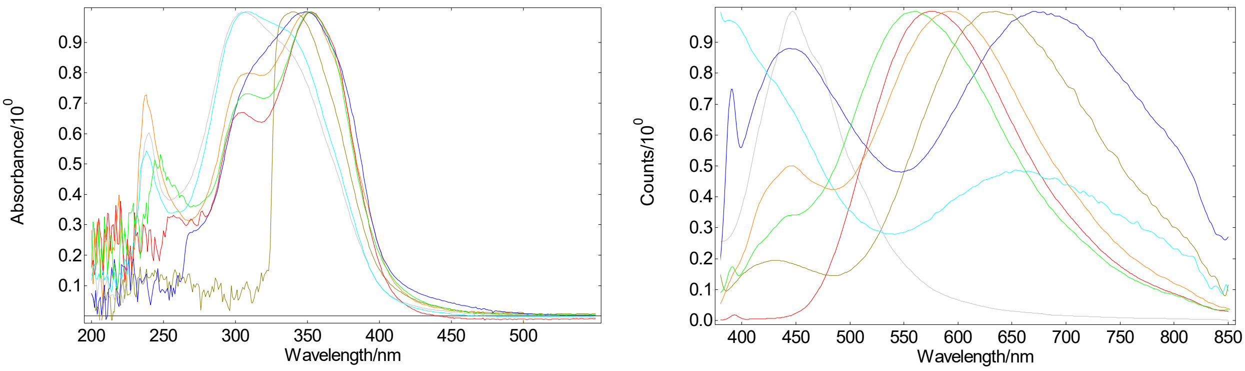

Figure S50. Normalized UV-vis (left) and emission spectra (right) of ^{NO2}**IPP_BF$_2$** measured in various solvents ($5 \cdot 10^{-5}$ M). Color code: green (■), THF; grey (■), EtOH; red (■), ethyl acetate; light blue (■), CH_3CN; khaki (■), acetone; blue (■), DMF; orange (■), CH_2Cl_2.

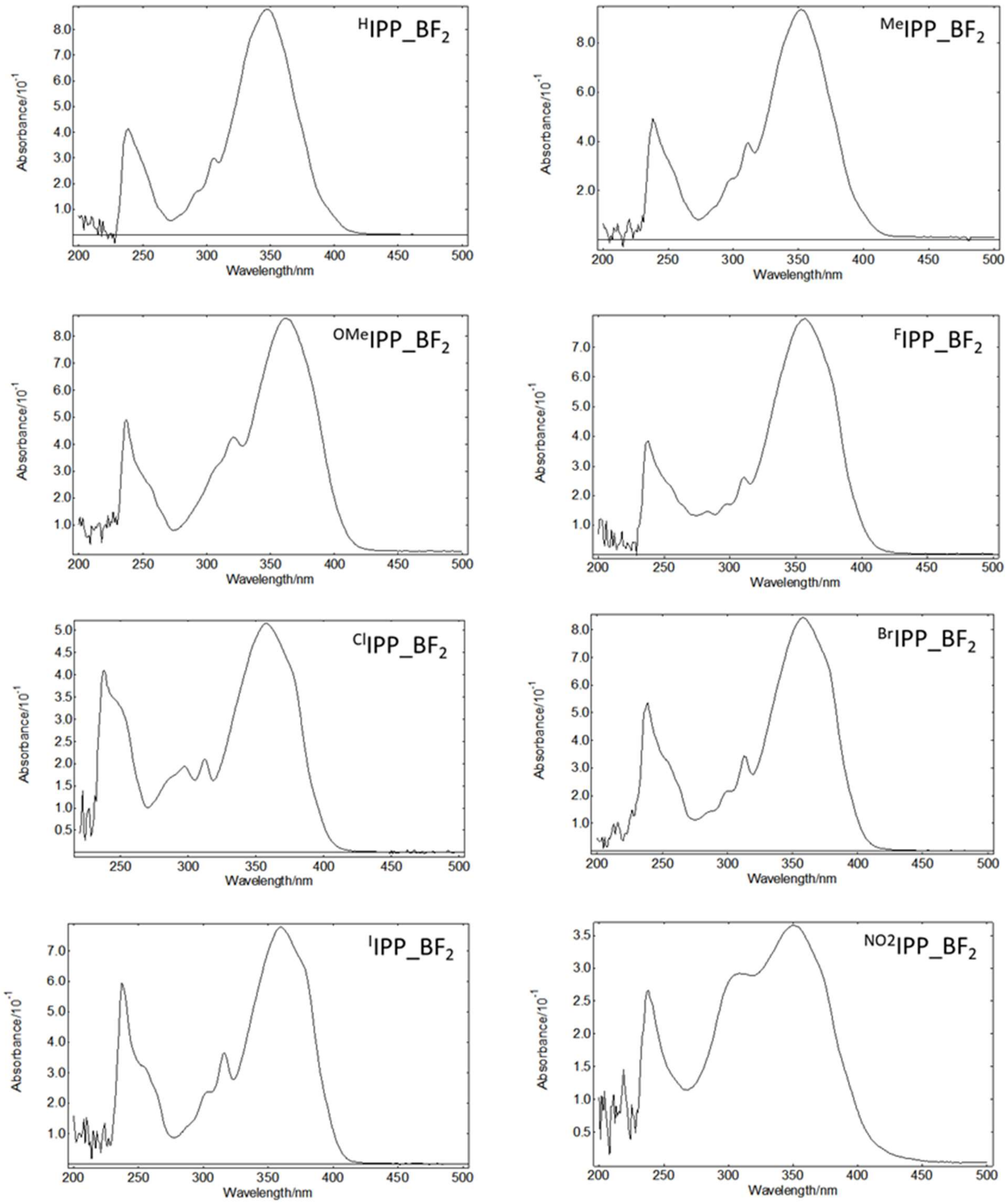

Figure S51. UV-vis spectra of compounds $^{\mathbf{R}}$**IPP_BF$_2$** in CH_2Cl_2 ($5 \cdot 10^{-5}$ M).

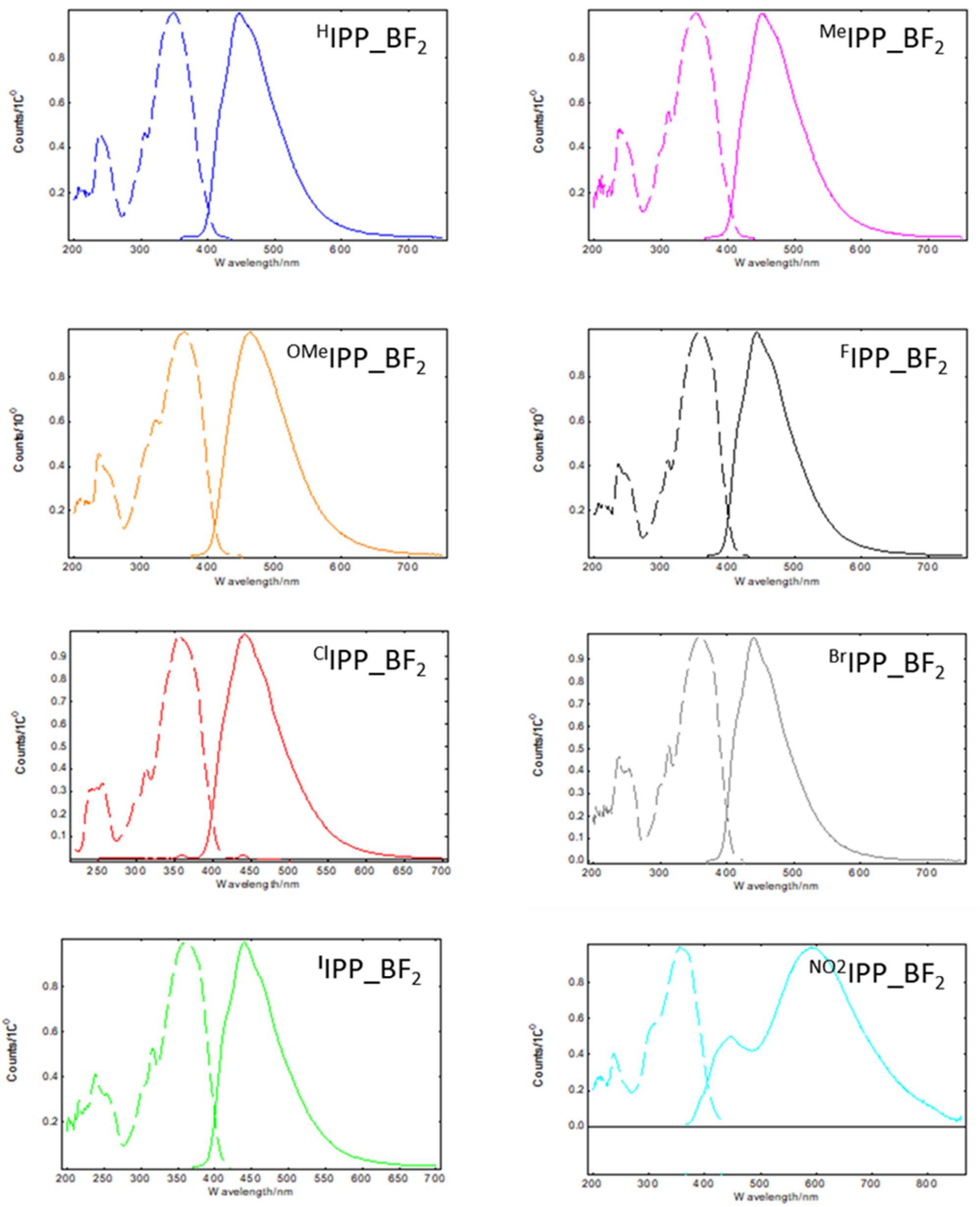

Figure S52. Normalized excitation (dashed) and emission (solid) spectra of **^{R}IPP_BF$_2$** compounds in CH_2Cl_2 ($5\cdot10^{-5}$ M).

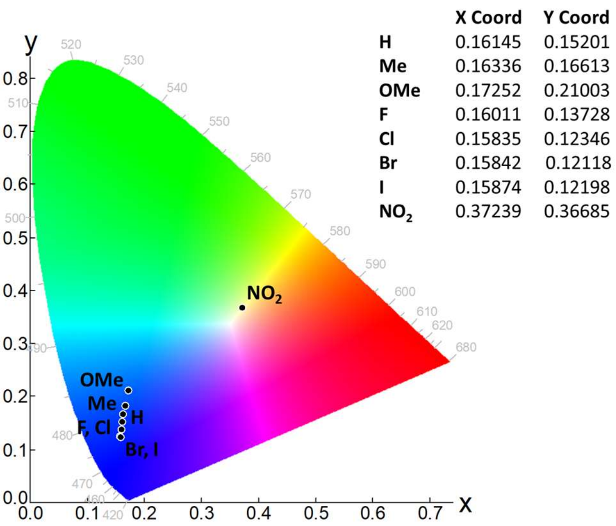

Figure S53. CIE 1931 chromaticity plot for emission of compounds **^{R}IPP_BF$_2$** in dichloromethane solution ($5{\cdot}10^{-5}$ M).

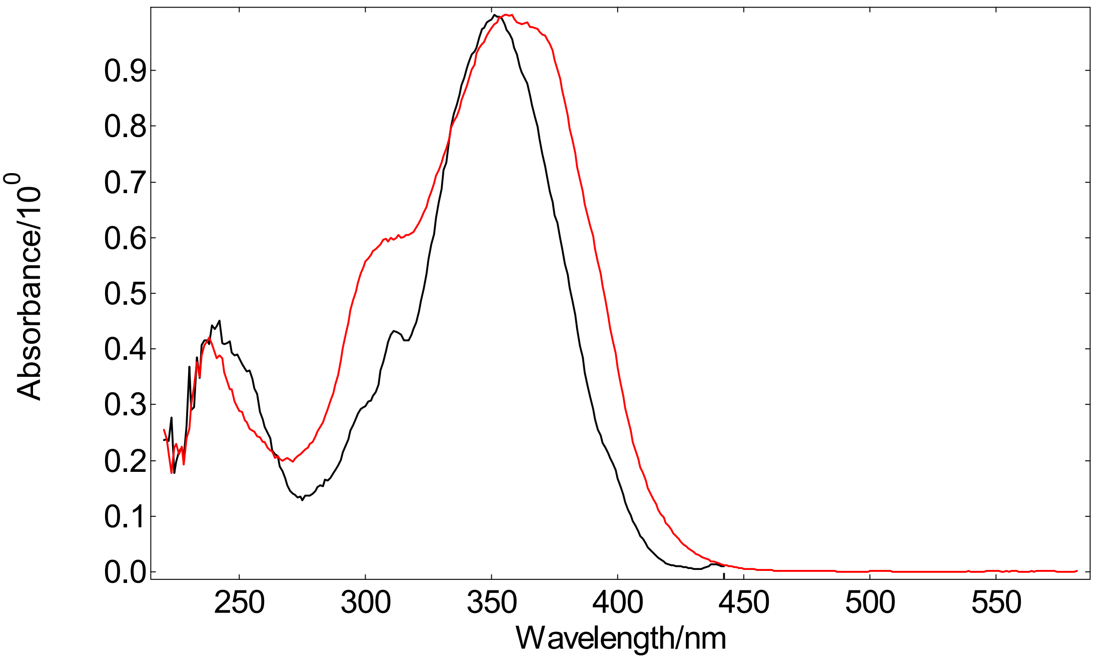

Figure S54. Normalized excitation spectra of **^{NO2}IPP_BF$_2$** in CH_2Cl_2 ($5{\cdot}10^{-5}$ M). Color code: red (■), emission at 597 nm; black (■), emission at 445 nm.

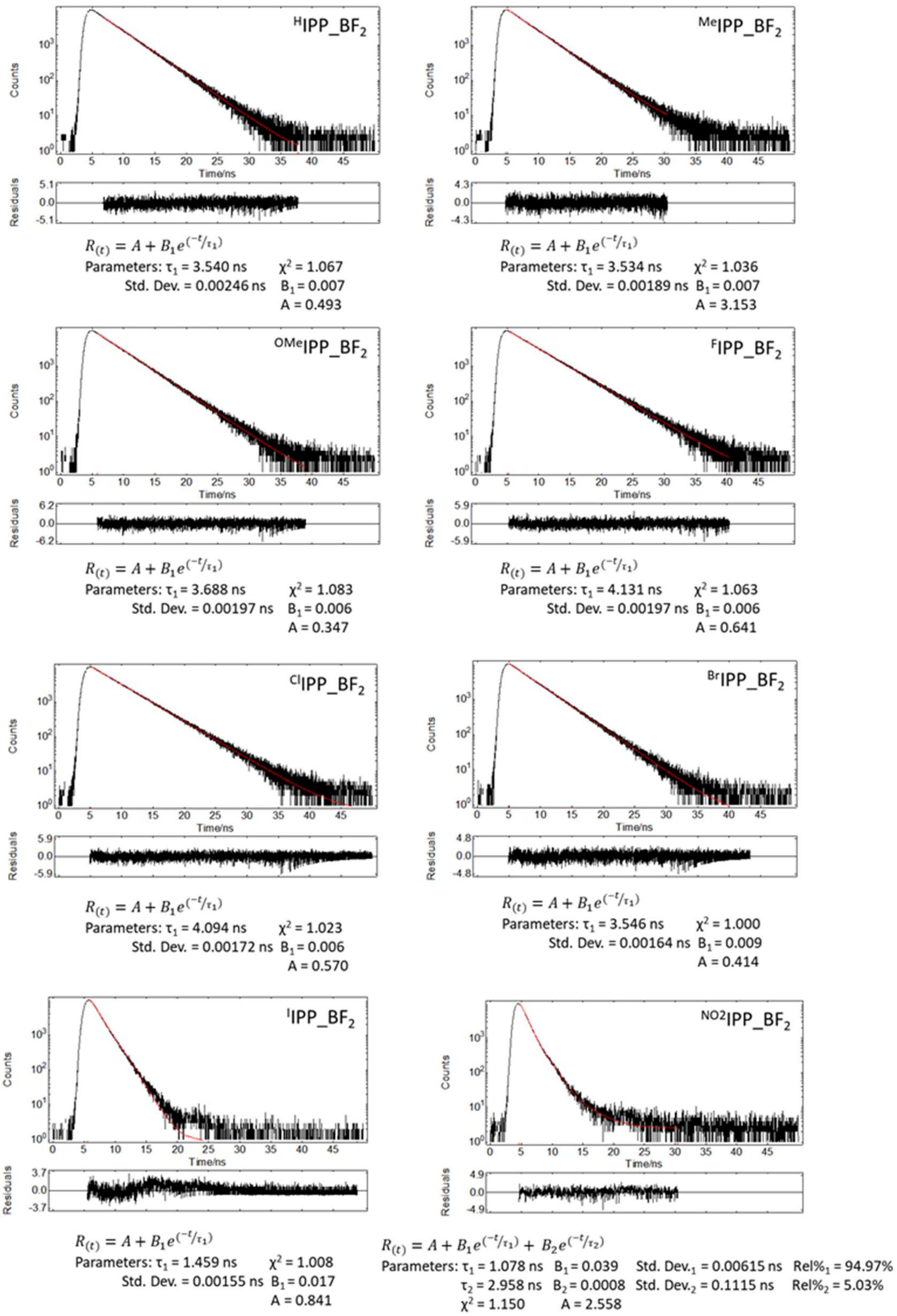

Figure S55. Fitting of the lifetime decay for compounds **^{R}IPP_BF$_2$** in CH_2Cl_2 ($5\cdot10^{-5}$ M).

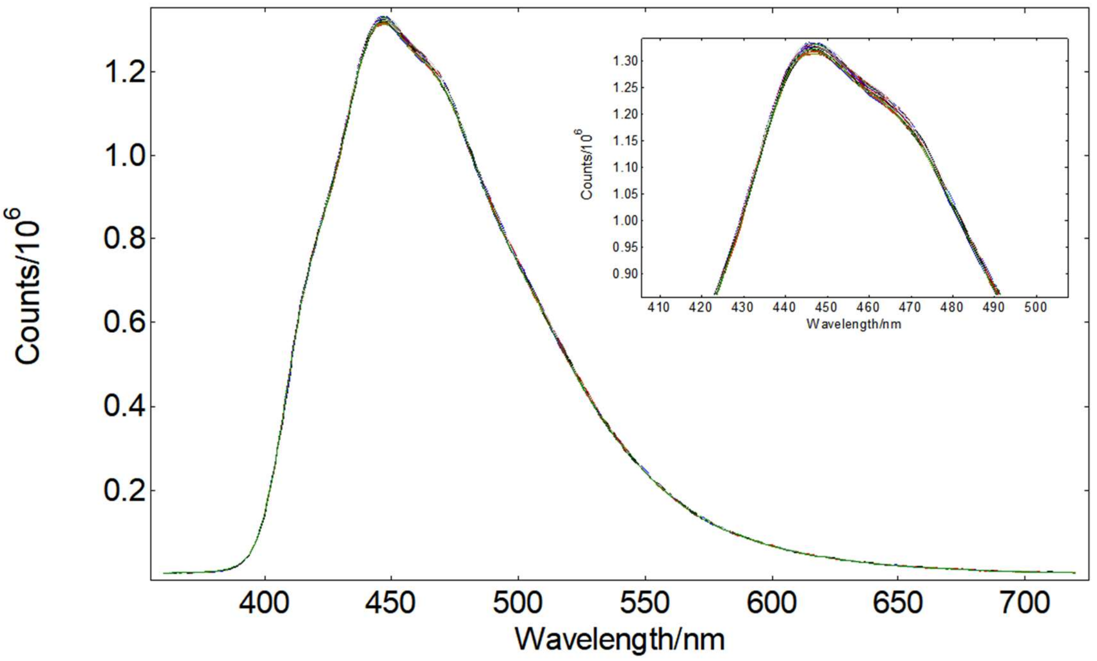

Figure S56. Multiple emission spectra of **^{H}IPP_BF$_2$** in dichloromethane solution ($5 \cdot 10^{-5}$ M): λ_{exc} 348 nm, 100 scans, ca. 1 min/scan, no interval between scans (= total irradiation time ca. 2 hours).

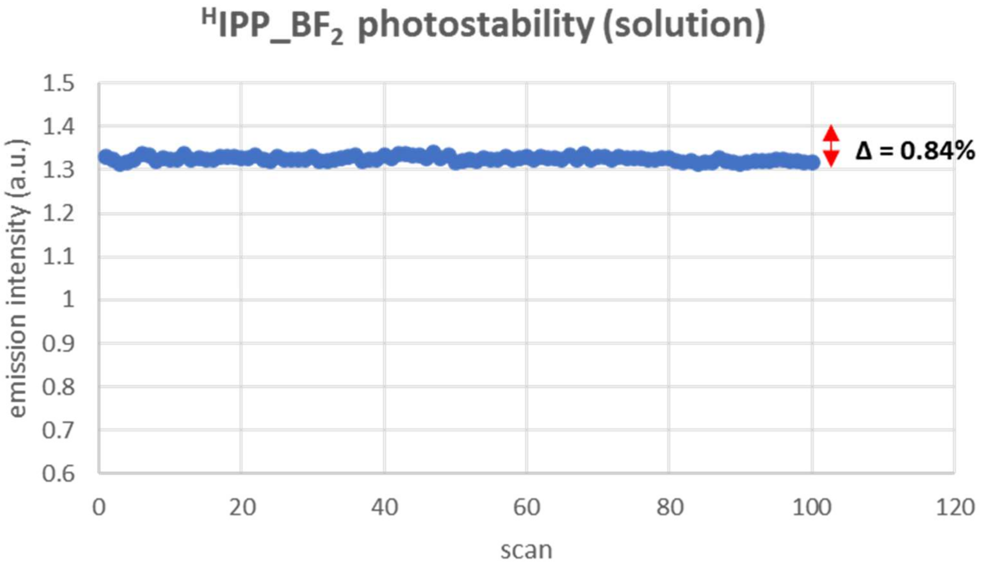

Figure S57. Intensity of emission of **^{H}IPP_BF$_2$** *vs*. time in dichloromethane solution ($5 \cdot 10^{-5}$ M) as obtained by the multiple scan experiment of Figure S42.

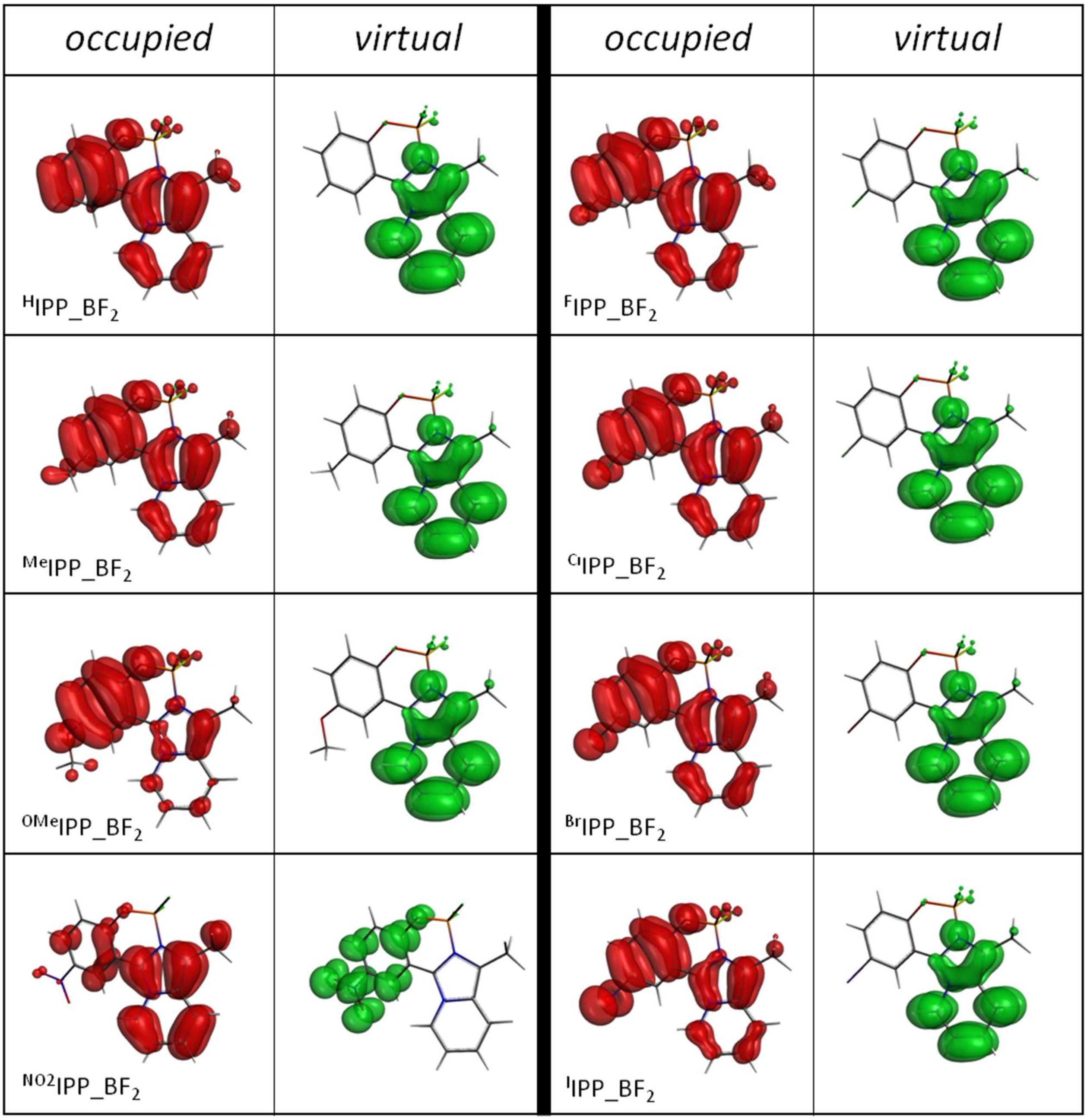

Figure S58. Natural Transition Orbitals (NTOs) calculated for compounds **^{R}IPP_BF$_2$** relative to the first (NTO-1) low energy transition.

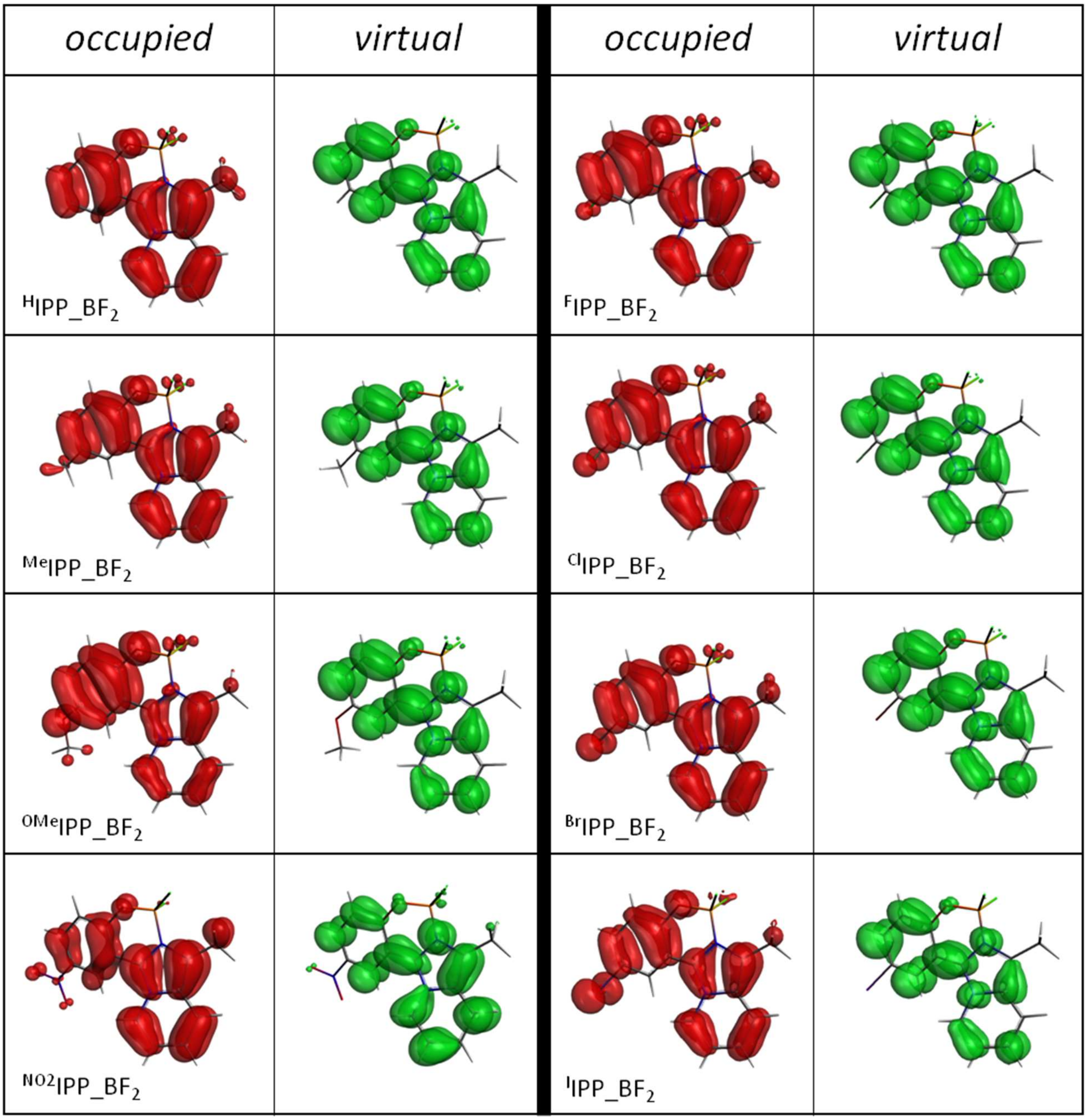

Figure S59. Natural Transition Orbitals (NTOs) calculated for compounds $^{\mathbf{R}}$**IPP_BF$_2$** relative to the second (NTO-2) low energy transition.

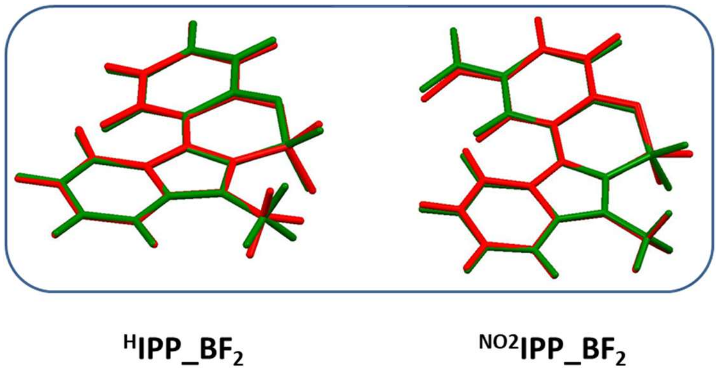

Figure S60. Comparison between ground state (S_0, green) and excited state (S_1, red) calculated optimized geometries for compounds **^{H}IPP_BF$_2$** and **^{NO2}IPP_BF$_2$**.

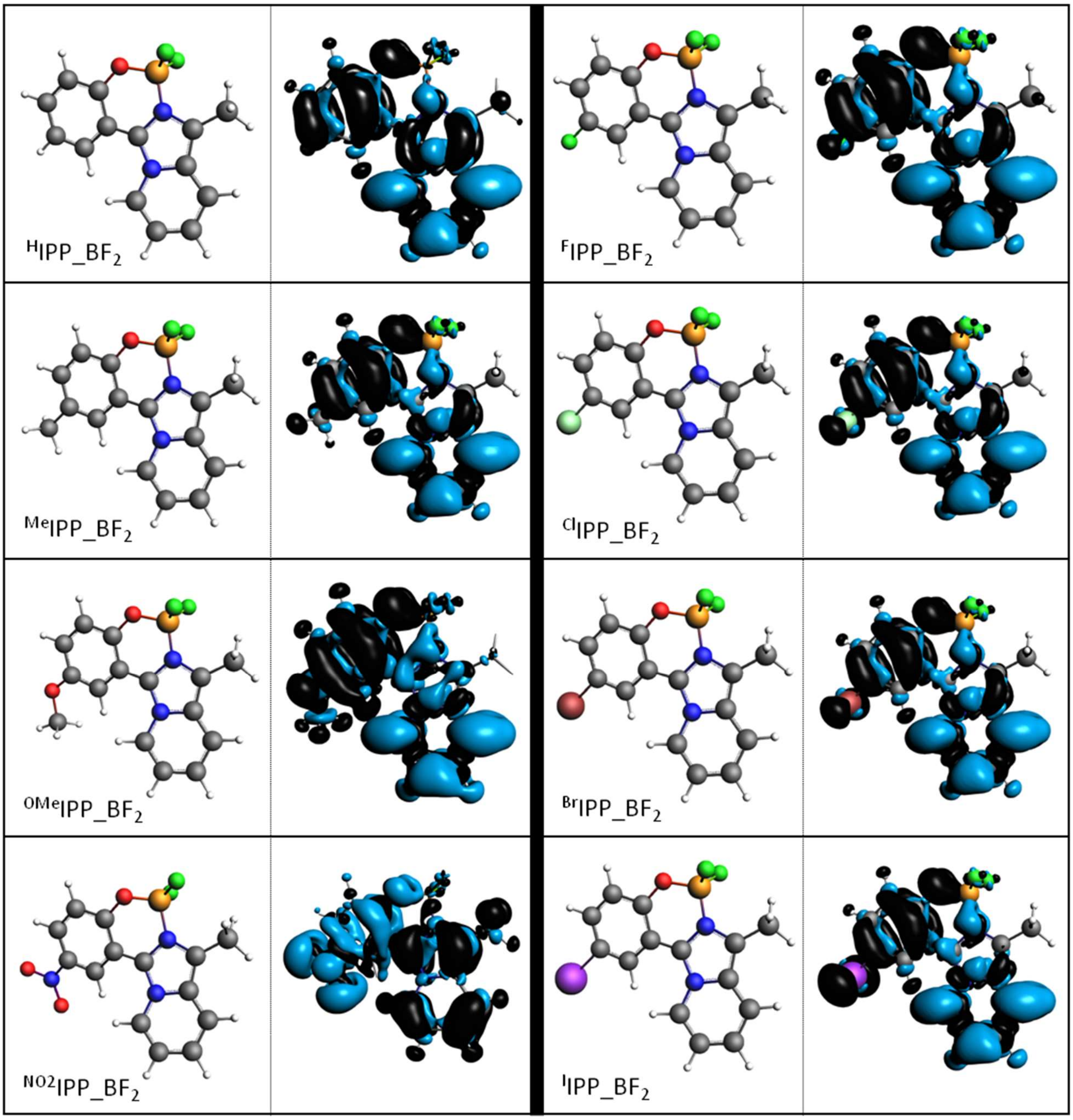

Figure S61. Electron Difference Density Maps (EDDM) for the lowest energy singlet electronic transition computed by TDDFT (**black** indicates a decrease in electron density, **blue** indicates an increase).

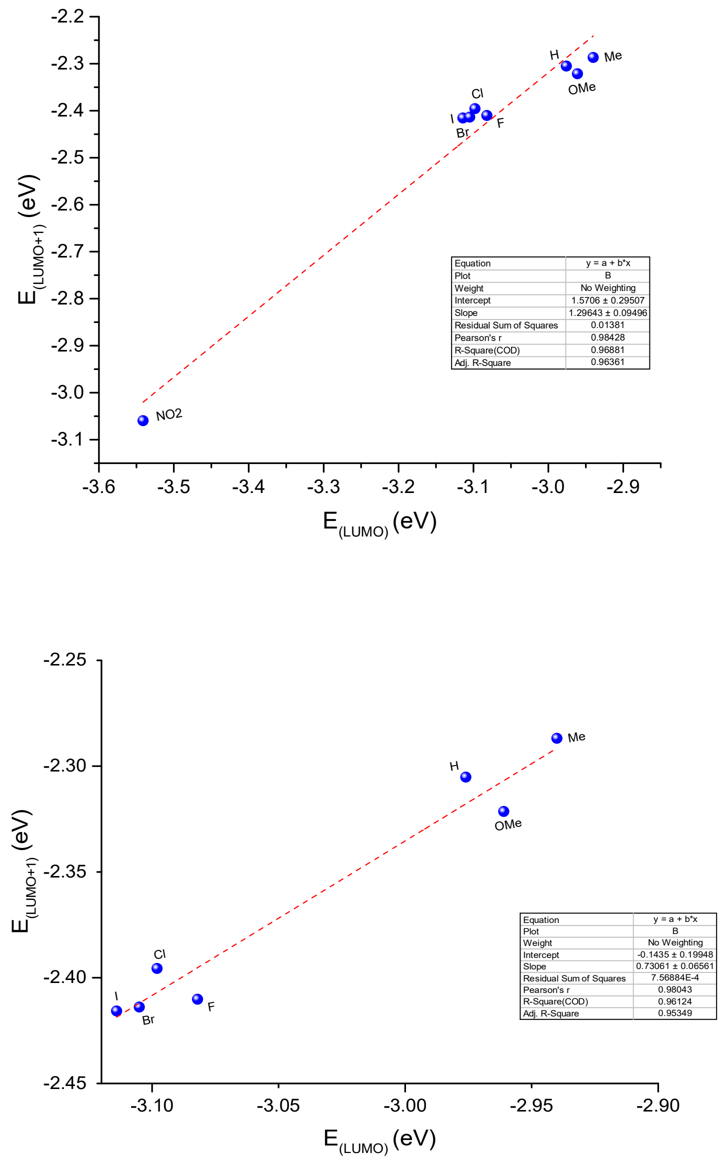

Figure S62. Linear correlation between LUMO and LUMO+1 energies for **^{R}IPP_BF$_2$** (bottom: enlargement of the region between -3.10 and -2.90 eV).

^{I}IPP

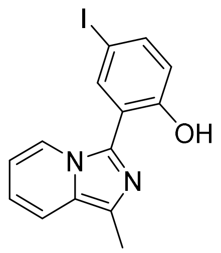

^{1}H NMR (400 MHz, $CDCl_3$, 298 K, *J* [Hz]): δ = 8.34 (d, *J* = 7.2, 1H), 7.97 (d, *J* = 1.7, 1H), 7.51 (dd, *J* = 8.6, 1.7, 1H), 7.44 (d, *J* = 9.0, 1H), 6.91 (d, *J* = 8.6, 1H), 6.74 (m, 1H), 6.78 (t, *J* = 6.6, 1H), 2.54 (s, 3H). ^{13}C NMR (100 MHz, $CDCl_3$, 298 K): δ = 153.1, 137.6, 131.7, 128.2, 127.1, 122.7, 121.5, 120.0, 119.7, 118.3, 118.1, 116.1, 114.8, 114.1, 88.5, 12.1.

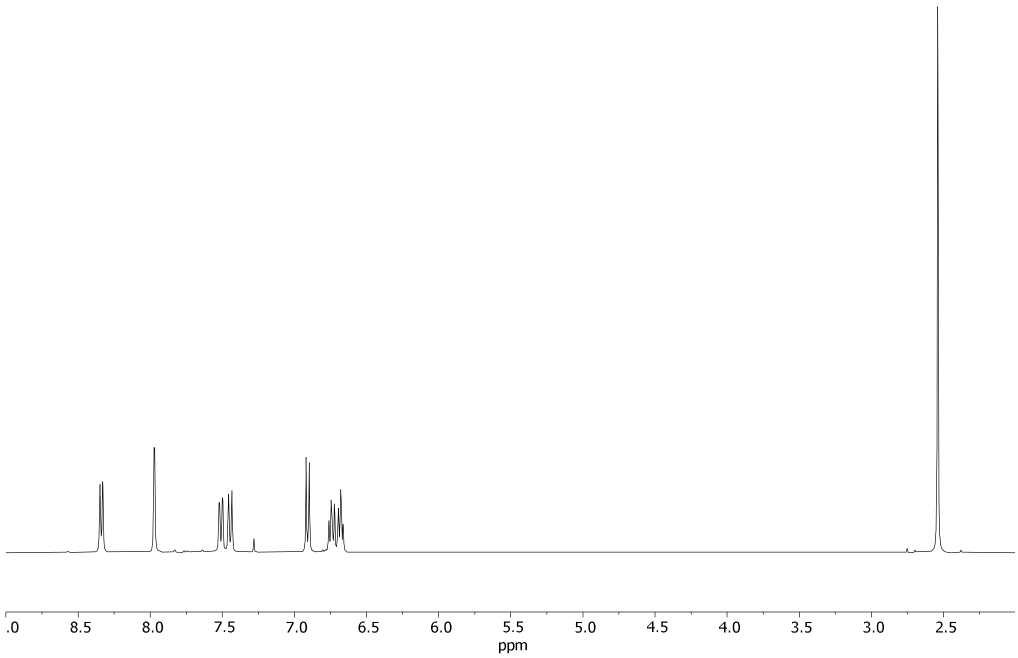

Figure S63. ^{1}H NMR ($CDCl_3$, 25°C) of **^{I}IPP**.

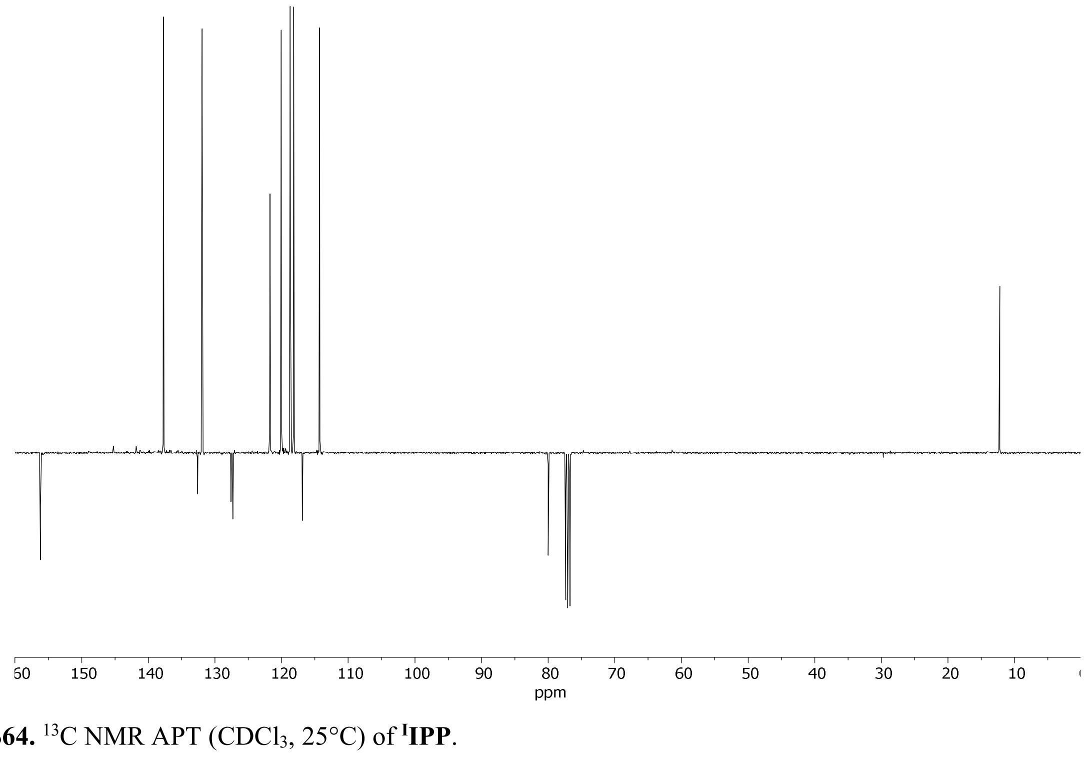

Figure S64. ^{13}C NMR APT ($CDCl_3$, 25°C) of **^{I}IPP**.

^{Cl}IPP

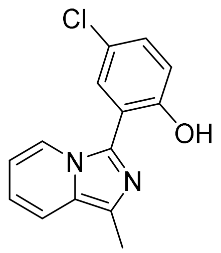

^{1}H NMR (400 MHz, $CDCl_3$, 298 K, *J* [Hz]): δ = 8.38 (d, *J* = 7.2, 1H), 7.68 (d, *J* = 2.3, 1H), 7.46 (d, *J* = 9.0, 1H), 7.21 (dd, *J* = 8.7, 2.3, 1H), 7.07 (d, *J* = 8.7, 1H), 6.75 (m, 1H), 6.68 (t, *J* = 6.6, 1H), 2.55 (s, 3H). ^{13}C NMR (100 MHz, $CDCl_3$, 298 K): δ = 155.0, 133.0, 128.6, 127.6, 127.3, 123.5, 122.7, 121.7, 118.8, 118.5, 118.1, 115.4, 114.3, 114.1, 12.2.

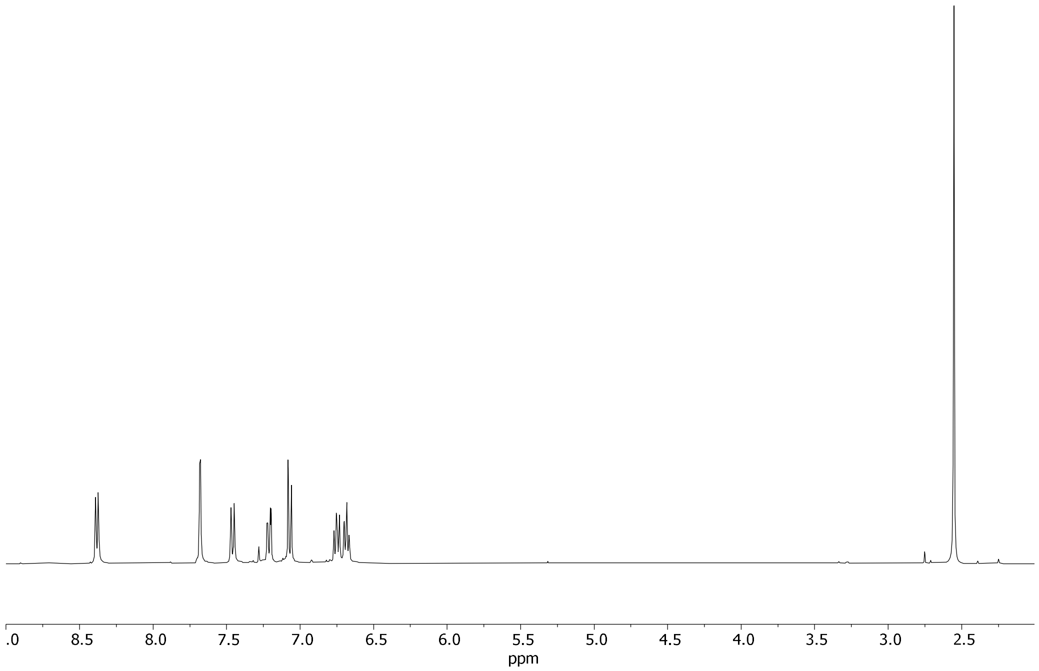

Figure S65. ^{1}H NMR (CD_2Cl_2, 25°C) of **^{Cl}IPP**.

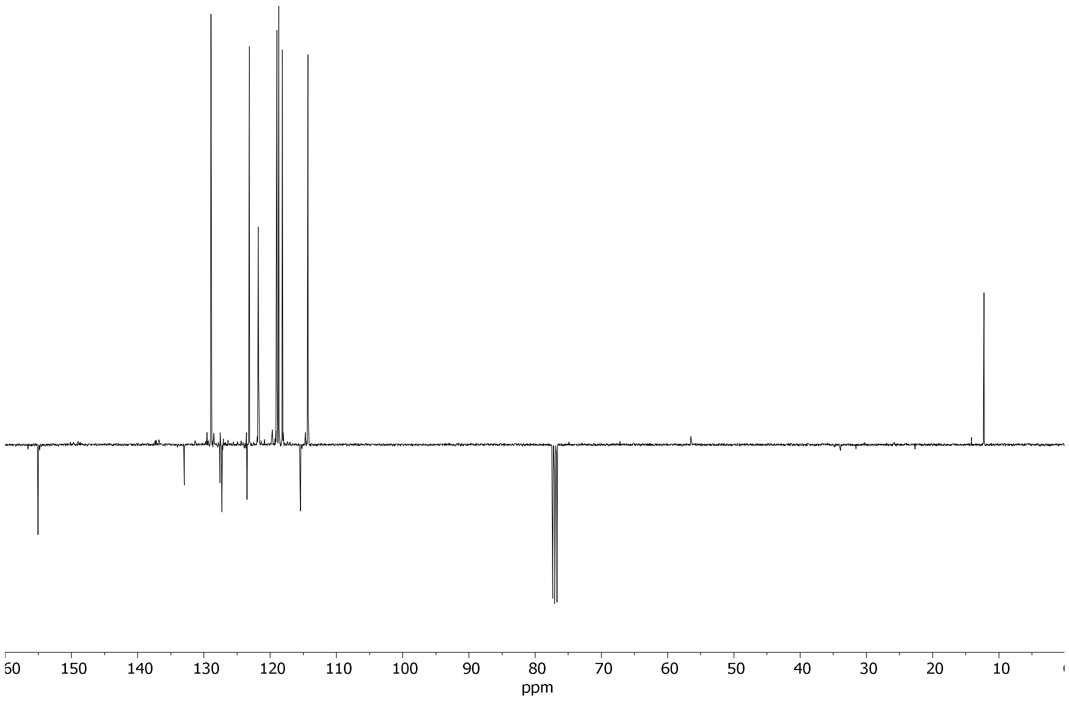

Figure S66. ^{13}C NMR APT (CD_2Cl_2, 25°C) of **^{Cl}IPP**.

Table S1. Crystallographic and structure refinement parameters for compounds **^{H}IPP_BF$_2$** and **^{OMe}IPP_BF$_2$**.

	**^{H}IPP_BF$_2$**	**^{OMe}IPP_BF$_2$**
Chemical formula	$C_{14}H_{11}BF_2N_2O$	$C_{15}H_{13}BF_2N_2O_2$
Formula weight	272.06	302.08
Crystal system	Orthorhombic	Monoclinic
Space group	*P mcn* (no. 62)	$P\,2_1/c$ (no. 14)
Crystal color and shape	Yellow block	Orange block
Crystal size	0.19 x 0.17 x 0.15	0.22 x 0.19 x 0.16
a (Å)	6.8839(5)	17.9831(13)
b (Å)	10.0250(7)	7.1160(4)
c (Å)	17.8687(10)	22.4770(17)
α (°)	90	90
β (°)	90	111.246(6)
γ (°)	90	90
V (Å^3)	1233.14(14)	2680.8(3)
Z	4	8
T (K)	293(2)	293(2)
D_c (g·cm^{-3})	1.465	1.497
μ (mm^{-1})	0.113	0.118
Scan range (°)	$2.30 < \theta < 26.30$	$2.65 < \theta < 67.90$
Unique reflections	1356	4812
Observed refls [I>2σ(I)]	963	2872
R_{int}	0.0343	0.0860
Final R indices [I>2σ(I)]*	0.0649, wR_2 0.1854	0.1248, wR_2 0.2919
R indices (all data)	0.0900, wR_2 0.2072	0.1957, wR_2 0.3871
Goodness-of-fit	1.077	2.473
Max, Min Δρ/e (Å^{-3})	0.353, - 0.463	0.945, - 0.905

* Structures were refined on F_0^2: $wR_2 = [\Sigma[w(F_0^2 - F_c^2)^2] / \Sigma w(F_0^2)^2]^{1/2}$, where $w^{-1} = [\Sigma(F_0^2) + (aP)^2 + bP]$ and $P = [\max(F_0^2, 0) + 2F_c^2]/3$

Table S2. Photophysical data for compound **^{Me}IPP_BF$_2$** recorded in different solvents ($5 \cdot 10^{-5}$ M).

Solvent	λ_{abs}	λ_{exc}	λ_{em}	Stokes shift	ϕ_{PL}	τ
CH_2Cl_2	352	351	452	0.79	0.19	3.5
acetone	351	355	448	0.73	0.19	3.9
CH_3CN	350	349	449	0.79	0.17	4.0
EtOH	349	354	446	0.72	0.15	3.9
THF	353	353	451	0.76	0.20	4.1
toluene	355	355	454	0.76	0.17	3.8
AcOEt	351	352	449	0.77	0.16	3.8
$CHCl_3$	352	353	451	0.77	0.15	3.3
DMF	353	355	448	0.73	0.22	4.4

Table S3. Photophysical data for compound **^{Cl}IPP_BF$_2$** recorded in different solvents ($5 \cdot 10^{-5}$ M).

Solvent	λ_{abs}	λ_{exc}	λ_{em}	Stokes shift	ϕ_{PL}	τ
CH_2Cl_2	358	357	442	0.67	0.23	4.1
acetone	356	359	436	0.61	0.24	4.5
CH_3CN	356	358	436	0.62	0.22	4.5
EtOH	356	357	436	0.63	0.27	4.4
THF	358	358	439	0.64	0.28	4.5
toluene	360	362	444	0.63	0.23	4.4
AcOEt	357	357	440	0.66	0.23	4.3
$CHCl_3$	358	357	441	0.66	0.20	3.8
DMF	359	364	433	0.54	0.35	5.0

Table S4. Photophysical data for compound **^{NO2}IPP_BF$_2$** recorded in different solvents ($5 \cdot 10^{-5}$ M).

Solvent	λ_{abs}	λ_{exc}	λ_{em}	Stokes shift
CH_2Cl_2	350	356	597	0.80
acetone	340	366	623	1.45
CH_3CN	337	354	657	
EtOH	339	395	447	
THF	350	357	560	
toluene	insoluble	insoluble	insoluble	
AcOEt	352	355	577	
DMF	350	676	368	

Coordinates for optimized geometries for ground (S_0) and excited (S_1) states for compounds ^{R}IPP_BF$_2$.

Coordinates for ^{H}IPP_BF$_2$ (ground state, S_0)

C	0.02813665	6.54107320	5.61657620
C	-0.40663059	5.29743747	6.09534511
H	-0.21238337	5.05101276	7.13910115
C	-1.08095622	4.41308982	5.25742430
H	-1.41774601	3.45155882	5.64663673
C	-1.34773286	4.76633291	3.92831114
H	-1.90764509	4.09375326	3.27881888
C	-0.90250068	5.98757967	3.43421268
H	-1.15816718	6.25668927	2.41289669
C	-0.17796155	6.88429792	4.24925602
C	0.37143792	8.14446206	3.81827594
C	1.25856446	10.19757428	4.05556570
C	1.13303480	9.95030755	2.69348204
C	1.43960197	10.69567780	1.53221740
H	1.84325965	11.69986696	1.64978174
O	0.66476603	7.36699195	6.47465773
C	1.23668773	10.13824480	0.29569570
B	0.77085159	8.80821100	6.26182525
H	1.46835499	10.69741134	-0.60923036
C	0.74237754	8.80255931	0.19604549
F	1.96767094	9.26662730	6.84731559
H	0.61686109	8.33069617	-0.77669574
C	0.43016032	8.07734129	1.30708316
F	-0.33609195	9.50022357	6.82084393
H	0.09251915	7.04987695	1.26856828
C	1.77442516	11.41028679	4.74059110
H	2.74494177	11.21414093	5.21677149
H	1.08250017	11.74479678	5.52473969
H	1.90102747	12.22482659	4.01863317
N	0.80088480	9.08090132	4.70628107
N	0.58678489	8.64826524	2.56214923

Coordinates for ^{H}IPP_BF$_2$ (excited state, S_1)

C	-0.07369322	6.57761505	5.65937399
C	-0.51082983	5.32041188	6.12605559
H	-0.38663909	5.09929016	7.18468510
C	-1.09853565	4.41949498	5.25974560
H	-1.44182842	3.45356794	5.62839512
C	-1.28385710	4.75819642	3.89826721
H	-1.79166966	4.06230309	3.23176466
C	-0.83301604	5.96734739	3.40450368
H	-1.02669234	6.21290987	2.36648382
C	-0.17620053	6.90068413	4.24628692
C	0.39238567	8.12902416	3.83612358
C	1.24273683	10.17580512	4.07643952
C	1.11651331	9.92879256	2.68841883
C	1.42787547	10.71535848	1.54899821
H	1.82953270	11.71614197	1.67931776
O	0.44211635	7.41453009	6.54583194
C	1.20915957	10.12743265	0.27097608
B	0.77403955	8.84335280	6.28569011
H	1.42953448	10.70062673	-0.63064357
C	0.73691708	8.83056846	0.16219042
F	2.03005304	9.11397026	6.85570961
H	0.59203438	8.36634040	-0.81293020
C	0.42878012	8.05425143	1.31364288
F	-0.21790900	9.66296646	6.86631025
H	0.20603683	6.99851468	1.28416291
C	1.77649435	11.39211531	4.72348199
H	2.87891234	11.36287429	4.75498041
H	1.41630339	11.49142831	5.75262961
H	1.48918337	12.28438915	4.15238754
N	0.81958127	9.06420420	4.74467954
N	0.60971434	8.64760666	2.55921739

Coordinates for ^{OMe}IPP_BF$_2$ (ground state, S_0)

C	0.02716280	6.49999866	5.74200064
C	-0.38845780	5.26785710	6.26861107
H	-0.14657755	5.03912158	7.30614246
C	-1.09954185	4.36310272	5.49327777
H	-1.42425005	3.40871890	5.90779463
C	-1.43347349	4.68062537	4.16409442
H	-3.14305361	4.93011205	2.05191598
C	-1.01321113	5.88951366	3.61524100
H	-1.32370485	6.15573264	2.61152881
C	-0.24685719	6.79845253	4.38269687
C	0.28309466	8.04072088	3.88448729
C	1.18395294	10.09696763	4.00959817
C	1.00004699	9.80151885	2.66363745
C	1.25418258	10.50408709	1.46320717
H	1.66182505	11.51138859	1.52565289
O	0.71340196	7.34845546	6.54515089
C	0.99425835	9.90442630	0.25747941
B	0.77587673	8.78239203	6.28714465
H	1.18195250	10.43207401	-0.67585146
C	0.49995198	8.56503805	0.22708133
F	1.97594861	9.28871260	6.82350835
H	0.33379526	8.05820980	-0.72140255
C	0.23973885	7.88018925	1.37620012
F	-0.33253019	9.46893599	6.85214757
H	-0.08981142	6.84942550	1.39003479
C	1.73042234	11.33329859	4.62577494
H	2.71779534	11.15087354	5.07155355
H	1.07101876	11.70232305	5.42236680
H	1.83193543	12.11778834	3.86775523
N	0.75279277	9.00589934	4.72014062
N	0.44633534	8.49704946	2.60253027
O	-2.17879047	3.74355495	3.49274032
C	-2.51995615	4.02577241	2.12841311
H	-1.61685199	4.14069903	1.50882514
H	-3.09216155	3.16044573	1.78019687

Coordinates for ^{OMe}IPP_BF$_2$ (excited state, S_1)

C	-0.09554507	6.53204404	5.78387224
C	-0.56307536	5.30662063	6.32486845
H	-0.39473853	5.12295086	7.38373450
C	-1.21828943	4.39338074	5.53795906
H	-1.58570179	3.44946675	5.93503444
C	-1.44549686	4.69494887	4.16831179
H	-3.02820332	4.88010933	1.95306812
C	-0.96835414	5.89176132	3.59939571
H	-1.19093983	6.11252685	2.56286426
C	-0.25720524	6.80876624	4.37014260
C	0.30669955	8.03544323	3.88392646
C	1.15779386	10.09349744	4.02081114
C	0.95863568	9.80665637	2.66077939
C	1.18609039	10.54951699	1.47206996
H	1.59055862	11.55612354	1.54176984
O	0.47847180	7.38082533	6.61180930
C	0.91173528	9.91796679	0.22747158
B	0.88516383	8.79024307	6.27477620
H	1.08148549	10.46056749	-0.70367796
C	0.45480270	8.60801569	0.19118806
F	2.21595508	8.96391094	6.69129899
H	0.28654733	8.10240797	-0.76061664
C	0.19158351	7.87021088	1.37112552
F	0.03008703	9.66823109	6.96960030
H	0.08362640	6.79386065	1.38246184
C	1.72542631	11.32183099	4.63215719
H	2.79992471	11.20641841	4.84587627
H	1.22446312	11.56235721	5.57850157
H	1.60685926	12.17365474	3.95185301
N	0.76642129	8.99223452	4.74210877
N	0.44597702	8.50404333	2.60391174
O	-2.13387254	3.76627956	3.48959734
C	-2.41806280	3.97877124	2.08556321
H	-1.48321203	4.05122183	1.51695035
H	-2.97935139	3.09661671	1.77111192

Coordinates for ^{NO2}IPP_BF$_2$ (ground state, S_0)

C	-0.07567066	6.58493519	5.64302522
C	-0.53626099	5.34574601	6.12726238
H	-0.37171414	5.11192026	7.17769804
C	-1.19117487	4.45561339	5.29553354
H	-1.55162599	3.49889550	5.66536462
C	-1.40046448	4.81363491	3.95707895
O	-2.52173816	2.82747406	3.54181666
C	-0.93743995	6.02160418	3.44065079
H	-1.17016776	6.24706964	2.40669649
C	-0.23509071	6.91449104	4.25843333
C	0.34294711	8.15852593	3.82160872
C	1.28878726	10.18363739	4.05187802
C	1.14654719	9.93774127	2.68928693
C	1.46594698	10.67623259	1.52838323
H	1.90223740	11.66646190	1.64446068
O	0.52399136	7.40481605	6.50517384
C	1.22992438	10.12941025	0.29256190
B	0.77910624	8.83666241	6.26283243
H	1.46661063	10.68426507	-0.61329215
C	0.68874867	8.81382861	0.19534067
F	2.02468126	9.16812492	6.82000082
H	0.52918955	8.35188680	-0.77676803
C	0.36773745	8.09418123	1.30797960
F	-0.24519022	9.62372515	6.83388391
H	-0.00764209	7.08083696	1.26425348
C	1.84647038	11.38003338	4.73222419
H	2.81009624	11.15052780	5.20723105
H	1.16674979	11.74198854	5.51468474
H	2.00162401	12.18666304	4.00758603
N	0.80718485	9.08178409	4.70601459
N	0.56196319	8.65388510	2.56276887
O	-2.31123599	4.25611999	1.89559822
N	-2.12274121	3.91170207	3.07832913

Coordinates for ^{NO2}IPP_BF$_2$ (excited state, S_1)

C	0.09513253	6.53880642	5.62014030
C	-0.35512723	5.29929626	6.07708439
H	-0.11597867	5.00149978	7.09764167
C	-1.11490410	4.46829414	5.25801908
H	-1.47837247	3.50891013	5.62002471
C	-1.45290263	4.87717605	3.95669471
O	-2.67996668	2.92861446	3.58575619
C	-0.98301731	6.08845696	3.46296696
H	-1.30488010	6.39081300	2.47070591
C	-0.17957605	6.90890409	4.27665339
C	0.36817046	8.16252804	3.83685362
C	1.25141015	10.20017266	4.05387255
C	1.13514624	9.93331002	2.67065389
C	1.45436513	10.65998680	1.52603407
H	1.85326735	11.66542677	1.62662335
O	0.81580330	7.34433229	6.44805746
C	1.26184508	10.07079884	0.28195331
B	0.71288308	8.78140892	6.31442931
H	1.50350407	10.62000793	-0.62419505
C	0.78171843	8.74985594	0.19556696
F	1.79731238	9.39702328	6.94436017
H	0.66346703	8.26227200	-0.76763051
C	0.47194864	8.03791823	1.33144049
F	-0.51643314	9.29997760	6.77097239
H	0.15358688	7.00419149	1.31668372
C	1.78962824	11.40137008	4.70676428
H	2.75498385	11.16544444	5.17896818
H	1.11914057	11.74295297	5.50544674
H	1.93717123	12.20391387	3.97911683
N	0.78447777	9.09755579	4.71734150
N	0.60743460	8.64359808	2.55405341
O	-2.57248900	4.47387369	1.93009430
N	-2.28636288	4.06573364	3.12243261

Coordinates for ^{F}IPP_BF$_2$ (ground state, S_0)

C	0.03023780	6.53794843	5.61227636
C	-0.41475307	5.29729216	6.08900504
H	-0.21801008	5.04416181	7.13038969
C	-1.10236836	4.41129504	5.26113752
H	-1.45327249	3.45021267	5.63428921
C	-1.35246051	4.79196032	3.94524404
F	-2.07342687	3.94665832	3.12940522
C	-0.91046441	5.99590788	3.42510298
H	-1.18008783	6.24580514	2.40362162
C	-0.17775186	6.88296254	4.24636598
C	0.37625420	8.14235538	3.81808761
C	1.25353213	10.19908398	4.05791354
C	1.12970334	9.95142989	2.69474401
C	1.43303410	10.69783085	1.53373174
H	1.82845650	11.70541874	1.65021987
O	0.68073834	7.35696846	6.46807337
C	1.23573152	10.13688333	0.29756446
B	0.79063623	8.80007160	6.26327997
H	1.46367661	10.69684052	-0.60770382
C	0.75335891	8.79727167	0.19855968
F	1.99713973	9.25086032	6.83411421
H	0.63582789	8.32191420	-0.77325647
C	0.44320326	8.07189964	1.31020626
F	-0.30490043	9.49556766	6.83857188
H	0.11810723	7.04031835	1.27407126
C	1.76228020	11.41749132	4.73921693
H	2.74220440	11.23545148	5.20169753
H	1.07658762	11.74199827	5.53285664
H	1.86790768	12.23484201	4.01686588
N	0.80375979	9.07928871	4.70766796
N	0.59057972	8.64758213	2.56302380

Coordinates for ^{F}IPP_BF$_2$ (excited state, S_1)

C	-0.10018763	6.57535606	5.65623725
C	-0.59104335	5.33505864	6.12959384
H	-0.49820994	5.12666106	7.19352551
C	-1.18179174	4.43144644	5.27115868
H	-1.56718161	3.47671035	5.62194832
C	-1.29742524	4.78261054	3.91175231
F	-1.92504681	3.91461450	3.07420549
C	-0.81002421	5.96677061	3.38928185
H	-0.98008837	6.17308412	2.33931710
C	-0.16392531	6.88954776	4.23724306
C	0.41578460	8.12053225	3.83500650
C	1.22062577	10.18614211	4.08059825
C	1.10440533	9.93562067	2.69450964
C	1.39465346	10.72740641	1.55400475
H	1.76433586	11.74104545	1.68293456
O	0.41497293	7.40284142	6.54467770
C	1.21082923	10.12676147	0.27590332
B	0.85920484	8.81182370	6.28213271
H	1.42658564	10.70079346	-0.62627636
C	0.77953824	8.81512137	0.16978621
F	2.17373883	8.94974284	6.76065485
H	0.66764869	8.33874309	-0.80407905
C	0.47161541	8.03728301	1.31815682
F	-0.00935526	9.68653011	6.96085018
H	0.30817164	6.96988834	1.29171053
C	1.72042280	11.42485554	4.71800313
H	2.80093144	11.54741993	4.53712751
H	1.55116269	11.42313061	5.79882660
H	1.22119141	12.30380568	4.28365964
N	0.81461630	9.06536107	4.74789766
N	0.62930564	8.63886413	2.56599543

Coordinates for ^{Me}IPP_BF$_2$ (ground state, S_0)

C	-0.01227019	6.57457267	5.63093481
C	-0.46702108	5.33661825	6.10505838
H	-0.26923827	5.07811345	7.14537646
C	-1.16272897	4.46934826	5.26992195
H	-1.51007691	3.51247667	5.66462040
C	-1.44905568	4.81378183	3.93563617
H	-1.74375094	2.91461016	2.94307415
C	-0.97787347	6.03605720	3.46034524
H	-1.23837021	6.31884312	2.44321371
C	-0.22879443	6.92029385	4.26922982
C	0.34208851	8.16809512	3.83060910
C	1.28776876	10.19697408	4.05694339
C	1.14282354	9.95197664	2.69668801
C	1.46106249	10.68641671	1.53140738
H	1.89468477	11.67863799	1.64377232
O	0.65254193	7.38495397	6.48522720
C	1.23085177	10.13380561	0.29757552
B	0.78696621	8.82201783	6.26911196
H	1.47040910	10.68504568	-0.61012098
C	0.69616246	8.81314940	0.20423221
F	1.99919968	9.25686694	6.84243978
H	0.54682860	8.34435254	-0.76658402
C	0.37399289	8.09853789	1.31913658
F	-0.29961459	9.54017719	6.83824629
H	0.00634556	7.08143775	1.28575041
C	1.84346554	11.39618097	4.73495936
H	2.81166643	11.17396514	5.20437014
H	1.16775481	11.75202947	5.52389633
H	1.98729936	12.20497252	4.00989426
N	0.80676611	9.09315215	4.71340538
N	0.55817770	8.66576042	2.57206537
C	-2.24634545	3.88701146	3.05271440
H	-3.24110320	3.68940166	3.47861626
H	-2.38609586	4.30984954	2.04960655

Coordinates for ^{Me}IPP_BF$_2$ (excited state, S_1)

C	-0.14283500	6.62089490	5.67722163
C	-0.63226462	5.38250506	6.15389002
H	-0.51539476	5.16575659	7.21425725
C	-1.24873061	4.49894506	5.29760456
H	-1.62505469	3.54806123	5.67531640
C	-1.42938815	4.81956540	3.92175818
H	-1.57785351	2.91860708	2.94097933
C	-0.92904416	6.02599953	3.43761583
H	-1.11322079	6.26798403	2.39669750
C	-0.24569180	6.94013270	4.26315823
C	0.35060233	8.15930166	3.84242580
C	1.26778000	10.18004901	4.07273362
C	1.11545938	9.93814374	2.69088186
C	1.43462949	10.71090324	1.54335076
H	1.87596325	11.69607690	1.66635527
O	0.40316532	7.43927107	6.55645070
C	1.18328326	10.13146576	0.26815998
B	0.84208043	8.84406066	6.28088764
H	1.41634320	10.69503565	-0.63640728
C	0.66256723	8.85139722	0.16854290
F	2.14944980	8.99614083	6.77518471
H	0.49346149	8.38842129	-0.80381818
C	0.33528434	8.08891656	1.32029456
F	-0.04015633	9.72600757	6.93847548
H	0.08768731	7.03879053	1.28938044
C	1.85255226	11.37428200	4.72362816
H	2.93864522	11.25330729	4.86904319
H	1.40423473	11.55095962	5.70835108
H	1.69687116	12.26232615	4.09870467
N	0.81477967	9.08009690	4.74592985
N	0.55999243	8.66981004	2.57049972
C	-2.14292336	3.86053878	3.02040789
H	-3.12907063	3.59809211	3.43126313
H	-2.27868694	4.26763792	2.01213938

Coordinates for ^{I}IPP_BF$_2$ (ground state, S_0)

C	-0.01250396	6.55726092	5.62452751
C	-0.44716430	5.31245611	6.09906890
H	-0.27172793	5.06802276	7.14635506
C	-1.09123517	4.40781972	5.26015855
H	-1.41718246	3.44429407	5.64946206
C	-1.32615886	4.75846951	3.92546493
I	-2.32930197	3.40864573	2.64321056
C	-0.89955056	5.98449574	3.42721927
H	-1.14255626	6.24522398	2.40269044
C	-0.20204512	6.89225270	4.25333542
C	0.34904043	8.15044105	3.81892162
C	1.26971042	10.18845963	4.05596220
C	1.13434933	9.94507557	2.69378085
C	1.44862624	10.68932056	1.53474492
H	1.87268099	11.68455680	1.65455308
O	0.60854736	7.38386389	6.48921755
C	1.22505069	10.14246482	0.29712788
B	0.76091051	8.81984388	6.26271746
H	1.46021777	10.70138292	-0.60678992
C	0.70095084	8.81955329	0.19575604
F	1.97278353	9.24356702	6.84308913
H	0.55282232	8.35599909	-0.77753089
C	0.38484801	8.09459111	1.30578369
F	-0.32236512	9.54976042	6.81895560
H	0.02369927	7.07625789	1.26064936
C	1.81492216	11.38974822	4.73799185
H	2.78475720	11.17278971	5.20618602
H	1.13649014	11.73809350	5.52758955
H	1.95324024	12.20144152	4.01541933
N	0.79605001	9.07986281	4.70665354
N	0.56546048	8.65362384	2.56238224

Coordinates for ^{I}IPP_BF$_2$ (excited state, S_1)

C	-0.09314023	6.57857589	5.65259081
C	-0.55717676	5.32978363	6.12914391
H	-0.44615961	5.11762195	7.19083333
C	-1.14367718	4.41989938	5.27663456
H	-1.49706625	3.46022173	5.64796467
C	-1.29746353	4.75966133	3.91094246
I	-2.24250474	3.41233434	2.63624134
C	-0.82965082	5.97160456	3.40276732
H	-1.00829799	6.20329439	2.35977670
C	-0.18138236	6.89606875	4.23800237
C	0.38697242	8.13373942	3.82418877
C	1.23762825	10.18371093	4.06765389
C	1.10918017	9.93900228	2.68767548
C	1.41175656	10.71965514	1.54154218
H	1.81818666	11.71948316	1.66848862
O	0.41701156	7.41055766	6.53728056
C	1.19054331	10.13647493	0.26437025
B	0.82596746	8.83010295	6.26686352
H	1.41150103	10.71000436	-0.63698618
C	0.71353888	8.83951014	0.15802242
F	2.12222386	9.01085198	6.78039520
H	0.56765430	8.37298853	-0.81630176
C	0.39981010	8.06601292	1.30528449
F	-0.08638578	9.68360671	6.92029847
H	0.18285193	7.00896905	1.26724239
C	1.77479823	11.39322079	4.73367554
H	2.84794266	11.28073185	4.95833361
H	1.25824009	11.58703769	5.68178306
H	1.65779157	12.26906892	4.08433703
N	0.80927332	9.06736874	4.73642112
N	0.59339910	8.64847462	2.55918764

Coordinates for ^{Cl}IPP_BF$_2$ (ground state, S_0)

C	0.00828536	6.54825550	5.61993551
C	-0.43332475	5.30599753	6.09524081
H	-0.24804325	5.05599018	7.13940931
C	-1.10047011	4.41300296	5.26201911
H	-1.44204055	3.45170754	5.64259262
C	-1.34514664	4.77679005	3.93467297
Cl	-2.23195708	3.68073857	2.88328079
C	-0.90521621	5.99279619	3.42792216
H	-1.15914374	6.24882426	2.40464722
C	-0.18940178	6.88801842	4.25096799
C	0.36413486	8.14586022	3.81833247
C	1.26136015	10.19356925	4.05646983
C	1.13011502	9.94836272	2.69358612
C	1.43713743	10.69459277	1.53362960
H	1.84594250	11.69647083	1.65200954
O	0.64450475	7.36935596	6.47992716
C	1.22811379	10.13996324	0.29653812
B	0.78049873	8.80939360	6.26383830
H	1.45903410	10.69966423	-0.60807654
C	0.72717928	8.80761469	0.19628948
F	1.99321532	9.24162181	6.83501604
H	0.59342993	8.33857391	-0.77652464
C	0.41627042	8.08134942	1.30723374
F	-0.30487004	9.52483713	6.83248689
H	0.07315592	7.05624719	1.26568131
C	1.78800543	11.40309461	4.73903136
H	2.76043315	11.20143697	5.20885395

Coordinates for ^{Cl}IPP_BF$_2$ (excited state, S_1)

C	-0.08347433	6.57874311	5.65556271
C	-0.53514238	5.32387282	6.12685639
H	-0.41570765	5.10546012	7.18620505
C	-1.12485986	4.41767200	5.27297673
H	-1.47807222	3.45376365	5.63252256
C	-1.29044359	4.76808118	3.91254354
Cl	-2.09688870	3.65396066	2.85465571
C	-0.83182367	5.97517859	3.40184975
H	-1.02460125	6.20179335	2.36043112
C	-0.18014400	6.89975289	4.24055018
C	0.38906670	8.13270383	3.82892247
C	1.24532216	10.17852264	4.07163619
C	1.11303970	9.93448380	2.68738555
C	1.41880654	10.71753803	1.54418126
H	1.82702080	11.71637363	1.67166433
O	0.42653523	7.41166652	6.54048005
C	1.19207161	10.13414307	0.26656323
B	0.79993573	8.84046362	6.27580121
H	1.41172667	10.70787028	-0.63495106
C	0.71255079	8.83915029	0.15940264
F	2.07406774	9.06351889	6.82195618
H	0.56167067	8.37482845	-0.81501193
C	0.40264377	8.06444522	1.30952323
F	-0.15606890	9.67422407	6.88951635
H	0.18042784	7.00850330	1.27439252
C	1.78680509	11.38844211	4.72984122
H	2.87015194	11.28840220	4.90895495
H	1.30708579	11.56323394	5.70020945
H	1.63355887	12.26879840	4.09406023
N	0.81846135	9.06525464	4.73972754
N	0.59719748	8.65003258	2.55883657

Coordinates for ^{Br}IPP_BF$_2$ (ground state, S_0)

C	-0.00619190	6.55629821	5.62522873
C	-0.44122560	5.31234243	6.10252150
H	-0.26465721	5.06831406	7.14949407
C	-1.08818688	4.40803083	5.26602605
H	-1.42152789	3.44404195	5.64685226
C	-1.31788257	4.76776235	3.93659499
Br	-2.24148987	3.54790801	2.77617434
C	-0.89463063	5.98683891	3.42775416
H	-1.14234635	6.23772311	2.40202738
C	-0.19719180	6.89289721	4.25400126
C	0.35234762	8.15081371	3.81852433
C	1.26856477	10.18974798	4.05566207
C	1.13436256	9.94575654	2.69287943
C	1.44872523	10.69039269	1.53405641
H	1.87205822	11.68595892	1.65429175
O	0.61756082	7.38223288	6.48831136
C	1.22776966	10.14266906	0.29603334
B	0.76129556	8.81946604	6.26455562
H	1.46440059	10.70128804	-0.60781174
C	0.70441542	8.81941589	0.19435250
F	1.96925251	9.25054409	6.84582795
H	0.55603944	8.35648507	-0.77936056
C	0.38825974	8.09398188	1.30449636
F	-0.32817405	9.54123332	6.81737311
H	0.02626872	7.07590652	1.25807184
C	1.81055110	11.39183251	4.73866879
H	2.77944773	11.17542890	5.20922133
H	1.12948392	11.73947688	5.52638743
H	1.94989448	12.20378920	4.01648387
N	0.79742360	9.08070177	4.70631816
N	0.56912231	8.65285865	2.56134436

Coordinates for ^{Br}IPP_BF$_2$ (excited state, S_1)

C	-0.08417757	6.58131188	5.65719952
C	-0.52847102	5.32474047	6.13008903
H	-0.40707480	5.10596609	7.18905894
C	-1.11364347	4.41508496	5.27659280
H	-1.45883357	3.44839576	5.63646027
C	-1.28039945	4.76942400	3.91900706
Br	-2.14767860	3.54396997	2.76223492
C	-0.83403809	5.97769225	3.40502057
H	-1.02954918	6.20689943	2.36469939
C	-0.18339569	6.90364825	4.24277116
C	0.38450604	8.13532250	3.82783993
C	1.25439392	10.17504227	4.06937923
C	1.11810037	9.93257811	2.68571583
C	1.42747542	10.71411823	1.54243964
H	1.84394850	11.70947046	1.67041976
O	0.42374006	7.41593978	6.54148028
C	1.19200938	10.13513415	0.26484780
B	0.78166417	8.84752322	6.27456028
H	1.41357769	10.70855991	-0.63640014
C	0.70018250	8.84479512	0.15708454
F	2.04248073	9.09031125	6.84140602
H	0.54092634	8.38407815	-0.81774108
C	0.38781847	8.07083400	1.30705106
F	-0.19750996	9.67167390	6.86544979
H	0.15268303	7.01789522	1.26926506
C	1.80387113	11.37932611	4.73056584
H	2.88262236	11.26325544	4.92662429
H	1.31271971	11.56324827	5.69368905
H	1.67215195	12.25984317	4.09057723
N	0.82200797	9.06436834	4.73789423
N	0.59163091	8.65168690	2.55708016
